# School-related sedentary behaviours and indicators of health and well-being among children and youth: a systematic review

**DOI:** 10.1186/s12966-022-01258-4

**Published:** 2022-04-05

**Authors:** Nicholas Kuzik, Bruno G. G. da Costa, Yeongho Hwang, Simone J. J. M. Verswijveren, Scott Rollo, Mark S. Tremblay, Stacey Bélanger, Valerie Carson, Melanie Davis, Susan Hornby, Wendy Yajun Huang, Barbi Law, Jo Salmon, Jennifer R. Tomasone, Lucy-Joy Wachira, Katrien Wijndaele, Travis J. Saunders

**Affiliations:** 1 Healthy Active Living and Obesity Research Group, Children’s Hospital of Eastern Ontario Research Institute, Ottawa, Canada; 2 Department of Pediatrics, Faculty of Medicine, University of Ottawa, Ottawa, Canada; 3 School of Physical & Health Education, Nipissing University, North Bay, Canada; 4 Faculty of Kinesiology, Sport, and Recreation, University of Alberta, Edmonton, Canada; 5 Institute for Physical Activity and Nutrition, Deakin University, Geelong, Australia; 6 Department of Health Sciences, Carleton University, Ottawa, Canada; 7 Département de Pédiatrie, Faculté de Médicine, Université de Montréal and CHU Sainte Justine, CIRENE (Centre Intégré du Réseau en Neurodéveloppement de L’Enfant), Montréal, Quebec Canada; 8 Physical and Health Education (PHE) Canada, Ottawa, Canada; 9 Pan-Canadian Joint Consortium for School Health (JCSH), Summerside, Canada; 10 Department of Sport, Physical Education and Health, Hong Kong Baptist University, Hong Kong, China; 11 School of Kinesiology and Health Studies, Queen’s University, Kingston, Canada; 12 Physical Education, Exercise and Sports Science, Kenyatta University, Nairobi, Kenya; 13 MRC Epidemiology Unit, University of Cambridge, Cambridge, UK; 14 Department of Applied Human Sciences, University of Prince Edward Island, Charlottetown, Canada

**Keywords:** Sedentary Behaviour, School, Children, Youth, Adolescent, Systematic Review

## Abstract

**Background:**

The purpose of this systematic review was to examine the associations between school-related sedentary behaviours and indicators of health and well-being in children and youth (~ 5–18 years) attending school.

**Methods:**

This review was conducted to inform the development of School-Related Sedentary Behaviour Recommendations. Peer-reviewed, published, or in-press articles in English were included. Reviews, meta-analyses, and case studies were excluded; all other study designs were eligible. Further, articles had to meet the a priori study criteria for population, intervention, comparator (PROSPERO ID: CRD42021227600). Embase, MEDLINE® ALL, and PsycINFO were searched. Risk of bias was assessed for individual experimental studies using the Cochrane risk of bias assessment tool, and in observational studies based on the GRADE framework and in line with previous systematic reviews examining sedentary behaviours in children. Overall quality of evidence was assessed using the GRADE framework for each outcome category and study design. Results were synthesized narratively, grouped by study design and outcome category. Further, several high-level summaries were conducted to help interpret results.

**Results:**

Evidence was synthesized from 116 reports, including 1,385,038 participants and 1173 extracted associations. More school-related sedentary behaviour was favourably associated with nearly one-third of extracted associations for cognitive (33%) and social-emotional (32%) indicators (e.g., less anxiety), but unfavourably associated with other movement behaviours (e.g., less physical activity) (35%). Active lessons were favourable (72%), compared to more school-related sedentary behaviours, when examining associations for all health and well-being indicators. More homework was favourable across all health and well-being indicators in 4% of extracted associations for primary school children, and 25% of extracted associations for secondary school children. However, ≥2 h/day of homework appeared to be unfavourable for health and well-being. Limitations for synthesized studies included generally low quality of evidence and a lack of studies in South American, African, or low-middle income countries.

**Conclusions:**

Findings can help inform policy makers, schools, and teachers, regarding the amount of homework assigned and the introduction of active lessons into the classroom to enhance health and well-being of children. More research is needed examining school-related sedentary behaviours and indicators of health and well-being in low- and middle-income countries.

**Supplementary Information:**

The online version contains supplementary material available at 10.1186/s12966-022-01258-4.

## Background

Sedentary behaviour is defined as any waking behaviour characterized by an energy expenditure ≤1.5 metabolic equivalents (METs) while in a sitting, reclining, or lying posture [1]. Accumulating high levels of sedentary behaviour is unfavourably associated with a breadth of health and well-being outcomes in school-aged children and youth or adolescents [2, 3]. However, according to an estimated global average, children accumulate 8 h/day of sedentary time [4], which represents approximately 50% of the waking day. Thus, children’s health and well-being may be at risk due to excessive time spent engaged in sedentary behaviours.

In line with the public health concerns surrounding children’s sedentary behaviours, Canada and Australia released 24-h movement behaviour guidelines for children and youth or young-people that included specific sedentary behaviour benchmarks [5, 6]. Specifically, it was recommended that children and youth should spend no more than 2 h/day engaged in recreational screen time and sitting for extended periods should be limited. These recommendations were informed by Carson et al.’s [2] systematic review of 235 studies, which included some studies examining school-related sedentary behaviours [7–12]. However, recommendations specifically for the school setting were not made since this was not an objective of the review.

The school is recognized as an important setting for promoting children’s health and well-being, based on the capacity of a school to incorporate health- and well-being-related curricula, establish a health- and well-being-related culture, and engage sources outside of the school that influence children’s behaviours (e.g., families, communities) [13]. Further, children spend a large amount of time in school, and the net global school attendance rates are 89% for primary (~ 5–12 years) and 66% for secondary (~ 13–17 years) school-aged children [14, 15]. Evidence indicates that children spend most of their school day sedentary, with one meta-analysis estimating that on average 63% of the school-day is spent sedentary for children and adolescents in the United States [16]. Further, sedentary behaviour in school directly accounted for an average of ~ 40% of total weekday sedentary behaviour in a sample of Spanish children and adolescents [17].

Schools, and policy makers, also dictate the volume of homework assigned to children, which ranges from 3 to 10 h/week of sedentary time according to global averages for 15-year-olds [18]. Considering the amount of sedentary time accumulated in schools, and assigned by schools, sedentary behaviour recommendations specific to the school setting may be important and relevant for children’s health and well-being. However, the development of school-related sedentary behaviour recommendations is precluded by the lack of a comprehensive literature synthesis examining the relationship between school-related sedentary behaviours and indicators of health and well-being.

Several previous reviews have examined aspects of school-related sedentary behaviours and indicators of health and well-being [19–26]. However, the scope of these reviews were narrow for included: study designs, health and well-being indicators, and school-related sedentary behaviours. For instance, these reviews have only examined experimental study designs. Some reviews examined any school-related intervention [19, 23], while the others were specific to recess [25], classrooms [22, 26], or standing desks in particular [20, 21, 24]. The two reviews that examined any school-related interventions were specific to adiposity indicators in primary school-aged children [23], and physical activity in older adolescents [19]. Further, of the reviews examining a broad spectrum of health and well-being indicators, two were specific to standing desk interventions [20, 24] and the other was specific to recess [25]. Collectively, these reviews have not provided an up-to-date and exhaustive overview of the associations between school-related sedentary behaviours and health and well-being indicators.

Based on the lack of representation across study designs, health and well-being indicators, and school-related sedentary behaviour exposures in previous systematic reviews, a comprehensive systematic review is needed, that builds on Carson et al.’s [2] review, to inform the development of school-related sedentary behaviour recommendations. Therefore, the objective of this systematic review was to comprehensively examine the associations between school-related sedentary behaviours and indicators of health and well-being in children and youth (~ 5–18 years) attending school. Further objectives of this study included examining differences in associations across school-related sedentary behaviour exposure types (e.g., homework, sedentary time) and age groups, as well as examining any dose-response associations for school-related sedentary behaviours with health and well-being indicators.

## Methods

### Context

This systematic review was conducted to act as a source of evidence informing the development of the School-Related Sedentary Behaviour Recommendations, conducted by members of the Sedentary Behaviour Research Network (SBRN). Members of the SBRN Recommendations Steering Committee and an international expert panel met to determine key methodological decisions when conceptualizing this review. Details of the final guidelines are available elsewhere [27]. A summary of the methodology specific to the current review is presented below.

### Protocol and registration

This systematic review was registered with the international prospective register of systematic reviews (PROSPERO; Registration ID: CRD42021227600) and followed the Preferred Reporting Items for Systematic Reviews and Meta-Analyses (PRISMA) guidelines [28].

### Eligibility criteria

Only peer-reviewed, published, or in-press articles in English were included. Reviews, meta-analyses, and case studies were excluded, but all other study designs were eligible. Further, articles had to meet the a priori study criteria for population, intervention, comparator, and outcome (PICO) [29] in line with the Grading of Recommendations Assessment, Development, and Evaluation (GRADE) framework [30, 31].

#### Population (participants)

Apparently healthy (i.e., general populations, including those with overweight/obesity, but not samples exclusively with a diagnosed medical condition) children and youth (~ 5–18 years) attending primary or secondary school. For studies measuring multiple time points (e.g., longitudinal), school attendance was needed for at least one measurement time point. The following post-hoc sample size exclusion criteria were imposed in line with previous systematic reviews [2, 32]: experimental/intervention studies needed a minimum sample size of ≥30 participants, while observational studies needed a minimum sample size of ≥300 participants.

#### Intervention (exposures)

Duration, patterns, and types of school-related sedentary behaviours. Sedentary behaviour is defined according to the SBRN as any waking behavior characterized by an energy expenditure ≤1.5 METs, while in a sitting, reclining, or lying posture [1]. For this review, the operational definition of sedentary behaviours included behaviours typically involving sedentary postures and low energy expenditure (e.g., homework, screen time [iPad/tablet/touch-screen, smart phone], time spent sitting). All sedentary behaviours needed to be school-related, which refers to sedentary behaviours occurring during school hours (e.g., classroom, recess) or outside of school hours but influenced by the school (e.g., homework, studying). Traditional class time was assumed to be sedentary, and activities that could displace sedentary behaviours (e.g., adding more physical education (PE) classes to the schedule) were included as a proxy for sedentary behaviour reduction. Sedentary behaviours were not operationally defined as failing to meet physical activity guidelines [1]. For experimental studies, interventions that targeted multiple health behaviours (e.g., standing time and nutrition) were not included. If possible, results were to be separated based on the context of school-related sedentary behaviours as: outside of school hours (e.g., homework), during school instructional time (e.g., classroom), and during school free-time (e.g., recess). However, to better align with the extracted school-related sedentary behaviour exposures, post-hoc categories were created for: active breaks, active lessons, additional physical activity, homework, recess/PE, screen time, standing desks, and sedentary time.

#### Comparator

Various durations, patterns, or types of school-related sedentary behaviours. However, a comparator or control group was not required.

#### Outcomes (indicators)

Outcome or indicator categories were selected based on previous systematic reviews [2, 33], and through expert input and consensus. Based on the GRADE framework, health and well-being outcome categories were ranked as “critical” or “important” by the steering committee and expert panel, since only critical or important outcomes should be used to inform guideline recommendations [34]. Critical outcomes included: adiposity indicators (e.g., body mass index, waist circumference, skinfolds, bio-electrical impedance analysis), biomarkers (e.g., lipid profile, insulin, glucose, blood pressure), cognitive indicators (e.g., academic achievement, executive functions, literacy), musculoskeletal growth (e.g., bone mineral density, fat free mass, height), risks (injury)/harm (e.g., “text neck”/anterior head syndrome, eyestrain, headaches), and social-emotional indicators (e.g., classroom time on task, prosocial behaviour, sociability, self-esteem). Important outcomes included: fitness (e.g., grip strength, shuttle run, flexibility) and other movement behaviours (e.g., physical activity, sleep, non-school-related sedentary behaviours).

### Information sources and search strategy

This review adopted and modified a previous search strategy, developed with and peer-reviewed by academic librarians with expertise in systematic review search strategies [2]. Search terms were updated to include sedentary behaviours that were not common or did not exist in 2016 (e.g., Zoom), and to include terms specific to school-related sedentary behaviours (e.g., homework). Search strategies were modified specifically for the databases Ovid Embase, Ovid MEDLINE® ALL, and Ovid PsycINFO. A date limit was used to exclude articles published before January 1, 2014 to reduce overlap with the previous systematic review [2]. The most recent search was conducted on January 7, 2021. For the full search strategy, see Additional File 1. Records were imported into Covidence (Veritas Health Innovation, Melbourne, Australia) and deduplication was completed before screening for eligibility.

### Study selection

At level 1 screening, titles and abstracts of all potentially relevant articles were reviewed by the lead author (NK) and one other independent reviewer (BC, YH, or SV) in Covidence. Eligible articles meeting the screening criteria by either reviewer proceeded to level 2 full-text screening. The lead author (NK) and another independent reviewer (BC, YH, or SV) screened the full-text articles for inclusion or exclusion. Discrepancies in article inclusion or exclusion were resolved through discussion and consensus between the 2 reviewers, or by including a third reviewer (TS) to reach consensus.

### Data collection process and data items

Data were extracted from eligible articles into Google Sheets templates. All studies were extracted by one reviewer and verified by another reviewer, with one reviewer (NK) extracting or verifying each study and the other reviewers (BC, YH, or SV) independently performing the reciprocal extraction or verification. For each study, descriptive characteristics were extracted including author, publication year, country, study design, and sample size. Details for the exposure, outcome, and study results were extracted for each study. When studies reported results from multiple models (e.g., bivariate and adjusted linear regression models), the most fully adjusted results were used to summarize findings. However, if models with similar covariates were reported (e.g., adjusted for BMI, and adjusted for body fat percentage), results from both models were extracted for comparison. Statistical significance of extracted results was defined as *p* < 0.05 regardless of how individual studies defined statistical significance.

### Risk of bias in individual studies and across studies

Risk of bias was assessed or verified for individual studies by one reviewer (NK) and the other reviewers (BC, YH, or SV) independently performed the reciprocal assessment or verification. To assess risk of bias for individual experimental studies, the Cochrane risk of bias assessment tool was used [35]. For assessing risk of bias in observational studies, criteria were determined based on recommendations for types of characteristics to examine from the GRADE framework, and in line with similar systematic reviews examining sedentary behaviours in children [2, 32]. The assessed criteria consisted of the following domains: selection bias, performance bias, selective reporting bias, detection bias, attrition bias, and other biases (e.g., inadequate control for key confounders). The results for study-level risk of bias can be found in Additional File 3. The GRADE framework was used to assess the overall quality of evidence for each outcome category and study design [36]. Quality of evidence was ranked as “high”, “moderate”, “low”, or “very low”, corresponding to the confidence that the true effect aligns with the estimated effect (e.g., very low = true effect is distinctly different from the estimated effect; high = confident that the true effect is close to the estimated effect) [36]. The ranked quality of evidence started at high for randomised trials and low for other study designs. Quality of evidence was downgraded if serious limitations were seen in the domains of risk of bias, inconsistency, indirectness, or imprecision. If no downgrades occurred then quality of evidence could be upgraded based on large magnitudes of effect, dose-response gradients, or sufficiently controlling for residual confounding. Quality of evidence ratings were performed by one reviewer and presented to the broader expert panel for consensus.

### Synthesis of results

Due to heterogeneity across school-related sedentary exposures and outcomes, meta-analyses were not conducted. Instead, narrative syntheses were performed. Specifically, extracted results were coded in the direction of null, favourable (i.e., desired or beneficial), or unfavourable (i.e., undesired or adverse) based on the significance and direction of an association between the school-related sedentary behaviour exposure and the outcome. For consistency, result directions (e.g., favourable, unfavourable) were reported as the relationship between sedentary behaviour and the indicator of health and well-being. As previously discussed, traditional class time was assumed to be sedentary, and activities that could displace sedentary behaviours (e.g., adding more physical education (PE) classes to the schedule) were included as a proxy for sedentary behaviour reduction. For instance, if higher durations of recess were associated with higher grades, this was described as more sedentary behaviour being unfavourable for academic achievement. In contrast, if a longitudinal study found that more homework was associated with higher grades, this was described as more homework being favourable for academic achievement.

For each health and well-being indicator category, when all extracted results for a study were in the same direction, this study was classified as consistently null, favourable, or unfavourable. When the direction of findings for extracted results for a study were not consistent (e.g., one study finding more sedentary behaviour was favourable and null for two extracted results in the same health and well-being indicator category), the study was classified as mixed. When mixed results were observed, attempts were made to explain the inconsistent findings (e.g., dose-response relationships). Studies comparing different types of sedentary behaviours (e.g., screen vs paper-based learning) were not coded as favourable or unfavourable with sedentary behaviour in general, but instead framed relative to each of the specific sedentary behaviours being examined (e.g., sedentary game favourable for cognitive indicators when compared to sedentary lesson). Summary tables also included subsections for each category of school-related sedentary behaviours. To assist with the interpretation of results, high-level summaries of results that omitted the mixed category by counting the frequency of individual results being null, favourable, or unfavourable were also generated. High-level results were summarized by outcome and exposure categories, as well as the age categories of primary school-aged (~ 5–12 years) and secondary school-aged (~ 13–18 years) children. When sample ages spanned across primary and secondary school-age ranges, results were omitted from the age sub-categorizations.

## Results

### Study selection

**Fig. 1:**
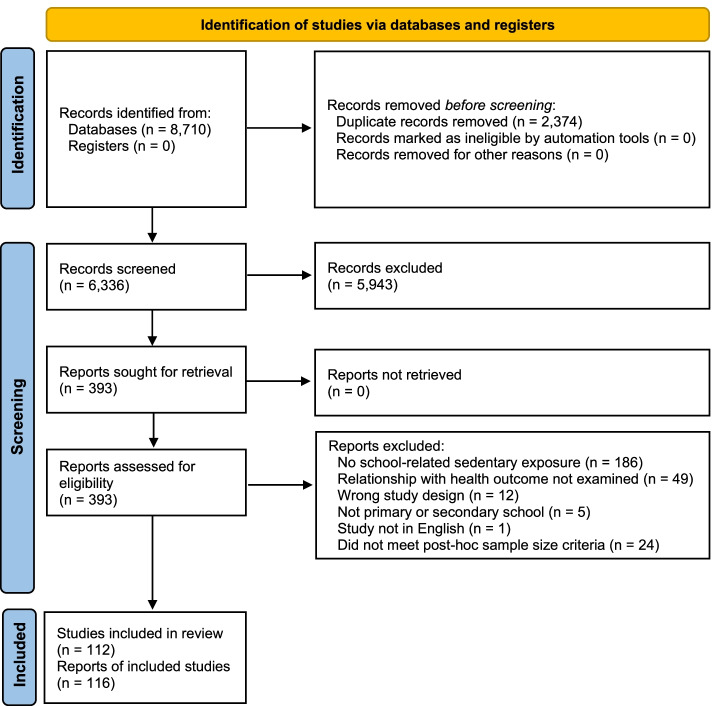
PRISMA Flowchart

Figure 1 illustrates the PRISMA flowchart for included studies. After screening, 116 studies were included and had all relevant data extracted and quality of evidence rated for the qualitative synthesis.

### Study characteristics

**Fig. 2:**
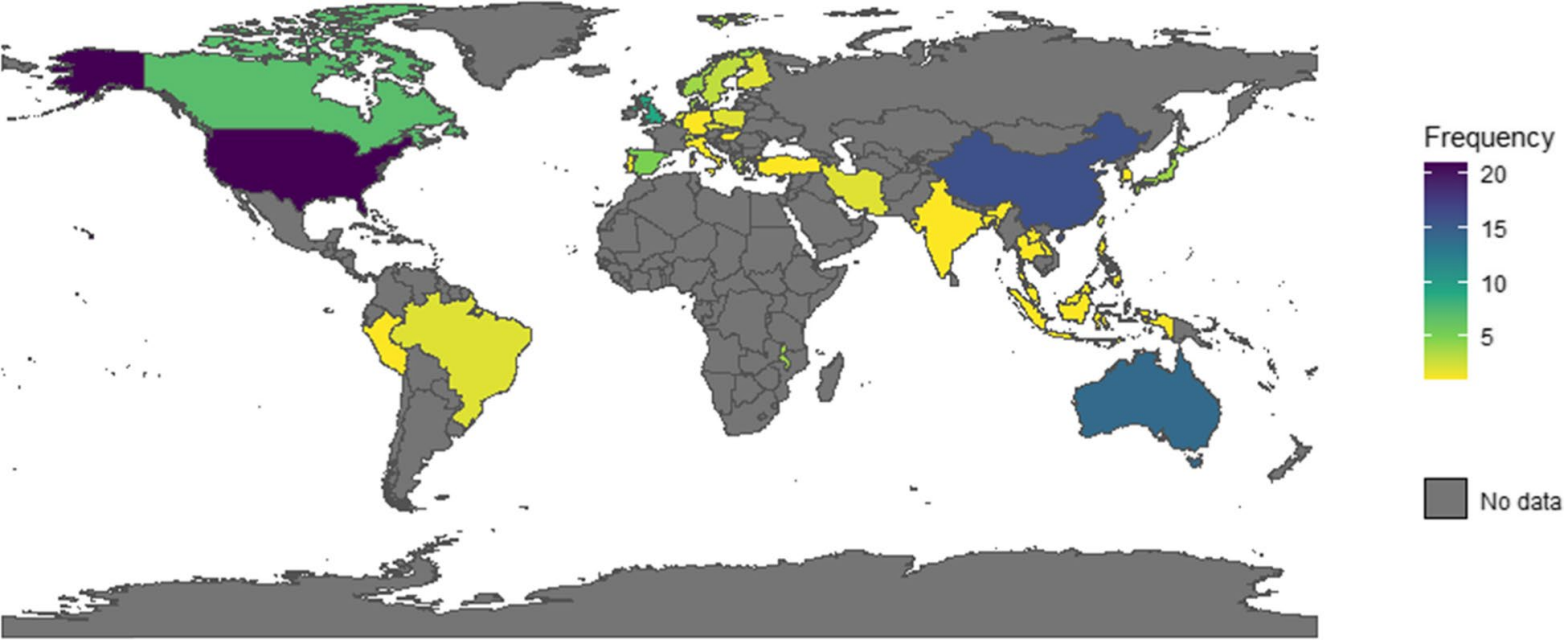
Number of Studies by Country

The 116 included articles, including 112 samples (or unique studies), are summarized by outcome category in Additional File 2; Supplementary Tables 1–8. For the articles from the same study [37–43], there was only one instance of the same results being presented in more than one article. Specifically, two articles from one study reported on the same associations for other movement behaviours [37, 43], so results specific to other movement behaviours were only used from the first study [43]. Though, both studies contributed unique findings to other health and well-being outcome categories, so study exclusion was not necessary. Extracted data across study designs included 1,385,038 participants, of which 1,327,091 were from unique samples. Of the 116 articles, 5 examined multiple countries, including Australia and the United Kingdom [44]; Czech Republic and Poland [45]; Fiji, Kiribati, Samoa, Solomon Islands, Tonga, and Vanuatu [46]; Indonesia, Lao PDR, Philippines, Thailand, and Timor-Leste [47]; Belgium, Greece, Hungary, Netherlands, and Switzerland [48]. Data were collected from a total of 43 countries, with the most frequent being the United States (*n* = 19/131), China (*n* = 16/131), and Australia (*n* = 14/131), and the most frequent continents being Europe (*n* = 45/131), Asia (*n* = 35/131), and North America (*n* = 25/131) (See Fig. 2). As well, according to World Bank income classifications, data were collected from high (70.8%), upper-middle (17.5%), lower-middle (8.0%), and low (3.6%) income countries. Baseline mean ages ranged from 6 to 17 years and from school grades 1 to 12. Experimental study designs were used in 44 studies, including clustered RCTs (*n* = 20), RCTs (*n* = 3), cross-over trials (*n* = 5), and non-randomised interventions (*n* = 16). Observational study designs were used in 79 studies (longitudinal [*n* = 14] and cross-sectional [*n* = 65]). Of those studies, five articles contained results for two study designs (cross-sectional and longitudinal: *n* = 3; non-randomised intervention and longitudinal: *n* = 1; non-randomised intervention and cross-sectional: *n* = 1) and two articles contained the results of two different samples/experiments (non-randomised interventions: *n* = 1; clustered RCTs: *n* = 1). The school-related sedentary exposures (including those that imply the displacement of sedentary behaviour) included homework (*n* = 57), recess/PE (*n* = 16), standing desks (*n* = 12), sedentary time (*n* = 9), active breaks (*n* = 9), additional physical activity (*n* = 8), screen time (*n* = 8), and active lessons (*n* = 7)—some studies measured multiple exposures.

### Synthesis of results

#### Critical outcomes

##### Adiposity indicators

Thirty-two studies examined the association between school-related sedentary behaviours and adiposity indicators (See Additional File 2, Supplementary Table 1 for the individual studies). The study designs for these articles were clustered RCT (*n* = 4), non-randomised intervention (*n* = 4), longitudinal (*n* = 3), and cross-sectional (*n* = 21). For a summary of the measured adiposity indicators, see Table 1.

**Table 1:** Summary of results for adiposity indicators organized by study design

No. of participants (No. of studies)	Design	Risk of bias	Inconsistency	Indirectness	Imprecision	Other	Absolute effect	Quality
1060 (4) [49–52]	Clustered RCT	No serious risk of bias	No serious risk of inconsistency	Very serious risk of indirectness^a^	No serious risk of imprecision	None	**Overall:** • **4/4** studies reported **null** findings **Additional PA:** • **2/2** studies reported **null** findings [49, 50] **Recess/PE:** • **1/1** studies reported **null** findings [51] **Standing desk:** • **1/1** studies reported **null** findings [52]	Low
728 (4) [53–56]	Non-Randomised Intervention	Serious risk of bias^b^	No serious risk of inconsistency	Serious risk of indirectness^c^	No serious risk of imprecision	None	**Overall:** • **2/4** studies reported **null** findings • **1/4** studies reported more sedentary behaviour **unfavourable** for health • **1/4** studies reported **mixed** findings • **1:** More sedentary behaviour null and unfavourable for health **Active Breaks:** • **1/1** studies reported more sedentary behaviour **unfavourable** for health [56] **Recess/PE:** • **1/1** study reported **null** findings [53] **Standing desk:** • **1/2** studies reported **null** findings [54] • **1/2** studies reported **mixed** findings • **1:** More sedentary behaviour null for BMI, and unfavourable for waist circumference [55]	Very Low
2330 (3) [57–59]	Longitudinal	Serious risk of bias^d^	No serious risk of inconsistency	No serious risk of indirectness	No serious risk of imprecision	None	**Homework:** • **1/3** studies reported **null** findings [57] • **1/3** studies reported more homework **favourable** for health [58] • **1/3** studies reported **mixed** findings^e^ • **1:** More homework null and unfavourable for health [59] • **1:** Mixed findings included **dose response** relationships with **unfavourable** associations for ≥3 h studying, and **null** for 1–3 h studying for obesity but not overweight [59]	Very Low
616,995^f^ (21) [38, 41, 46–48, 60–75]	Cross-sectional	Serious risk of bias^g^	No serious risk of inconsistency	Serious risk of indirectness^f^	No serious risk of imprecision	None	**Overall:** • **10/21** studies reported **null** findings • **4/21** studies reported more sedentary behaviour **unfavourable** for health • **7/21** studies reported **mixed** findings • **5:** More sedentary behaviour null and unfavourable for health • **2:** More sedentary behaviour null and favourable for health **Active breaks:** • **1/1** study reported **null** findings [60] **Additional PA:** • **1/1** study reported **null** findings [60] **Homework:** • **5/11** studies reported **null** findings [61–65] • **2/11** studies reported more homework **unfavourable** for health [41, 69] • **4/11** studies reported **mixed** findings^h^ • **4:** More homework null and unfavourable for health [38, 72–74] **Recess/PE:** • **2/5** studies reported **null** findings [47, 60] • **1/5** studies reported more sedentary behaviour **unfavourable** for health [70]^i^ • **2/5** studies reported **mixed** findings^j^ • **1:** More sedentary behaviour null and favourable for health [46] • **1:** More sedentary behaviour null and unfavourable for health [73] **Sedentary time:** • **3/6** studies reported **null** findings [66–68] • **1/6** studies reported more sedentary time **unfavourable** for health [71] • **2/6** studies reported **mixed** findings^k^ • **1:** More sedentary time null and favourable for health [75] • **1:** More sedentary time null and unfavourable for health [48]	Very Low

Mean age at baseline ranged from 7.7 to 15.0 years; when mean age was not reported age or grade range minimums were 10.0 years and grade 1 and range maximums were 16.0 years and grade 6. Study designs included clustered RCT, non-randomised interventions, and longitudinal with up to 7 years follow-up, and cross-sectional. Adiposity was assessed objectively by BMI, BMI percentiles (Centers for Disease Control [CDC], German national standards, and unreported), BMI z-scores (CDC, International Obesity Task Force [IOTF], World Health Organization [WHO]), fat mass index (combination of skinfolds, height, and weight), fat-free mass (bioelectrical impedance), sum of skinfolds, total body fat percentage (Bioelectrical impedance, dual energy x-ray absorptiometry, as well as a combination of skinfolds, height, and weight), trunk body fat percentage (dual energy x-ray absorptiometry), waist circumference, waist circumference z-scores (Cole), weight status (CDC, IOTF, WHO, Working Group on Obesity in China [WGOC], Korean Centers for Disease Control [KCDC]). Subjectively assessed weight status (Self-reported: IOTF, sample-specific z-score classification; proxy-reported: WGOC, WHO). Further two studies were unclear whether measurements were objective or subjective for BMI z-scores (WHO), and weight status (WHO)

^a^ None of the studies included a measure of school-related sedentary behaviours

^b^ 2/4 studies had high risk of performance bias based on the comparison groups being in the same school [54, 55]

^c^ 2/4 studies did not report school-related sedentary exposures [53, 55], while the other 2/5 studies found the interventions decreased sedentary behaviours [54, 56]

^d^ 3/3 studies were high risk for performance bias due to no demonstration of psychometric testing for subjective exposure measures

^e^ 1: Unfavourable for overall for obesity, as well unfavourable for ≥3 h/day of studying for obesity; null for 1–3 h/day for overweight and obesity status, and ≥ 3 h/day for overweight status

^f^ 1 study sampled 905 schools and approximated sample size based on teachers estimates (~ 524,700)

^g^ 11/21 studies were high risk of performance bias due to no demonstration of psychometric testing of subjective exposure measures

^h^ 4: Null & unfavourable [2: weekend homework = null, weekday homework = unfavourable [38, 72]; 1: unfavourable when comparing overweight to normal weight for boys, but null for all other weight class and gender comparisons [73]; 1: unfavourable for boys and boys stressed by homework, but null for boys not stressed by homework or any comparisons for girls [74]]

^i^ Unfavourable overall, but null for females when comparing never attend PE class to regularly attend PE class [70]

^j^ 1: Null & favourable [1: Favourable for overweight group, but null for obese and overweight/obese [46]]; 1: Null & unfavourable [1: Unfavourable when comparing overweight to normal weight for boys and girls, and overweight to underweight for girls, null for all other sex and weight category comparisons [73]]

^k^ 1: Null & favourable [1: Favourable for boys sedentary time during recess, but null for all other contexts and all contexts for girls [75]]; 1: Null & unfavourable [unfavourable for the Netherlands group, but null for all other countries [48]]

Among clustered RCT study designs, null findings were consistently reported in 4/4 studies [49–52]. The consistent null findings were reported for the school-related sedentary exposures of additional physical activity [49, 50], recess/PE [51], and standing desks [52]. Overall, the quality of evidence was rated as low due to a very serious risk of indirectness. For a description of quality of evidence assessments, see Table 1.

For non-randomised interventions, findings were consistently reported as null in 2/4 studies [53, 54], mixed in 1/4 studies [55], and more sedentary behaviour was unfavourable for adiposity indicators in 1/4 studies [56]. Organizing by categories of school-related sedentary behaviour exposures, consistent null findings were reported recess/PE [53], while more sedentary behaviour was unfavourable for adiposity indicators when compared to active breaks [56], suggesting a benefit for displacing sedentary behaviour with active breaks. For standing desks, 1/2 studies reported consistent null findings [54]. Overall, the quality of evidence was rated as very low due to serious risk of bias and serious risk of indirectness.

Among longitudinal studies, homework was the only school-related sedentary exposure. Findings were consistently reported as null in 1/3 studies [57] and more homework was favourable for adiposity indicators in 1/3 studies, indicating that higher levels of homework were associated with lower adiposity [58]. While findings were reported as mixed (i.e., null and unfavourable) in 1/3 studies, a dose response relationship partly explained the mixed findings with unfavourable associations seen for ≥3 h/day of studying time [59], suggesting that homework above that threshold was associated with higher adiposity. Overall, the quality of evidence was rated as very low due to serious risk of bias.

For cross-sectional studies, findings were consistently reported as null in 10/21 studies [47, 60–68], more sedentary behaviour was unfavourable for adiposity indicators in 4/21 studies [41, 69–71], and mixed for 7/21 studies [38, 46, 48, 72–75]. Organizing by categories of school-related sedentary behaviour exposures, consistent null associations were seen for active breaks [60] and additional physical activity [60]. More sedentary behaviour was unfavourable for adiposity indicators in 2/11 studies examining homework [41, 69], 1/6 studies examining sedentary time [71], and 1/5 studies examining recess/PE [70]. Overall, the quality of evidence was rated as very low due to serious risk of bias and serious risk of indirectness.

##### Biomarkers

A total of 4 studies examined the association between school-related sedentary behaviours and biomarkers (See Additional File 2, Supplementary Table 2 for the individual studies). Study designs included clustered RCTs (*n* = 2), non-randomised intervention (*n* = 1), and longitudinal (*n* = 1). For a summary of the measured biomarkers, see Table 2.

**Table 2:** Summary of results for biomarker indicators organized by study design

No. of participants (No. of studies)	Design	Risk of bias	Inconsistency	Indirectness	Imprecision	Other	Absolute effect	Quality
525 (2) [49, 51]	Clustered RCT	Serious risk of bias^a^	No serious risk of inconsistency	No serious risk of indirectness	No serious risk of imprecision	None	**Overall:** • **2/2** studies reported **null** findings **Additional PA:** • **1/1** study reported **null** findings [49] **Recess/PE:** • **1/1** study reported **null** findings [51]	Moderate
41 (1) [54]	Non-Randomised Intervention	Serious risk of bias^b^	No serious risk of inconsistency	No serious risk of indirectness	Very serious risk of imprecision^c^	None	**Standing desk:** • **1/1** study reported **null** findings [54]	Very Low
698 (1) [76]	Longitudinal	Serious risk of bias^d^	No serious risk of inconsistency	No serious risk of indirectness	Serious risk of imprecision^e^	None	**Homework:** • **1/1** study reported **null** findings [76]	Very Low

Mean age at baseline ranged from 6.7 to 11.5 years; when mean age was not reported one study had an age range of 11 to 12 years, while another study sampled from children in grades 1 and 5. Study designs included clustered RCT, non-randomised interventions, and longitudinal with up to 5 years follow-up. Biomarkers were assessed objectively by blood pressure (systolic, diastolic, and mean arterial blood pressures), fasting blood draws (glucose, insulin, high-density lipoprotein-cholesterol (HDL), low-density lipoprotein-cholesterol (LDL) and triglycerides) and composite cardiovascular risk scores (z-scores of waist circumference, blood pressure [mean of systolic and diastolic blood pressure z-scores], glucose, inverted HDL, and triglycerides)

^a^1/2 studies had intervention and control groups in the same school

^b^ 1/1 study had intervention and control group in the same school

^c^ Only one study, with a small sample size

^d^ Differences in measurement of blood pressure from baseline to follow-up

^e^ Only one study, but large sample size

Across all study designs and exposure categories, consistent null findings were observed. This included 2/2 clustered RCTs assessing additional physical activity [49] and recess/PE [51], 1/1 non-randomised intervention assessing standing desks [54], and 1/1 longitudinal study assessing homework [76]. The quality of evidence was rated as either moderate (clustered RCT) or very low (non-randomised intervention and longitudinal) due to serious risk of bias for all study designs, as well as very serious risk of imprecision (non-randomised intervention) and serious risk of imprecision (longitudinal).

##### Cognitive indicators

A total of 29 studies examined the association between school-related sedentary exposures and cognitive indicators (See Additional File 2, Supplementary Table 3 for the individual studies). The study designs for these articles were clustered RCT (*n* = 7), RCT (*n* = 3), cross-over trial (*n* = 2), non-randomised intervention (*n* = 3), longitudinal (*n* = 3), and cross-sectional (*n* = 11). One article contained two clustered RCT studies [77]. For a summary of the measured cognitive indicators, see Table 3.

**Table 3:** Summary of results for cognitive indicators organized by study design

No. of participants (No. of studies)	Design	Risk of bias	Inconsistency	Indirectness	Imprecision	Other	Absolute effect	Quality
3628 (7) [37, 43, 52, 77–79]	Clustered RCT	No serious risk of bias	No serious risk of inconsistency	Serious risk of indirectness^a^	No serious risk of imprecision	None	**Overall:** • **4/7** studies reported **null** findings • **1/7** studies reported **mixed** findings • **1:** More sedentary behaviour null and unfavourable for health • **2/7** studies compared **types** of sedentary behaviours **Active breaks:** • **1/1** study reported **null** findings [78] **Active lessons:** • **1/1** studies reported **null** findings [79] **Additional PA:** • **2/2** studies reported **null** findings [37, 43] **Screen time:** • **2/2** studies compared **types** of sedentary behaviours^b^ • **2:** Tablet was favourable for health compared to usual classroom in both experiments/samples [77]. **Standing desk:** • **1/1** study reported **mixed** findings^c^ • **1:** Null and unfavourable [52]	Moderate
615 (3) [80–82]	RCT	No serious risk of bias	No serious risk of inconsistency	No serious risk of indirectness	No serious risk of imprecision	None	**Overall:** • **1/3** study reported **mixed** findings • **1:** More sedentary behaviour null and unfavourable for health • **2/3** studies compared **types** of sedentary behaviours **Active breaks:** • **1/1** studies reported **mixed** findings • **1:** More sedentary behaviour null and unfavourable for health [Null compared to one activity break, but unfavourable compared to two activity breaks [80]] **Screen time:** • **2/2** studies compared **types** of sedentary behaviours^d^ • **2:** Mix of favourable and null associations for educational tablet groups compared to non-educational tablet and typical classroom groups [81, 82]	High
200 (2) [83, 84]	Cross-Over Trial	No serious risk of bias	No serious risk of inconsistency	No serious risk of indirectness	No serious risk of imprecision	None	**Screen Time:** • **2/2** studies compared **types** of sedentary behaviours • **1:** Paper-based favourable for health when compared to screen-based [83] • **1:** Paper-based favourable and null for health when compared to screen based [84]^e^	High
277 (3) [85–87]	Non-Randomised Intervention	Serious risk of bias^f^	No serious risk of inconsistency	No serious risk of indirectness	No serious risk of imprecision	None	**Overall:** • **2/3** studies reported **null** findings • **1/3** studies compared **types** of sedentary behaviours **Standing desk:** • **2/2** studies reported null findings [85, 86]^g^ **Screen time:** • **1/1** studies compared **types** of sedentary behaviours • **1:** Class-based educational video games were favourable for the subject Math but null for Danish [87]	Very Low
13,715 (3) [40, 88, 89]	Longitudinal	Serious risk of bias^h^	No serious risk of inconsistency	No serious risk of indirectness	No serious risk of imprecision	Dose Response	**Homework:** • **1/3** studies reported more homework **favourable** for health [88] • **2/3** studies reported **mixed** findings^i^ • **2:** More homework favourable and null for health • **2:** Mixed findings included **dose response** relationships with **favourable** associations for “high homework” levels and > 121 min/day, and **null** associations for “medium homework” levels, 61–90 min/day, and 90–120 min/day [40, 89]	Very Low
270,810 (11) [62, 63, 68, 90–97]	Cross-sectional	Serious risk of bias^j^	No serious risk of inconsistency	No serious risk of indirectness	No serious risk of imprecision	None	**Overall:** • **1/11** studies reported **null** findings • **1/11** studies reported more sedentary behaviour **unfavourabl**e for health • **6/11** studies reported more sedentary behaviour **favourable** for health • **3/11** studies reported **mixed** findings • **2:** More sedentary behaviour favourable and null for health • **1:** More sedentary behaviour favourable and unfavourable for health **Homework:** • **5/9** studies reported more homework **favourable** for health [62, 63, 92–94]^k^ • **1/9** studies reported more sedentary behaviour **unfavourable** for health [91] • **3/9** studies reported **mixed** findings^l^ • **2:** More sedentary behaviour favourable and null for health [95, 96] • **1:** More sedentary behaviour favourable and unfavourable for health [97] **Recess/PE:** • **1/1** studies reported **null** findings [90] **Sedentary time:** • **1/1** studies reported more sedentary time **favourable** for health [68]	Very Low

Mean age at baseline ranged from 7.0 to 14.9 years; when mean age was not reported age or grade range minimums were 10.0 years and grade 3 and range maximums were 18.0 years and grade 12. Study designs included clustered RCT, RCT, cross-over trials, non-randomised interventions, and longitudinal with up to 4 years follow-up, and cross-sectional. Cognitive indicators were assessed objectively by academic achievement (grade point average for Japanese, Mathematics, Social Studies, Sciences, English, Music, Arts, and Home Economics/Vocational Technology; Norwegian standardized national tests; General Certificate of Secondary Education exams scores (GCSEs); Grade point average; Language grade; Math grade; Math & language grade; Citizenship grade; Math grade; Spanish grade; Sciences grade; Average of Chinese, mathematics, English, and science standardized test scores; National Assessment Program - Literacy and Numeracy (NAPLAN); and Norwegian standardized national tests -Reading, English, and Numeracy), cognitive flexibility (Trail Making Test part B, Verbal Fluency, Dimensional Change Card Sort Test [NIH Toolbox]), episodic memory (Picture Sequence Test [NIH Toolbox], Wechsler Memory Scale [WMS-R] Logical Memory subtest], executive functions (mean of standardized scores for Trail Making Test part B, Verbal Fluency test, Stroop Color Word test, and Digit Span test), inhibitory control (Stroop test, Eriksen Flanker reaction time, Flanker Test [NIH Toolbox]), manual processing speed (Single-Finger-Tapping task), math abilities (study specific quiz, Early Grade Mathematics Assessment [EGMA], Heidelberger Rechen Test 1–4 [HRT]), mathematics conceptual understanding (study specific quiz), math curriculum knowledge (quiz items based on educational app), maths curriculum knowledge generalization (quiz items based on educational app), memory (study specific quiz), non-verbal reasoning (Matrix Reasoning test [Wechsler Intelligence Scale for Children fourth edition]), processing speed (Pattern Comparison Test [NIH Toolbox]), reading abilities (Early Grade Reading Assessment [EGRA]-Chichewa), selective attention (Sky Search’ subtest of the ‘Test of Selective Attention in Children’ [TEA-Ch]), short-term memory (Forward Spatial Span task, and Forward Digit Span task), verbal reasoning and verbal knowledge (British Abilities Scale Verbal Similarities), visual attention (Speeded Search task), and working memory (Digit Span test [Wechsler Intelligence Scale for Children fourth edition], Backward Digit Span task, Corsi Block Tapping test, and Figural Intersections task). Cognitive indicators were assessed subjectively through self-report of academic achievement (letter grades, percent scores, number of failed subjects, or relative to peers for English, Math, and across all subjects) and academic performance (ability to understand school lessons) or number of failed subjects overall), as well as teacher-report of academic achievement (general performance in Math and Danish)

^a^ Only 3/7 studies demonstrated an intervention effect for decreasing school-related sedentary behaviours

^b^ 1 study, but included 2 experiments that were treated as 2 separate studies

^c^ 1: Null & unfavourable [unfavourable for working memory, but null for non-verbal reasoning [52]]

^d^ 1: No difference for younger children or when split by gender, but educational tablet group was generally more favourable compared to non-educational tablet and typical classroom groups [81]; 1: educational tablet group favourable for math test score and visual attention when compared to non-educational tablet and typical classroom groups, but no differences between groups for short-term memory and manual processing speed [82]

^e^ 1: Paper-based favourable for most outcomes, but no difference for younger children and when compared to mobile screens [84]

^f^ 3/3 studies had high risk of reporting bias based on insufficient details reported for study variables

^g^ 1: Study reported a favourable finding at 4 months, but null at 8 months [85]

^h^ 3/3 studies had high risk for attrition bias with included participants differing from excluded participants for key variables

^i^ 2: Favourable when comparing highest levels of homework (i.e., High homework levels and > 121 min/day) [1: only for English not Math [40]], but null when comparing lower levels of homework (i.e., medium homework levels, 90–120 min/day, and 61–90 min/day) [89]

^j^ 8/11 studies had high risk of performance bias, with no evidence of psychometric testing for subjective exposure measures

^k^ 1: favourable overall, but null relationships were seen for boys weekdays and girls weekend days [92]

^l^ 2: Favourable & null [1: Favourable when doing homework without computer, null when doing homework with computer [95], 1: Favourable for homework, null for cram school attendance [96]]; 1: Favourable & unfavourable [1: Favourable when looking at student time on homework, unfavourable when looking at mean school time on homework [97]]

Among the clustered RCT study design, findings were consistently reported as null in 4/7 studies [37, 43, 78, 79], and mixed in 1/7 studies [52]. Additionally, 2/7 studies (2 studies, 1 article) compared types of sedentary behaviours [77]. Of the 2 studies comparing types of sedentary behaviours, screen-based learning was compared to usual classroom learning [77]. Based on categories of school-related sedentary exposures, consistent null associations were seen for active breaks [78], active lessons [79], and additional physical activity [37, 43]; while screen-based learning was favourable for cognitive indicators compared to the usual classroom condition [77]. Overall, the quality of evidence was rated as moderate due to serious risk of indirectness.

Among RCT study designs, no overall consistent null, favourable, or unfavourable directions of results were observed with 1/3 studies reporting mixed findings [80] and 2/3 studies comparing types of sedentary behaviours [81, 82] also with no clear direction in findings. Mixed findings were explained in one study by dose of physical activity, as null associations were observed when comparing no active breaks to one active break, while more sedentary behaviour was unfavourable for cognitive indicators when comparing no active breaks to two active breaks [80]. Overall, the quality of evidence was rated as high due to no serious risks to quality of evidence.

For cross-over study designs, screen time was the only school-related sedentary exposure and comparing types of sedentary behaviours were the only extracted results [83, 84]. A consistent direction of results was seen in 1/2 articles, as the paper-based condition was favourable for cognitive indicators compared to the screen-based condition [83]. Specifically, when taking the same paper-based or tablet-based math quiz, children performed better in the paper-based condition across all sub-scales of the quiz [83]. Whereas, in the other study no consistent direction of findings were reported between paper and screen based comparisons [84]. Overall, the quality of evidence was rated as high due to no serious risks to quality of evidence observed.

Among non-randomised interventions, findings were consistently reported as null in 2/3 studies [85, 86], and 1/3 studies compared types of sedentary behaviours [87]. Based on categories of school-related sedentary exposures, consistent null associations were observed for standing desks [85, 86]. Overall, the quality of evidence was rated as very low due to serious risk of bias.

For longitudinal study designs, the only exposure observed was homework. More homework was consistently favourable for cognitive indicators in 1/3 studies [88]. The 2/3 studies with mixed findings included dose-response relationships with favourable associations (e.g., “high homework” levels), and null associations (e.g., “medium homework” levels) [40, 89]. Overall, the quality of evidence was rated as very low due to serious risk of bias.

Among cross-sectional study designs, more school-related sedentary behaviour was consistently null for cognitive indicators in 1/11 studies [90], unfavourable for cognitive indicators in 1/11 studies [91], favourable for cognitive indicators in 6/11 studies [62, 63, 68, 92–94], and mixed for 3/11 studies [95–97]. Based on categories of school-related sedentary exposures, consistent null findings were observed for recess/PE [90] and sedentary time [68]. As well, 5/9 studies reported more homework was favourable for cognitive indicators [62, 63, 92–94], while 1/9 studies reported more homework was unfavourable for cognitive indicators [91]. Overall, the quality of evidence was rated as very low due to a serious risk of bias.

##### Musculoskeletal growth

A total of 3 studies examined the association between school-related sedentary behaviours and musculoskeletal growth (See Additional File 2, Supplementary Table 4 for the individual studies). Study designs included clustered RCT (*n* = 1), non-randomised intervention (*n* = 1), and cross-sectional (*n* = 1). For a summary of the measured musculoskeletal growth see Table 4.

**Table 4:** Summary of results for musculoskeletal growth indicators organized by study design

No. of participants (No. of studies)	Design	Risk of bias	Inconsistency	Indirectness	Imprecision	Other	Absolute effect	Quality
236 (1) [51]	Clustered RCT	Serious risk of bias^a^	No serious risk of inconsistency	No serious risk of indirectness	Serious risk of imprecision^b^	None	**Recess/PE:** • **1/1** study reported **null** findings [51]	Low
228 (1) [53]	Non-Randomised Intervention	Serious risk of bias^c^	No serious risk of inconsistency	Very serious risk of indirectness^d^	Serious risk of imprecision^e^	None	**Recess/PE:** • **1/1** studies reported **null** findings [53]	Very Low
1586 (1) [69]	Cross-sectional	Serious risk of bias^f^	No serious risk of inconsistency	No serious risk of indirectness	Serious risk of imprecision^g^	None	**Homework:** • **1/1** studies reported **mixed** findings • **1:** Null for boys and more homework favourable for girls health [69]	Very Low

Mean age at baseline ranged from 7.7 years to 11.5 years. Study designs included clustered RCT and non-randomised intervention with up to 7 years follow-up, and cross-sectional. Musculoskeletal growth was assessed objectively with height, weight, and fat-free mass (i.e., bioelectrical impedance, and skinfold thickness)

^a^ Groups were randomised to intervention and control within the same schools

^b^ Only one study, but not a small sample size

^c^ Intervention effects not reported, only differences in mean follow-up values

^d^ Study did not report school-related sedentary exposure

^e^ Only one study, but not a small sample size

^f^ Study did not demonstrate psychometric testing for subjective exposure measure

^g^ Only one study, but large sample size

For the clustered RCT and non-randomised intervention, the only exposure observed was recess/PE. Consistent null associations were observed in both studies [51, 53]. Overall, the quality of evidence was rated as low for the clustered RCT due to serious risk of bias and serious risk of imprecision, and very low for the non-randomised intervention due to serious risk of bias, very serious risk of indirectness, and serious risk of imprecision.

For the cross-sectional study, the only exposure observed was homework. Findings were mixed, with null results observed for boys and more homework favourable for musculoskeletal growth in girls [69]. Specifically, homework was positively associated with fat-free mass index in girls, but no significant associations were detected for boys. Overall, the quality of evidence was rated as very low for serious risk of bias and serious risk of imprecision.

##### Risks (injury)/harm

A total of 19 studies examined the association between school-related sedentary behaviours and risks (injury)/harm (See Additional File 2, Supplementary Table 5 for the individual studies). Study designs included a cross-over trial (*n* = 1), non-randomised interventions (*n* = 3), longitudinal (*n* = 1), and cross-sectional (*n* = 14). Additionally, one of these articles included longitudinal and cross-sectional study designs. For a summary of the measured outcomes, see Table 5.

**Table 5:** Summary of results for risks (injury)/harm indicators organized by study design

No. of participants (No. of studies)	Design	Risk of bias	Inconsistency	Indirectness	Imprecision	Other	Absolute effect	Quality
47 (1) [98]	Cross-Over Trial	Very serious risk of bias^a^	No serious risk of inconsistency	No serious risk of indirectness	Very serious risk of imprecision^b^	None	**Standing desk**: • **1/1** studies reported **mixed** findings^c^ • **1**: More sedentary behaviour null and unfavourable for health [98]	Very Low
178 (3) [54, 55, 85]	Non-Randomised Intervention	Very serious risk of bias^d^	No serious risk of inconsistency	No serious risk of indirectness	No serious risk of imprecision	None	**Standing desk**: • **3/3** studies reported **null** findings [54, 55, 85]	Very Low
1958 (1) [99]	Longitudinal	Serious risk of bias^e^	No serious risk of inconsistency	No serious risk of indirectness	Serious risk of imprecision^f^	Dose Response	**Homework**: • **1/1** studies reported **mixed** findings • **1:** Mixed findings included **dose response** relationships with **unfavourable** associations for ≥2 h of cram school attendance, and **null** associations for 0.5–1.9 h/day [99].	Very Low
472,293 (14) [63, 94, 99–110]	Cross-sectional	Serious risk of bias^g^	No serious risk of inconsistency	No serious risk of indirectness	No serious risk of imprecision	Dose Response	**Overall:** • **2/14** studies reported **null** findings, • **5/14** studies reported more sedentary behaviour **unfavourable** for health • **7/14** studies reported **mixed** findings • **5:** More sedentary behaviour null and unfavourable for health • **2:** More sedentary behaviour favourable, null, and unfavourable for health • **5:** Mixed findings included **dose response** relationships **Homework:** • **2/12** studies reported **null** findings [63, 100] • **3/12** studies reported more sedentary behaviour **unfavourable** for health [94, 101, 102] • **7/12** studies reported mixed findings^h^ • **5**: More sedentary behaviour null and unfavourable for health [99, 105–108] • **2**: More sedentary behaviour favourable, null, and unfavourable for health [109, 110] • **5**: Mixed findings included **dose response** relationships with **unfavourable** results for “too much”, ≥60 min/day, ≥ 2 h/day, 2–3 h/day, and > 3 h/day; **null** results for “not enough”, 1–30 min/day, 31–60 min/day, and 0.5–1.9 h/day homework, as well as 4–6 h/day and > 10 h/day of studying/sitting; and **favourable** results for 6–8 and 8–10 h of studying/sitting [99, 105–107, 110] **Recess/PE:** • **1/1** studies reported more sedentary behaviour **unfavourable** for health [103] **Screen time:** • **1/1** studies reported more sedentary behaviour **unfavourable** for health [104]	Very Low

Mean age at baseline ranged from 9.7 to 16.5 years; when mean age was not reported age or grade range minimums were 6.0 years and grade 1 and range maximums were 19.0 years and grade 12. Study designs included cross-over trial, non-randomized interventions, and longitudinal with up to 4 years follow-up, and cross-sectional. Risks (injury)/harms were assessed objectively through eye examinations for myopia, visual acuity, and visual impairment; and subjectively pain/discomfort (abdominal, ankles/feet, back, elbow, hip/thigh, knee, lower back, lower limbs, neck and shoulder, neck, shoulder, upper back, upper limbs, wrist/hands, and overall pain or discomfort using the Nordic Musculoskeletal Questionnaire, HBSC survey, or study-specific questionnaires), headaches (study-specific questionnaires), well-being (self-report HBSC), and global health (parent-report questionnaire)

^a^ Intervention first condition, and control group first condition were in the same classroom

^b^ Only one study, with a small sample size

^c^ Unfavourable for odds of elbow, low back, neck, and shoulder pain, as well as for less neck pain, but null for all other areas of pain [98]

^d^ 2/3 studies had intervention and control groups in the same school

^e^ Study did not demonstrate psychometric testing of subjectively measured exposure variables

^f^ Only one study, but did have large sample size

^g^ 9/15 studies did not demonstrate psychometric testing of subjectively measured exposure variables

^h^ 5: null and unfavourable [1: unfavourable for too much homework and shoulder pain, but null for not enough and shoulder pain, and all neck pain [105]; 1: unfavourable for too much, null for not enough [106]; 1: unfavourable for > = 60 min and myopia, null for all visual acuity and myopia 1–30 and 31–60 min homework [107]; 1: unfavourable overall and > = 2 h of homework, null for 0.5–1.9 h/day [99]; 1: unfavourable for boys weekday homework and odds of pain, but null for all other outcomes and sub-groups (11/12 associations) [108]; **2**: favourable and null and unfavourable findings [1: dose response-generally favourable associations at 1–2 h of studying, unfavourable at > 3 h and 2–3 h [109]; 1: dose response-favourable associations at 6–8 and 8–10 h, but null for > 10 h and 4–6 h (compared to < 4 h), and unfavourable for extra learning tasks after class [110]]

The cross-over trial exposure was standing desks. Findings were mixed, with more sedentary behaviour null and unfavourable for risks/harms when comparing traditional classroom designs to standing desks [98]. Overall, the quality of evidence was rated as very low due to very serious risk of bias and very serious risk of imprecision.

For the non-randomised interventions, the only observed exposure was standing desks. Consistent null findings were observed for each of the included studies [54, 55, 85]. Overall, the quality of evidence was rated as very low due to very serious risk of bias.

The longitudinal study exposure was homework. Findings were mixed but included dose-response relationships with unfavourable associations for ≥2 h of cram school (tutoring centres) attendance, and null associations for 0.5–1.9 h/day [99]. Overall, the quality of evidence was rated as very low due to serious risk of bias and serious risk of imprecision.

For the cross-sectional studies, null findings were observed in 2/14 studies [63, 100], more sedentary behaviour was unfavourable for risks/harms in 5/14 studies [94, 101–104], and mixed in 7/14 studies [99, 105–110]. More sedentary behaviour was consistently unfavourable for risks/harms when compared to recess/PE (indicating recess/PE was beneficial for preventing risks/harms) [103]. More screen time was also consistently unfavourable for risks/harms [104]. More homework was consistently null in 2/12 studies [63, 100] and unfavourable for risks/harms in 3/12 studies [94, 101, 102]. Mixed findings for homework included dose-response relationships in 5/12 studies, with unfavourable results (e.g., “too much” homework associated with more shoulder pain), null results (e.g., no association between “not enough” homework and shoulder pain), and favourable results (e.g., 6–8 h/day of studying or sitting associated with less neck and shoulder pain [sample median: 8–10 h/day of studying or sitting]) [99, 105–107, 110]. Overall, the quality of evidence was rated as very low due to serious risk of bias.

##### Social-emotional indicators

Twenty-one studies examined the association between school-related sedentary behaviours and social-emotional indicators (See Additional File 2, Supplementary Table 6 for the individual studies). Study designs included clustered RCTs (*n* = 4), non-randomised intervention (*n* = 1), longitudinal (*n* = 4), and cross-sectional (*n* = 12). For a summary of the measured outcomes, see Table 6.

**Table 6:** Summary of results for social-emotional indicators organized by study design

No. of participants (No. of studies)	Design	Risk of bias	Inconsistency	Indirectness	Imprecision	Other	Absolute effect	Quality
6095 (4) [49, 111–113]	Clustered RCT	No serious risk of bias	No serious risk of inconsistency	Very serious risk of indirectness^a^	No serious risk of imprecision	None	**Overall:** • **1/4** studies reported **null** findings • **2/4** studies reported **unfavourable** findings • **1/4** studies reported **mixed** findings • **1:** null and unfavourable • **1/4** studies also compared **types** of sedentary behaviours **Active Lessons:** • **2/4** studies reported **unfavourable** findings [111, 112] • **1/4** studies reported **mixed** findings^b^ • **1:** Null and unfavourable [113] • **1/4** studies compared **types** of sedentary • **1:** Sedentary game was favourable compared to sedentary lesson [113] **Additional PA:** • **1/1** studies reported **null** findings [49]	Low
49 (1) [85]	Non-Randomised Intervention	No serious risk of bias	No serious risk of inconsistency	No serious risk of indirectness	Very serious risk of imprecision^c^	None	**Standing Desks:** • **1/1** study reported **favourable** findings [85]	Very Low
4656 (4) [57, 114–116]	Longitudinal	Serious risk of bias^d^	No serious risk of inconsistency	No serious risk of indirectness	No serious risk of imprecision	None	**Overall:** • **1/4** studies reported **null** findings • **3/4** studies reported **mixed** findings • **2:** Null and unfavourable • **1:** Null and favourable **Active Breaks:** • **1/1** studies reported **mixed** findings^e^ • **1:** Null and unfavourable [115] **Homework:** • **1/3** studies reported **null** findings [114] • **2/3** studies reported **mixed** findings^f^ • **1:** Null and unfavourable [57] • **1:** Null and favourable [116] • **1:** Mixed findings included **dose response** relationships with **favourable** associations for up to 2 h, but **null** associations for ≥2 h [116]	Very Low
83,252 (12) [39, 63, 65, 68, 71, 117–123]	Cross-sectional	Serious risk of bias^g^	No serious risk of inconsistency	No serious risk of indirectness	No serious risk of imprecision	None	**Overall:** • **2/12** studies reported **null** findings • **1/12** studies reported **favourable** findings • **1/12** studies reported **unfavourable** findings • **8/12** studies reported mixed findings • **2:** Favourable and unfavourable • **2:** Null and unfavourable • **3:** Favourable and null • **1:** Favourable, null, and unfavourable **Homework:** • **1/10** studies reported **null** findings [117] • **1/10** studies reported **favourable** findings [118] • **1/10** studies reported **unfavourable** findings [119] • **7/10** studies reported **mixed** findings^h^ • **3:** Favourable and null [63, 65, 120] • **2:** Favourable and unfavourable [39, 121] • **1:** Null and unfavourable [122] • **1:** Favourable, null, and unfavourable findings [123] • **1:** Mixed findings included **dose response** relationships with **favourable** associations for 1–2 h/day and **null** associations for > 2 h/day [65] **Sedentary time:** • **1/2** studies reported **null** findings [71] • **1/2** studies reported **mixed** findings^i^ • **1:** Null and unfavourable [68]	Very Low

Mean age at baseline ranged from 8.8 to 17.0 years; when mean age was not reported age or grade range minimums were 10.0 years and grade 1 and range maximums were 15.0 years and grade 12. Study designs included clustered RCT, non-randomized interventions, and longitudinal with up to 3 years follow-up, and cross-sectional. Social-emotional indicators were assessed objectively for time on task (direct observation momentary time sampling) and subjectively for anxiety (Brief Symptom Inventory, and Generalised Anxiety Disorder 7-item Scale), body dissatisfaction (Eating Disorders Inventory-3), classroom amotivation (Classroom Behavior and Assets Scale), classroom attentiveness (Classroom Behavior and Assets Scale), classroom behavioural assets (Classroom Behavior and Assets Scale), classroom cheerfulness (Classroom Behavior and Assets Scale), classroom cooperation (Classroom Behavior and Assets Scale), classroom defiance (Classroom Behavior and Assets Scale), classroom effort (Classroom Behavior and Assets Scale), classroom inattention (Classroom Behavior and Assets Scale), classroom mood problems (Classroom Behavior and Assets Scale), classroom problematic behavior (Classroom Behavior and Assets Scale), classroom restlessness (Classroom Behavior and Assets Scale), conduct problems (Strengths and Difficulties Questionnaire), coping (Brief Resilient Coping Scale), covid-19 stress (Swine Flu Anxiety Scale), depression (Brief Symptom Inventory, Child Depression Inventory, and Center for Epidemiologic Studies Depression Scale Revised 10), depressive mood (Depressive Mood Scale), difficulties with classroom transitions (Classroom Behavior and Assets Scale), emotional problems (Strengths and Difficulties Questionnaire), flourishing (Flourishing Scale), health-related quality of life (Child Health Utility 9D-Chinese version, Kidscreen-10, PedsQL 4.0 Spanish version), hostility (Brief Symptom Inventory), hyperactivity (Strengths and Difficulties Questionnaire), loneliness (UCLA Loneliness Scale), negative self-esteem (Brief Symptom Inventory), peer problems (Strengths and Difficulties Questionnaire), persistence (School-Age Temperament Inventory), physical quality of life (Child Health Questionnaire), physical self-concept (Marsh’s Physical Self-Description Questionnaire), positive mental wellbeing (Warwick-Edinburgh Mental Well-being Scale), prosociality (Strengths and Difficulties Questionnaire), psychological distress (Malaise Inventory), psychological well-being (Flourishing Scale), psychological quality of life (Child Health Questionnaire), reactivity (School-Age Temperament Inventory), school subjective social status (Subjective Social Status Scale), social quality of life (Paediatric Quality of Life Inventory), society subjective social status (Subjective Social Status Scale), somatization (Brief Symptom Inventory), study and interpersonal stress (Student-life Stress Inventory), suicidal attempt (Kiddie Schedule for Affective Disorders and Schizophrenia), suicidal ideation (Kiddie Schedule for Affective Disorders and Schizophrenia), total difficulties (Strength and Difficulties questionnaire)

^a^ Only 1/4 studies reported intervention effect of decreasing school-related sedentary behaviours

^b^ 1: Null & unfavourable [Null when comparing the low/moderate physical activity game to the sedentary game conditions, but unfavourable when comparing sedentary lesson to low/moderate physical activity game, or MVPA game to either sedentary condition [113]]

^c^ Only one study, and small sample size

^d^ 3/4 studies used subjective exposure measures with no evidence of psychometric testing

^e^ 1: Null and unfavourable [1: Unfavourable for lack of effort or motivation, but null for all other outcomes (12/13 null associations) [115]]

^f^ 1: Null and unfavourable [1: unfavourable for change in homework, but null for baseline homework [57]]; 1: Null and favourable [favourable for psychological distress trend and up to 2 h (Dose response), but null for > = 2 h, and all other outcomes [116]]

^g^ 8/12 studies reported subjective exposures without evidence of psychometric testing

^h^ 3: Favourable and null [1: favourable for weekday homework in girls, but null for all other comparisons [120]; 1: Favourable for persistence and screen-based homework, but null for all other outcomes [63]; 1: Dose response-1-2 h favourable, > 2 h null [65]]; 2: Favourable and unfavourable [1: Favourable for loneliness and depression, unfavourable for COVID stress [121]; 1: Adding 15 min/day of homework unfavourable for anxiety, favourable for depression (except subtracting sleep unfavourable for those with < 8 h of sleep), and favourable for flourishing (except unfavourable when subtracting MVPA) [39]] 1: Null and unfavourable [1: Unfavourable for suicidal ideation, but null for suicide attempts [122]]; 1: favourable and null and unfavourable findings [1: Null for Asian-Australians, for Caucasian-Australians null for depressive mood, favourable for coping, and unfavourable for study and interpersonal stress [123]]

^i^ 1: Null and unfavourable [1: Null for society subjective social status, unfavourable for school subjective social status [68]]

Among clustered RCTs, a consistent direction in results was observed as null in 1/4 studies [49], more sedentary behaviour was unfavourable for social-emotional indicators in 2/4 studies [111, 112], and 1/4 studies reported a mix of null and unfavourable findings and compared types of sedentary behaviours [113]. For exposure categories, more sedentary behaviour was consistently null for additional physical activity [49]. More sedentary behaviour was consistently unfavourable for social-emotional indicators when compared to active lessons in 2/4 studies. One study found a sedentary game was favourable for social-emotional indicators when compared to a sedentary lesson [113]. Overall, the quality of evidence was rated as low due to very serious risk of indirectness.

For the non-randomised intervention, the only observed exposure was standing desks. More sedentary behaviour was consistently favourable for social-emotional indicators [85]. Specifically, the standing desk intervention group had higher total difficulties (i.e., hyperactivity, emotional symptoms, conduct problems, and peer problems) scores, compared to the traditional classroom control group. Overall, the quality of evidence was rated as very low very due to serious risk of imprecision.

Among longitudinal studies, findings were consistently observed as null in 1/4 studies [114] and mixed in 3/4 studies [57, 115, 116]. While no consistent findings were observed across exposure categories, 1/3 studies examining the exposure of homework reported consistent null findings [114]. Overall, the quality of evidence was rated as very low due to serious risk of bias.

For the cross-sectional studies, findings were consistently reported as null in 2/12 studies [71, 117], while more sedentary behaviour was favourable in 1/12 studies [118], unfavourable in 1/12 studies [119], and mixed findings were reported for 8/12 studies [39, 63, 65, 68, 120–123]. No consistent findings were reported across exposure categories. However, consistent findings in some studies were observed as null for homework [117], and sedentary time [71]. More homework was favourable for social-emotional indicators [118] and unfavourable for social-emotional indicators [119]. Overall, the quality of evidence was rated as very low due to serious risk of bias.

#### Important outcomes

##### Fitness

Thirteen studies examined the association between school-related sedentary behaviours and fitness (See Additional File 2, Supplementary Table 7 for the individual studies). Study designs included clustered RCTs (*n* = 6), non-randomised interventions (*n* = 2), and cross-sectional (*n* = 5). For a summary of the measured outcomes, see Table 7.

**Table 7:** Summary of results for fitness indicators organized by study design

No. of participants (No. of studies)	Design	Risk of bias	Inconsistency	Indirectness	Imprecision	Other	Absolute effect	Quality
4211 (6) [37, 49–51, 124, 125]	Clustered RCT	No serious risk of bias	No serious risk of inconsistency	Very serious risk of indirectness^a^	No serious risk of imprecision	None	**Overall:** • **3/6** studies reported **null** findings • **1/6** studies reported more sedentary behaviour **unfavourable** for health • **2/6** studies reported **mixed** findings • **1:** More sedentary behaviour favourable, null, and unfavourable for health • **1:** More sedentary behaviour null and unfavourable for health **Active breaks:** • **1/1** studies reported **mixed** findings^b^ • **1:** More sedentary behaviour null and unfavourable for health [124] **Additional PA:** • **2/4** studies reported **null** findings [37, 50] • **1/4** studies reported more sedentary behaviour **unfavourable** for health [49] • **1/4** studies reported **mixed** findings^c^ • **1:** More sedentary behaviour favourable, null, and unfavourable for health [125] **Recess/PE:** • **1/1** studies reported **null** findings [51]	Low
487 (2) [56, 126]	Non-Randomised Intervention	No serious risk of bias	No serious risk of inconsistency	Serious risk of indirectness^d^	No serious risk of imprecision	None	**Active breaks:** • **2/2** studies reported more sedentary behaviour **unfavourable** for health [56, 126]	Very Low
526,998^e^ (5) [60, 62, 71, 92, 127]	Cross-sectional	Serious risk of bias^f^	No serious risk of inconsistency	Serious risk of indirectness^e^	No serious risk of imprecision	None	**Overall:** • **4/5** studies reported **null** findings • **1/5** studies reported more sedentary behaviour **unfavourable** for health **Active breaks:** • **1/1** studies reported **null** findings [60] **Additional PA:** • **1/1** studies reported **null** findings [60] **Homework:** • **2/2** studies reported **null** findings [62, 92] **Recess/PE:** • **1/1** studies reported **null** findings [60] **Sedentary time:** • **1/2** studies reported **null** findings [127] • **1/2** studies reported more sedentary behaviour **unfavourable** for health [71]	Very Low

Mean age at baseline ranged from 8.4 to 14.9 years; when mean age was not reported age or grade range minimums were 7.0 years and grade 1 and range maximums were 13.0 years and grade 5. Study designs included clustered RCT and non-randomized intervention with up to 4 years follow-up, and cross-sectional. Fitness indicators were assessed objectively for aerobic fitness (20 m Shuttle run, Andersen test, FitnessGram Progressive Aerobic Cardiovascular Endurance Run (PACER), FitnessGram Test, and Resting HR), balance (four rotations while standing on upside down stool), composite physical fitness score (vital capacity, standing long jump, 50 m run, flexibility, sit-up [for girls] or pull-up [for boys], and 800 m [for girls] or 1000 m [for boys] run tests, were performed according to the Chinese National Student Physical Fitness Standard [CNSPFS] battery), coordination (ball bouncing, and running to cones aligned with a number the administrator yells out), locomotor gross motor skills (TGMD-3), max running duration (modified Bruce protocol for children, portable spirometry system), maximum power (modified Bruce protocol for children, portable spirometry system), motor quotient (Body Coordination Test: Balancing backwards, one-legged obstacle jumping, lateral jumping, and sideways movements), motor skill composite score (catching with one hand, throwing at a wall target, and shuttle run [10 × 5 m]), muscular endurance (sit-ups), muscular power (standing broad jump, and standing long jump), muscular strength (handgrip strength, push-up [from knees], push-up [regular]), object control gross motor skills (TGMD-3), relative power (modified Bruce protocol for children, portable spirometry system), total gross motor skills (TGMD-3), and VO2 peak (modified Bruce protocol for children, portable spirometry system)

^a^ 4/6 studies did not report a school-related sedentary exposure, 1/6 studies found no intervention effect on school-related sedentary behaviours, and 1/6 studies found an intervention effect for one of the intervention arms (educational PA decreased school-related sedentary, recreational PA increased school-related sedentary)

^b^ 1: Null and unfavourable [1: unfavourable for muscular power (standing long jump and push-ups), but null for coordination (ball bouncing, and running to cones aligned with a number the administrator yells out) and balance (four rotations while standing on upside down stool) outcomes [124]]

^c^ 1: Favourable, null, and unfavourable [Favourable for girls hand grip strength, and for both genders (pooled) when compared to the educational PA intervention, cardiorespiratory fitness for girls and pooled sample when compared to recreational PA intervention; unfavourable for boys and pooled for cardiorespiratory fitness and sit-ups when compared to the educational PA intervention, and standing broad jump for girls when compared to the educational PA intervention; Null for all other outcome (cardio, handgrip, sit-ups, broad jump), genders (boys, girls, pooled), and intervention group comparisons (educational or recreational PA interventions) [125]]

^d^ 1/2 studies found an intervention effect on school-related sedentary exposures, 1/2 studies did not report a school-related sedentary exposures

^e^ 1 study sampled 905 schools and approximated sample size based on teachers estimates (~ 524,600)

^f^ 2/5 studies appeared to use convenience sampling to recruit participants

Among the clustered RCTs, findings were consistently observed as null in 3/6 studies [37, 50, 51], more sedentary behaviour was unfavourable for 1/6 studies [49], and mixed findings were reported in 2/6 studies [124, 125]. Consistent null findings were observed for the exposure category recess/PE [51]. Null associations were observed in 2/4 studies examining additional physical activity. As well, more sedentary behaviour was unfavourable for fitness in 1/4 studies examining additional physical activity, indicating that incorporating more physical activity in the school day schedule was beneficial for fitness. Overall, the quality of evidence was rated as very low due to very serious risk of indirectness.

The non-randomised interventions exposure was active breaks. More sedentary behaviour was unfavourable for fitness when comparing traditional sedentary classrooms with classrooms adding active breaks to the class [56, 126]. Overall, the quality of evidence was rated as very low due to serious risk of indirectness.

Among the cross-sectional studies, findings were consistently observed as null in 4/5 studies [60, 62, 92, 127] and more sedentary behaviour was unfavourable for fitness in 1/5 studies [71]. Across the exposure categories, consistent null findings were observed for active breaks [60], additional physical activity [60], homework [62, 92], and recess/PE [60]. More sedentary time was unfavourable for fitness in 1/2 studies [71] and null in 1/2 studies [127]. Overall, the quality of evidence was rated as very low due to serious risk of bias and serious risk of indirectness.

##### Other movement behaviours

A total of 61 studies examined the association between school-related sedentary behaviours and other movement behaviours (See Additional File 2, Supplementary Table 8 for the individual studies). Study designs included clustered RCTs (*n* = 14), cross-over trials (*n* = 3), non-randomised interventions (*n* = 14), longitudinal (*n* = 5), and cross-sectional (*n* = 25). Of these articles, multiple study designs were observed for combinations of longitudinal and non-randomised intervention (*n* = 1), cross-sectional and non-randomised intervention (*n* = 1), and cross-sectional and longitudinal (*n* = 2). Further, one article included results from two non-randomised interventions [44]. For a summary of the measured outcomes see Table 8.

**Table 8:** Summary of results for other movement behaviour indicators organized by study design

No. of participants (No. of studies)	Design	Risk of bias	Inconsistency	Indirectness	Imprecision	Other	Absolute effect	Quality
6051 (14) [43, 49–52, 78, 79, 111, 125, 128–132]	Clustered RCT	No serious risk of bias	No serious risk of inconsistency	Very serious risk of indirectness^a^	No serious risk of imprecision	None	**Overall:** • **4/14** studies reported **null** findings • **2/14** studies reported more sedentary behaviour **unfavourable** for health • **8/14** studies reported **mixed** findings • **7:** More sedentary behaviour null and unfavourable for health • **1:** More sedentary behaviour favourable, null, and unfavourable for health **Active breaks:** • **1/1** study reported **mixed** findings^b^ • **1:** More sedentary behaviour null and unfavourable for health [78] **Active lessons:** • **1/3** studies reported more sedentary behaviour **unfavourable** for health [111] • **2/3** studies reported **mixed** findings^c^ • **1:** More sedentary behaviour favourable, null, and unfavourable for health [79] • **1:** More sedentary behaviour null and unfavourable for health [129] **Additional PA:** • **2/6** studies reported **null** findings [43, 49] • **1/6** studies reported more sedentary behaviour **unfavourable** for health [128] • **3/6** studies reported **mixed** findings^d^ • **3:** More sedentary behaviour null and unfavourable for health [50, 125, 130] **Recess/PE:** • **1/1** studies reported **null** findings [51] **Standing Desk:** • **1/3** studies reported **null** findings [52, 132] • **2/3** studies reported **mixed** findings^e^ • **1:** More sedentary behaviour null and unfavourable for health [131]	Low
336 (3) [98, 133, 134]	Cross-Over Trial	Very serious risk of bias^f^	No serious risk of inconsistency	No serious risk of indirectness	No serious risk of imprecision	None	**Overall:** • **1/3** studies reported more sedentary behaviour **unfavourable** for health • **2/3** studies reported **mixed** findings • **2:** More sedentary behaviour null and unfavourable for health **Active Lessons:** • **1/1** study reported **mixed** findings^g^ • **1:** More sedentary behaviour null and unfavourable for health [134] **Standing Desks:** • **1/2** studies reported more sedentary behaviour **unfavourable** for health [133] • **1/2** studies reported **mixed** findings^h^ • **1:** More sedentary behaviour null and unfavourable for health [98]	Low
4392 (14) [44, 53–56, 85, 86, 115, 135–139]	Non-Randomised Intervention	Serious risk of bias^i^	No serious risk of inconsistency	No serious risk of indirectness	Serious risk of imprecision^j^	None	**Overall:** • **1/14** studies reported **null** findings • **4/14** studies reported more sedentary behaviour **unfavourable** for health • **9/14** studies reported **mixed** findings • **6:** More sedentary behaviour null and unfavourable for health • **3:** More sedentary behaviour favourable, null, and unfavourable for health **Active breaks:** • **2/3** studies reported more sedentary behaviour **unfavourable** for health [56, 115] • **1/3** studies reported **mixed** findings^k^ • **1:** More sedentary behaviour null and unfavourable for health [136] **Active Lessons:** • **1/1** studies reported **mixed** findings^l^ • **1:** More sedentary behaviour null and unfavourable for health [137] **Recess/PE:** • **2/2** studies reported **mixed** findings^m^ • **2:** More sedentary behaviour favourable, null, and unfavourable for health [53, 138] **Standing desk:** • **1/8** studies reported **null** findings [44, 54, 86, 139] • **2/8** studies reported more sedentary behaviour **unfavourable** for health [55, 135] • **5/8** studies reported **mixed** findings^n^ • **4:** More sedentary behaviour null and unfavourable for health [44, 54, 86, 139] • **1:** More sedentary behaviour favourable, null, and unfavourable for health [85]	Very Low
35,835 (5) [42, 57, 115, 140, 141]	Longitudinal	Serious risk of bias^o^	No serious risk of inconsistency	No serious risk of indirectness	No serious risk of imprecision	None	**Overall:** • **1/5** studies reported **null** findings • **1/5** studies reported more sedentary behaviour **unfavourable** for health • **3/5** studies reported **mixed** findings • **3:** More sedentary behaviour null and unfavourable for health **Active breaks:** • **1/1** studies reported **mixed** findings^p^ • **1:** More sedentary behaviour null and unfavourable for health [115] **Homework:** • **1/3** studies reported more sedentary behaviour **unfavourable** for health [42] • **2/3** studies reported **mixed** findings^q^ • **2:** More sedentary behaviour null and unfavourable for health [57, 141] **Recess/PE:** • **1/2** studies reported **null** findings [140] • **1/2** studies reported **mixed** findings^r^ • **1:** More sedentary behaviour null and unfavourable for health [115]	Very Low
299,148 (25) [42, 45, 62, 71, 74, 75, 91, 93, 94, 108, 121, 123, 137, 140, 142–152]	Cross-sectional	Serious risk of bias^s^	No serious risk of inconsistency	No serious risk of indirectness	No serious risk of imprecision	None	**Overall:** • **3/25** studies reported **null** findings • **5/25** studies reported more sedentary behaviour **unfavourable** for health • **2/25** studies reported more sedentary behaviour **favourable** for health • **15/25** studies reported **mixed** findings • **10:** More sedentary behaviour null and unfavourable for health • **1:** More sedentary behaviour favourable, null and unfavourable for health • **2:** More sedentary behaviour favourable and unfavourable for health • **2:** More sedentary behaviour favourable and null for health • **4:** Mixed findings included **dose response** relationships **Homework:** • **2/18** studies reported **null** findings [123, 142] • **2/18** studies reported more sedentary behaviour **unfavourable** for health [42, 143] • **2/18** studies reported more sedentary behaviour **favourable** for health [121, 146] • **12/18** studies reported **mixed** findings^t^ • **7:** More sedentary behaviour null and unfavourable for health [62, 74, 91, 147–150] • **2:** More sedentary behaviour null and favourable for health [93, 151] • **2:** More sedentary behaviour favourable and unfavourable for health [94, 152] • **1:** More sedentary behaviour favourable, null, and unfavourable for health [108] • **3:** Mixed findings included **dose response** relationships with **unfavourable** associations for ≥3 h/day and 2–3 h/day, **favourable** for 1–3 h/day, and null for 1–2 h/day and 2–3 h/day [149, 150, 152] **Recess/PE:** • **1/4** studies reported **null** findings [140] • **1/4** studies reported more sedentary behaviour **unfavourable** for health [144] • **2/4** studies reported **mixed** findings^u^ • **2:** More sedentary behaviour null and unfavourable for health [75, 137] **Sedentary time:** • **2/3** studies reported more sedentary behaviour **unfavourable** for health [71, 145] • **1/3** studies reported **mixed** findings^v^ • **1:** More sedentary behaviour null and unfavourable for health [45] • **1:** Mixed findings included **dose response** relationships with **unfavourable** for all comparisons between not sedentary and most sedentary, **null** for comparisons with less and more sedentary to most sedentary [45]	Very Low

Mean age at baseline ranged from 6.0 to 17.0 years; when mean age was not reported age or grade range minimums were 9.0 years and grade 1 and range maximums were 19.0 years and grade 12. Study designs included clustered RCT, cross-over trial, non-randomized intervention, and longitudinal with up to 7 years follow-up, and cross-sectional. Other movement behaviour indicators were assessed objectively for physical activity during and outside of school (heart rate 50–59.9% of max, heart rate ≥ 60% of max, light-intensity physical activity, metabolic equivalent(MET)-minutes, METs < 3, METs ≥3, moderate-intensity physical activity, moderate- to vigorous-intensity physical activity, physical activity guideline adherence, sit-to-stand transitions, standing time, stepping time, steps/day guideline adherence, total steps, total accelerometer x counts during field trip, total accelerometer y counts during field trip, total accelerometer counts per minute, total physical activity, and vigorous-intensity physical activity, using ActiGraph GT1M/GT3X/GT3X+/WGT3X-BT/GT9X Link accelerometers, ActivPAL/3/3C/micro accelerometers, Axivity AX3 accelerometers, Sensewear accelerometers, or Yamax Digi-Walker SW-200 pedometers), sedentary behaviours outside of school (sedentary time and sitting time, using ActiGraph GT3X and ActivPAL micro accelerometers), and sleep (sleep duration, using ActivPAL micro visual inspection and logsheets); or subjectively for physical activity during and outside of school (active videogames, activity usually, activity yesterday, days meeting physical activity guideline adherence, exercise habits, Godin Leisure-Time Exercise Questionnaire, leisure activity index, organized leisure physical activity, physical activity guideline adherence, Physical Activity Questionnaire-Children (PAQ-C), physical exercise index, total physical activity), sedentary behaviour outside of school (computer for communicating, computer for playing games, electronic videogames, mobile for communicationg, mobile for playing games, overall sedentary screen media usage, overall screen time, passive videogames, personal computer (PC) use, recreational screen time, screen based social networking, sedentary screen time, talking on the phone, television, texting, and video chatting), and sleep outside of school (bedroom-sharing, bedtime routine, daytime tiredness, daytime sleep, daytime sleepiness, difficulty initiating sleep, difficulty maintaining sleep, insomnia, school day bedtime, sleep duration, sleep guideline adherence, sleep hygiene [cognitive, emotional, physiological, and overall], sleep stability, and wake time)

^a^ 4/14 studies did not find an intervention effect on sedentary behaviours measures, and 6/14 studies did not report school-related sedentary behaviours

^b^ Null for sedentary vs light activity conditions, but unfavourable for sedentary vs moderate or vigorous conditions [78]

^c^ 1: Favourable and null and unfavourable findings [Unfavourable during the intervention, null for whole day (intervention and post-intervention periods), and after the intervention null, unfavourable, and favourable for LPA, MPA, and VPA, respectively [79]]; 1: null and unfavourable findings [1: Unfavourable for objectively assessed MPA and MVPA, null for VPA, LPA, and self-report PA [129]])

^d^ 3: Null and unfavourable [1: Overall unfavourable when compared to educational PA intervention group, null when compared to recreational PA intervention group [125]; 1: LPA group did not significantly differ, typical day unfavourable compared to MVPA group, restricted PA unfavourable compared to typical day, null effects for post-intervention compensation [130]; 1: Unfavourable for CPM, MVPA and Steps, but null for LPA; for boys null for CPM and LPA, unfavourable for MVPA and steps; for girls unfavourable for CPM, null for LPA, MVPA, and Steps [50]]

^e^ 2: Null and unfavourable [1: Unfavourable school-time MVPA, standing time and weekday standing time, null for all other context (outside school, school time, whole day weekends, whole day weekdays) and outcome (LPA, MVPA, standing, sitting, steps, sit-to-stand transitions, sleep) comparisons [131]; 1: Null associations for screen time, stepping time in secondary and primary school children, and standing time in secondary school children, but sedentary behaviour unfavourable for standing time in primary school children [132]]

^f^ No study randomized conditions

^g^ 1: null & unfavourable [1: unfavourable for all outcomes (MET-minutes, LPA, MPA, and VPA) at school A, B, and D, but school C null for LPA and VPA [134]

^h^ 1: Null & unfavourable [1: unfavourable for school day standing time, but null for full day sedentary time, LPA, MPA, and VPA [98]]

^i^ 7/14 studies had control group in the same school/class, 8/14 studies insufficiently reported outcome results (e.g., only post values)

^j^ 4/14 studies did not report a school-related sedentary measure, 3/14 studies did not demonstrate an intervention effect

^k^ 1: Null and unfavourable [1: overall null for MVPA and TPA, unfavourable for LPA, all null for just overweight participants, unfavourable for LPA and TPA in normal weight participants, but null for MVPA [136]]

^l^ 1: Null and unfavourable [1: overall unfavourable when comparing intervention time to class time for LPA and MVPA, and when comparing intervention day to non-intervention day for LPA, but null when comparing intervention day to non-intervention day for MVPA [137]]

^m^ 2: Favourable, null, and unfavourable findings [1: favourable only occurred for leisure time outcomes, unfavourable only occurred for overall school time outcomes, null occurred across all domains (total, weekend, school-time, leisure time, PE, and recess) [138]; 1: Overall unfavourable for physical activity measure, but null for screen time; favourable for girls screen time, but null for girls PA; unfavourable for boys PA, but null for boys screen time [53]]; **1:** Null and unfavourable [1: null for LPA when comparing PE days, unfavourable for all other combinations [137]]

^n^ 5: null and unfavourable [2: some unfavourable findings for steps, but overall null (1 study, two experiments) [44]; 1: unfavourable for school time sit-to-stand transitions, but null for all other movement behaviours in class, in school and during waking hours [54]; 1: unfavourable for MVPA, but null for LPA [139]; 1: unfavourable for standing time, but null for steps [86]]; 1: Favourable, null, and unfavourable [1: favourable for after school stepping time, unfavourable for class time standing and stepping, but null for after school standing, and class time standing and stepping [85]])

^o^ Subjective measures of outcomes in 4/5 studies and exposures in 5/5 studies, with no evidence of psychometric testing

^p^ 1: Null and unfavourable [1: unfavourable for “ever” held active breaks and time spent in active breaks, but null for held active breaks in the past week and breaks > = 3 min/day [115]]

^q^ 2: Null and unfavourable [1: Null for changes in homework from time 1 to time 2, but unfavourable for time 1 homework [57]; 1: Unfavourable for senior high, but null for junior high [141]]

^r^ 1: Null and unfavourable [1: null for recess, but unfavourable for PE [115]]

^s^ 19/25 studies used subjective exposure measure with no psychometric testing

^t^ **7:** null and unfavourable [1: Unfavourable for cram school attendance and weekend sleep duration, and weekday homework duration and weekday sleep, but null for all other weekday and weekend sleep and homework combinations and cram school attendance [147]; 1: Unfavourable for screen time, null for exercise habits [62]; 1: Unfavourable for some sleep quality aspects in older children, but null for all aspects in younger children [148]; 1: Dose response: More null associations for: weekdays (13/22 null associations) and 1–2 h of homework comparisons (8/10 null associations); more unfavourable associations for ≥3 and 2–3 h (both 7/10 unfavourable associations), and weekends (21/30 unfavourable associations) [149]; 1: Unfavourable screen time for girls and girls stressed about homework, and MVPA for boys and boys stressed about homework; Null for sleep, girls MVPA, boys not stressed about homework MVPA, boys screen time, and girls not stressed about homework screen time [74]; 1: Unfavourable for sleep, computer, overall sedentary screen time, and various other screen time; Null for passive and active videogames [91]; Dose response: generally unfavourable for aspects of sleep duration and quality at > 3 h of homework, null for 1–2 and 2–3 h of homework [150]]; **2:** Null and favourable [1: Favourable for video games, talking on the phone, TV on weekdays; Null for TV on weekends, texting, video chatting [93]; 1: Favourable for homework on school nights, null for homework before school [151]]; **2:** Favourable and unfavourable [1: Dose response: favourable for 1–3 h, unfavourable for > 3 h [152]; 1: Unfavourable for sleep and overall homework; Favourable for video games, talking on the phone, TV on weekdays, screen time on weekdays, sleep and weekday homework [94]; **1:** Favourable, null, and unfavourable [1: Unfavourable for PC for boys, TV and weekday homework for boys, TV and weekday homework for girls, PC and weekday homework for girls, PC on weekend and homework on weekends for girls; favourable for TV and weekend homework for girls; null for all physical activity and homework combinations [108]]

^u^ 2: Null and unfavourable [1: Unfavourable only for LPA and MVPA during school split for boys and girls, but null for all other time, weight class, gender, and outcome comparisons (36 null comparisons) [75]; 1: Null when comparing days with and without PE, unfavourable for school-related sedentary behaviours for all other comparisons [137]]

^v^ 1: Null and unfavourable [Dose response: For fully adjusted analyses unfavourable for all comparisons between not sedentary and most sedentary, null for comparisons with less and more sedentary to most sedentary [45]])

Among the clustered RCTs, findings were consistently observed as null in 4/14 studies [43, 49, 51, 52], more sedentary behaviour was unfavourable for other movement behaviours in 2/14 studies [111, 128], and mixed findings were reported in 8/14 studies [50, 78, 79, 125, 129–132]. Null findings were observed for recess/PE [51]. Consistent null findings were observed in 2/6 studies examining additional physical activity [43, 49], and 1/3 studies examining standing desks [52]. Further, more sedentary behaviour was consistently unfavourable for other movement behaviours in 1/3 studies comparing traditional lessons with active lessons [111], and 1/7 studies comparing traditional school days with school days incorporating various forms of additional physical activity [128]. Overall, the quality of evidence was rated as low due to very serious risk of indirectness.

For the cross-over trials, more school-related sedentary behaviour was consistently unfavourable for other movement behaviours in 1/3 studies [133] and mixed findings were reported for 2/3 studies [98, 134]. No exposure category contained only null, favourable, or unfavourable directions of results. More sedentary behaviours were consistently unfavourable for other movement behaviours in 1/2 studies comparing usual sedentary conditions to standing desks [133]. Overall, the quality of evidence was rated as low due to very serious risk of bias.

Among non-randomised interventions, associations between school-related sedentary behaviour and other movement behaviours were consistently null in 1/14 studies [54], more sedentary behaviour was unfavourable for other movement behaviours in 4/14 studies [55, 56, 115, 135], and mixed findings were reported in 9/14 studies [44, 53, 54, 85, 86, 136–139]. No consistent directions of results were seen across exposure categories. More sedentary behaviours were consistently unfavourable for other movement behaviours in 2/3 studies comparing a typical school day with school days incorporating active breaks [56, 115] and 2/8 studies comparing traditional classrooms to those with standing desks [55, 135]. Overall, the quality of evidence was rated as very low due to serious risk of bias and serious risk of indirectness.

For longitudinal studies, associations between school-related sedentary behaviour and other movement behaviours were observed as null in 1/5 studies [140], more sedentary behaviour was unfavourable for other movement behaviours in 1/5 studies [42], and mixed findings were reported in 3/5 studies [57, 115, 141]. No consistent trends were seen across exposure categories. More sedentary behaviour was consistently unfavourable for other movement behaviours in 1/3 studies examining homework [42], and null in 1/2 studies examining recess/PE [140]. Overall, the quality of evidence was rated as very low due to serious risk of bias.

Among cross-sectional studies, associations between school-related sedentary behaviour and other movement behaviours findings were consistently null for 3/25 studies [123, 140, 142], while more sedentary behaviour was unfavourable for other movement behaviours in 5/25 studies [42, 71, 143–145], favourable for other movement behaviours in 2/25 studies [121, 146], and mixed for 15/25 studies [45, 62, 74, 75, 91, 93, 94, 108, 137, 147–152]. Across exposure categories, no consistent directions of results towards null, favourable, and unfavourable were seen. Consistent null findings were observed in 2/18 studies examining homework [123, 142], and 1/4 studies examining recess/PE [140]. More sedentary behaviour was consistently favourable for other movement behaviours in 2/18 studies examining homework [121, 146], and consistently unfavourable for other movement behaviours in 2/18 studies examining homework [42, 143], 1/4 studies examining recess/PE [144], and 2/3 studies examining sedentary time [71, 145]. Overall, the quality of evidence was rated as very low due to serious risk of bias.

### High level summary of results

To facilitate the interpretation of the findings in this review, high-level summaries of results ungrouped from study designs, were completed for each extracted result that was classified as null, favourable, or unfavourable. For the high-level summary by outcome category, most results were null (See Table 9). However, more sedentary behaviour was favourably associated with approximately one-third of extracted associations for cognitive (33%) and social-emotional (32%) indicators. As well, more school-related sedentary behaviour was unfavourably associated with around one-third of extracted associations for other movement behaviours (35%). Ranges of quality of evidence are only presented in the high-level outcome category summary, since quality of evidence was rated by outcome categories and study design, but not for the high-level exposure category summary. Table 10 shows a high-level summary by exposure type, where null results were most frequently observed. However, more school-related sedentary behaviour was unfavourably associated with any health and well-being indicator when compared to active lessons in 72% of the extracted associations—indicating a benefit for active lessons in 72% of extracted associations. While 100% of results for screen time was also unfavourable, this only represented one extracted result, as most extracted associations for screen time compared types of sedentary behaviours.

**Table 9:** High-Level Summary of Results by Outcome Category

Outcome Category	Quality of Evidence	More SB Favourable for Health	Null	More SB Unfavourable for Health
**Critical Outcomes**				
*Adiposity Indicators*	Low to very low	2% (3)	77% (119)	21% (33)
*Biomarkers*	Moderate to very low	0% (0)	100% (15)	0% (0)
*Cognitive Indicators*	High to very low	33% (27)	57% (46)	10% (8)
*Musculoskeletal Growth*	Very low	14% (1)	86% (6)	0% (0)
*Risks (Injury)/Harms*	Very low	7% (8)	65% (70)	28% (30)
*Social-Emotional Indicators*	Low to very low	32% (32)	43% (43)	26% (26)
**Important Outcomes**				
*Fitness*	Very low	7% (5)	72% (49)	21% (14)
*Other Movement Behaviours*	Low to very low	4% (21)	61% (367)	35% (210)

Values represent the percent (frequency) of all extracted associations between a school-related sedentary behaviour exposure and health and well-being indicator, grouped by health and well-being indicator categories

*SB* Sedentary behaviour

**Table 10:** High-Level Summary of Results by Exposure Category

Exposure Category	More SB Favourable for Health	Null	More SB Unfavourable for Health
*Active Breaks*	0% (0)	62% (32)	38% (20)
*Active Lessons*	2% (1)	27% (16)	72% (43)
*Additional PA*	4% (5)	72% (94)	24% (32)
*Homework*	16% (75)	55% (258)	29% (136)
*Recess/PE*	5% (10)	75% (151)	20% (40)
*Screen Time*	0% (0)	0% (0)	100% (1)
*Standing Desk*	3% (4)	78% (105)	19% (26)
*Sedentary Time*	2% (2)	70% (59)	27% (23)

Values represent the percent (frequency) of all extracted associations between a school-related sedentary behaviour exposure and health and well-being indicators, grouped by school-related sedentary behaviour categories

*PA* Physical activity, *PE* Physical education, *SB* Sedentary behaviour

To further aide interpretation of the review findings, high-level summaries of results by outcome and exposure categories were also examined separately for primary (~ 5–12 years) and secondary (~ 13–18 years) school-aged children (See Table 11). For instance, more homework was favourable for any health and well-being indicator in 4% of extracted results for primary school children, and 25% of extracted results for secondary school children. Further, more sedentary behaviour was favourable for secondary school-aged children in nearly half of extracted associations for cognitive (48%) and social-emotional indicators (42%), compared to slightly over 10% for cognitive (14%) and for social-emotional (12%) indicators in primary school-aged children.

**Table 11:** High-Level Summaries by Outcome, Exposure, and Age-Group Categories

	More SB Favourable for Health		Null		More SB Unfavourable for Health	
Outcomes Categories	*Primary*	*Secondary*	*Primary*	*Secondary*	*Primary*	*Secondary*
**Critical Outcomes**						
*Adiposity*	2% (2)	0% (0)	77% (73)	77% (43)	21% (20)	23% (13)
*Biomarkers*	0% (0)	0% (0)	100% (15)	0% (0)	0% (0)	0% (0)
*Cognitive*	14% (5)	48% (19)	81% (30)	40% (16)	5% (2)	12% (5)
*MSK Growth*	14% (1)	0% (0)	86% (6)	0% (0)	0% (0)	0% (0)
*Risks*	0% (0)	11% (5)	73% (35)	66% (29)	27% (13)	23% (10)
*Social-emotional*	12% (3)	42% (28)	65% (17)	28% (19)	23% (6)	30% (20)
**Important Outcomes**						
*Fitness*	0% (0)	14% (5)	72% (23)	72% (26)	28% (9)	14% (5)
*Other movement behaviours*	2% (11)	5% (7)	64% (2815)	57% (79)	3334% (149)	38% (52)
**Exposure Categories**						
*Active breaks*	0% (0)	0% (0)	62% (32)	0% (0)	38% (20)	0% (0)
*Active lessons*	2% (1)	0% (0)	27% (16)	0% (0)	72% (43)	0% (0)
*Additional PA*	0% (0)	10% (5)	7877% (648)	62% (30)	232% (19)	27% (13)
*Homework*	4% (7)	25% (58)	62% (120)	51% (117)	34% (65)	24% (55)
*Recess/PE*	5% (9)	0% (0)	79% (139)	48% (10)	16% (28)	52% (11)
*Screen Time*	0% (0)	0% (0)	0% (0)	0% (0)	0% (0)	100% (1)
*Standing desk*	4% (4)	0% (0)	81% (92)	62% (13)	16% (18)	38% (8)
*Sedentary time*	4% (1)	2% (1)	71% (17)	70% (42)	25% (6)	28% (17)

Values represent the percent (frequency) of all extracted associations between a school-related sedentary behaviour exposure and health and well-being indicator, grouped by categories for school-related sedentary behaviours, health and well-being indicators, and age groups

*MSK* Musculoskeletal, *SB* Sedentary behaviour

Several instances of mixed directions of associations being explained by dose-response relationships between homework and health and well-being indicators were observed. Thus, a summary table was created to compile these associations, and explore the various dose-response relationships between homework and health and well-being indicators (See Table 12). A possible trend was seen for ≥2 h/day of homework being unfavourable for health and well-being. Trends in the null or favourable directions of associations were less apparent.

**Table 12:** Dose-response relationships explaining mixed results for the associations between homework and health and well-being indicators

Dose of Homework Unfavourable for Health	Null	Dose of Homework Favourable for Health
1–2 h/day (ref: < 1 h/day; 2/10 associations) [149]	> 0–0.5 h/day (ref: 0 h/day; 2/2 associations) [107]	> 0–2 h/day (ref: 0 h/day; 1/2 associations) [116]
> 1 h/day (ref: 0 h/day; 1/2 associations) [107]	> 0–2 h/day (ref: 0 h/day; 1/2 associations) [116]	1–2 h/day (ref: < 1 h/day; 1/1 association) [65]
2–3 h/day (ref: < 1 h/day; 14/18 associations) [149, 150]	0.5–1.9 h/day (ref: < 0.5 h/day; 2/2 associations) [99]	> 1–3 h/day (ref: 0–1 h/day; 1/1 association) [150]
≥2 h/day (ref: < 0.5 h; 2/2 associations) [99]	> 0.5–1 h/day (ref: 0 h/day; 2/2 associations) [107]	> ~ 2.5 h/day (ref: < 1 h/day; 1/2 associations) [40]^a^
> 3 h/day (ref: < 1 h/day and 0–1 h/day; 11/21 associations) [59, 149, 150, 152]	1–2 h/day (ref: < 1 h/day; 17/19 associations) [65, 149, 152]	6–8 h/day (ref: < 4 h; 3/4 associations) [110]^b^
“Too much homework” (ref: “just right amount of homework”; 3/4 associations) [105, 106]	1–3 h/day (ref: < 1 h/day; 2/2 associations) [59]	8–10 h/day (ref: < 4 h; 2/4 associations) [110]^b^
	> 1.0–1.5 h/day (ref: < 1 h/day; 2/2 associations) [40]^a^	“High homework” levels (ref: “low homework”; 1/1 association) [89]
	> 1 h/day (ref: 0 h/day; 1/2 associations) [107]	
	1.5- ~ 2.5 h/day (ref: < 1 h/day; 2/2 associations) [40]^a^	
	2–3 h/day (ref: < 1 h/day; 10/18 associations) [149, 152]	
	≥2 h/day (ref: 0 h/day; 4/4 associations) [65, 116]	
	> ~ 2.5 h/day (ref: < 1 h/day; 1/2 associations) [40]^a^	
	> 3 h/day studying (ref: < 1 h/day; 10/20 associations) [59, 149, 152]	
	4–6 h/day (ref: < 4 h; 4/4 associations) [110]^b^	
	6–8 h/day (ref: < 4 h; 1/4 associations) [110]^b^	
	8–10 h/day (ref: < 4 h; 2/4 associations) [110]^b^	
	> 10 h/day (ref: < 4 h; 4/4 associations) [110]^b^	
	“Not enough homework” (ref: “just right amount of homework”; 4/4 associations) [105, 106]	
	“Medium homework” levels (ref: “low homework”; 1/1 association) [89]	
	“Too much homework” (ref: “just right amount of homework”; 1/4 associations) [105, 106]	

*Ref* reference category

^a^ Study categorized homework time in quartiles, with durations changing at each time point. Quartile 1 (Q1): < 1.0 h/day for year 1–3 (Y1-Y3); Q2: > 1.0–1.5 h/day Y1-Y3; Q3: 1.5–2.0 (Y1)/2.5 (Y2)/3.0 (Y3) hours/day; Q4: > 2.0 (Y1)/2.5 (Y2)/3.0 (Y3) h/day

^b^ Time for all studies represent homework, studying, or cram school attendance except this study which measured hours/day spent studying or sitting (sample median: 8–10 h/day of studying or sitting)

## Discussion

### Summary of evidence

To help inform School-Related Sedentary Behaviour Recommendations, this systematic review examined the associations between school-related sedentary behaviours and inidcators of health and well-being in school-aged (~ 5–18 years) children. This was the first review to examine a comprehensive set of school-related sedentary behaviours and inidcators of health and well-being. Evidence was synthesized from 116 reports, including 1,385,038 participants and 1173 extracted associations. Based on high level summaries, the association between school-related sedentary behaviours and indicators of health and well-being were predominantly null. However, some evidence indicated more school-related sedentary behaviours could be favourable for cognitive and social-emotional indicators, and unfavourable for other movement behaviours. Further, when displacing school-related sedentary behaviours, active lessons were the most beneficial for students’ overall health and well-being. Compared to primary school-aged children, secondary school-aged children seemed to benefit from homework and had more favourable associations between school-related sedentary behaviours and cognitive and social-emotional indicators. Though high-level findings should be interpreted with some caution as findings are pooled across study designs and subsequent quality of evidence. The lower range of quality of evidence was very low for all health and well-being indicator categories, but upper ranges were observed as high for cognitive indicators.

Carson et al’s [2] review of sedentary behaviour and indicators of health and well-being in school-aged children found that homework was beneficial for cognitive indicators. Similarly, in the current review a favourable association between school-related sedentary behaviour and indicators of health and well-being was most frequently observed in the homework exposure category. These favourable associations seemed to be mainly for cognitive and social-emotional indicators. When examining dose-response relationships, higher levels of homework appeared to be unfavourable for health and well-being. Reverse causality could be an alternative explanation for the dose-response association between higher durations of time spent on homework and worse health and well-being, as children that spend more time on homework could be struggling to complete their assignments. Fernández-Alonso et al. [97] found homework duration was negatively associated with academic achievement at the individual level, but positively associated when looking at the amount the school assigns. While this could indicate there are benefits for schools to assign more homework, potential benefits should be interpreted with caution as further analyses revealed schools that assigned more homework widened the gap at the individual level for time spent on homework and academic achievement [97]. Thus, it could be said that assigning more homework at a school level adds inequity to students who struggle to complete homework based on cognitive or time constraints (e.g., after school employment). Further, placing an additional sedentary behaviour burden on children through homework and studying in pursuit of academic success could displace time in a 24-h day that could otherwise be spent on sleep and physical activity. This time displacement would ironically be counter-productive, based on the benefits to cognitive indicators from physical activity and adequate sleep for school-aged children [153, 154]. Regardless, homework demonstrated favourable associations with cognitive and social-emotional indicators, and is likely a valuable activity for children in moderation and at an age-appropriate level. For instance, in this review most favourable associations between homework and indicators of health and well-being were for secondary school-aged children. Thus, when determining the amount of homework assigned to children, teachers should consider how homework will enhance the academic development of all children, if homework is displacing time spent on other behaviours beneficial to academic development, and if the amount of homework is age-appropriate.

For school-related sedentary behaviour exposure categories, active lessons were overwhelmingly beneficial for health and well-being when displacing school-related sedentary behaviours. Active lessons are appealing since they simultaneously displace sedentary time and focus on educational pursuits. Further, within this review, evidence suggested that active lessons could improve children’s time on task behaviours or attention during class [111–113]. Additionally, no difference in content recall was observed when comparing content delivered through active lessons or traditional sedentary classroom conditions [79], suggesting that active lessons do not detract from learning objectives and could in fact enhance learning. Further, some studies not only incorporated active lessons into the school-day, but also conducted active lessons outside of the classroom. Considering the benefits of outdoor time for a range of health and well-being indicators [155–157], combining active lessons and outdoor time provides an additional opportunity to improve the health and well-being of school-aged children. Interestingly, most studies that examined active lessons were in the health and well-being indicator category of other movement behaviours (physical activity during active lessons), with 5 extracted associations for social-emotional indicators (time on task) and 1 extracted association for cognitive indicators (lesson content recall). Additionally, active lessons were only examined in primary school-aged children. Thus, future studies are needed to examine the benefits of active lessons across a range of health and well-being indicators, especially in outdoor settings where further benefits could be seen (e.g., myopia prevention [156]), and in secondary school-aged children.

Studies examining screen-based sedentary behaviours and indicators of health and well-being compared screen-based class time with other types of school-related sedentary behaviours (e.g., typical classroom time). Thus, it was difficult to make a broad claim that sedentary behaviour was favourable or unfavourable for health and well-being in high level summaries since sedentary behaviours were being compared to one another. Paper-based assessments (e.g., quizzes, writing accuracy) were favourable when compared to screen-based assessments [83, 84]. However, school-related screen time was beneficial when compared to non-educational screen time and traditional classroom learning, when lessons were built around screen time to serve a specific pedagogical purpose [77, 81, 82, 87]. Thus, it could be concluded that school-related screen time can be beneficial when it is meaningfully developed to serve a specific pedagogical purpose, and not implemented simply for the novelty of screens. Importantly, for several studies screen-based learning was seen as a means of meeting the United Nations Sustainable Development Goal of ensuring inclusive and equitable quality education for all [77, 81, 82]. Specifically, screen-based learning was examined to help overcome barriers specific to Malawi, where “school days are short, classrooms are overcrowded and poorly resourced, and teachers are frequently under qualified” [82]. While the merits of face-to-face learning versus screen-based learning can be debated, neither side can ignore the necessity of finding solutions for overcoming country specific barriers to delivering high-quality education for all children. Further, contingency plans are needed to prevent disrupted education if face-to-face learning in the classroom is not a possibility, as was seen in the COVID-19 crisis [158]. While COVID-19 could be seen as a global barrier to high-quality education, rural settings and low-middle income countries were disproportionately disrupted through a lack of infrastructure and equipment available to access online learning [158]. Thus, future research examining screen-based versus face-to-face learning should continue to reflect on how study results translate to inclusive and equitable education for all children internationally.

### Limitations

Several limitations of the included studies were observed. First, the quality of evidence was most frequently rated as very low. Future research should consider how study and evidence quality is evaluated [35, 36] when designing observational and experimental studies (e.g., exposure and outcome variables with sufficient psychometric evaluation, randomly sampling participants or schools), to aide the creation of high quality evidence. Second, most studies were cross-sectional. To better understand the causative mechanisms between school-related sedentary behaviours and indicators of health and well-being, more high-quality research is needed using longitudinal and experimental study designs. Third, few studies examined the health and well-being indicator categories of musculoskeletal growth (*n* = 3) and biomarkers (*n* = 4) compared to categories such as other movement behaviours (*n* = 62 studies). Fourth, 96% of studies were conducted in Europe, Asia, and North America with very few studies conducted in South America and Africa. Additionally, 71% of studies were conducted in high-income countries. More research is urgently needed to fill this gap, as review findings are limited in their ability to generalize to schools in Africa, South America, and low-middle income countries.

Further, several limitations existed specific to this review. First, the conceptualization of school-related sedentary behaviours included physical activity. Movement behaviours during the school day can be classified such that physical activity and sedentary behaviour (excluding screen time) are mutually exclusive and exhaustive, equating to perfectly collinear categories. Thus, any change to physical activity necessitates an equivalent change to sedentary behaviour, assuming no changes have been made to the length of the school day and sleep time is not part of the school day. Instead of assuming sedentary behaviours are displaced when adding physical activity, compositional analyses can examine this displacement. While two included studies used compositional analyses [61, 127], a future review should exclusively synthesize studies using compositional analyses to better understand the movement behaviour displacements occurring in the school setting. Further, a future review could examine compositional analyses studies while also considering possible compensations with recreational sedentary behaviours outside of school time. Second, we deviated from our review protocol by adding a post-hoc sample size exclusion criteria. While a deviation from protocol is not ideal, excluded participants (*n* = 3229) would have contributed less than 1% to the total number of participants. Further, excluding studies with smaller sample sizes increased our confidence that associations represent a true effect [159]. Third, while the comprehensive scope of this review can be considered a strength for informing guideline development, it may also be considered a limitation when summarizing findings. Specifically, a broad search, inclusion criteria, and outcome categories (e.g., fitness comprised of domains such as flexibility, aerobic endurance, muscular power) may have introduced heterogeneity to exposure and outcome variables, making it difficult to conduct meta-analyses. Ideally, this broad review will guide future reviews aimed at answering more narrowly focused research questions. Lastly, the gap between the last search (January 2021) and the submission (October 2021) of this review could warrant updating the search strategy. However, there was only a 6-month gap between the most recent search and the review findings informing the development of guidelines in June 2021 [27].

## Conclusions

Our findings suggest more school-related sedentary behaviour is unfavourable for other movement behaviours, but favourable for cognitive and social emotional indicators. Favourable associations between more school-related sedentary behaviour and cognitive and social emotional indicators were mainly related to homework. However, favourable associations were primarily observed for secondary school-aged children and a dose-response relationship was observed as high levels of homework were unfavourable for health and well-being indicators. Further, when displacing school-related sedentary behaviours, active lessons were the most beneficial for health and well-being. Our findings have important implications for policy makers, schools, and teachers, with regard to the amount of homework assigned and the introduction of active lessons into the classroom to enhance the learning, health and well-being of children. More research is needed examining screen-based learning and indicators of health and well-being, as well as school-related sedentary behaviours overall in low- and middle-income countries.

## Supplementary Information

**Additional file 1.**

**Additional file 2.**

**Additional file 3.**

## Data Availability

All data generated or analysed during this study are included in this published article and its supplementary information files.
