# Supplementary material for: School-related sedentary behaviours and indicators of health and well-being among children and youth: a systematic review
Source: Int J Behav Nutr Phys Act. 2022 Apr 5;19:40. doi: 10.1186/s12966-022-01258-4 (PMC8979786; doi:10.1186/s12966-022-01258-4)
Supplement: Supplementary file 1 — Additional file 1. [file 12966_2022_1258_MOESM1_ESM.docx]

| **#** | **APA PsycInfo <1806 to December Week 4 2020>** | **Results from 7 Jan 2021** | **Ovid MEDLINE(R) ALL <1946 to January 06, 2021>** | **Results from 7 Jan 2021** | **Embase <1974 to 2021 January 06>** | **Results from 7 Jan 2021** |
| --- | --- | --- | --- | --- | --- | --- |
| 1 | sedentar*.tw. | 7,433 | Sedentary Lifestyle/ | 10,141 | (sedentar$ or stationar$).tw. | 107,603 |
| 2 | ((chair or sitting or car or automobile or auto or bus or indoor or in-door or screen or computer) adj time).tw. | 1,536 | sedentar$.tw. | 33,001 | ((chair or sitting or car or auto or automobile or bus or indoor or in-door or screen or computer) adj time).tw. | 5,364 |
| 3 | (computer game* or video game*).tw. | 7,648 | ((chair or car or automobile or auto or bus or indoor or in-door or screen or computer) adj time).tw. | 3,147 | [low energy expenditure.tw.](http://expenditure.tw/) | 264 |
| 4 | ((television adj watch*) or tv watch*).tw. | 538 | [low energy expenditure.tw.](http://expenditure.tw/) | 186 | (computer game* or video game*).tw. | 6,041 |
| 5 | (Electronic game* or gaming).tw. | 5,444 | (computer game* or video game* or television or tv).tw. | 30,038 | ((television adj watch*) or tv watch*).tw. | 883 |
| 6 | (screen based entertainment or screen-based entertainment or screen time).tw. | 1,103 | (electronic game* or gaming).tw. | 3,684 | television viewing/ or computer/ | 75,194 |
| 7 | exp Social Media/ | 15,497 | Television/ or Computers/ or Video games/ | 68,624 | (screen based entertainment or screen-based entertainment or screen time).tw. | 3,011 |
| 8 | exp Mobile Devices/ | 8,427 | (screen based entertainment or screen-based entertainment or screen time).tw. | 2,420 | Social Media/ or Mobile Application/ or Mobile Phone/ | 51,824 |
| 9 | (texting or text messag* or app or apps or mobile applications).tw. | 10,997 | (texting or text messag* or app or apps or mobile applications).tw. | 35,707 | (texting or text messag* or app or apps or mobile applications).tw. | 49,093 |
| 10 | (smartphone* or smart phone* or cell phone* or mobile phone* or small screen*).tw. | 9,932 | (smartphone* or smart phone* or cell phone* or mobile phone* or small screen*).tw. | 23,940 | (smartphone* or smart phone* or cell phone* or mobile phone* or small screen*).tw. | 31,908 |
| 11 | (iphone* or ipad* or ipod* or tablet* or laptop*).tw. | 7,386 | (iphone* or ipad* or ipod* or tablet* or laptop*).tw. | 58,853 | (iphone* or ipad* or ipod* or tablet* or laptop*).tw. | 98,255 |
| 12 | bed rest.mp. or sitting.tw. | 5,784 | (social media or Facebook or Youtube or Twitter or Snapchat or Instagram or Pinterest or Skype or Vine or Zoom or Tik Tok or Google classroom or ClassDojo).tw. | 22,419 | (social media or Facebook or Youtube or Twitter or Snapchat or Instagram or Pinterest or Skype or Vine or Zoom or Tik Tok or Google classroom or ClassDojo).tw. | 30,195 |
| 13 | (homework* or recess or recesses).tw. | 5,936 | [bed rest.mp.](http://rest.mp/) | 7,383 | bed rest.mp. or sitting.tw. or (physical* adj3 inactivit*).tw. | 54,676 |
| 14 | or/1-13 | 70,449 | [sitting.tw.](http://sitting.tw/) | 23,164 | (homework* or recess or recesses).tw. | 9,204 |
| 15 | (child* or youth* or adolescent* or pediatric* or paediatric*).tw. | 911,813 | (physical* adj2 inactivit*).tw. | 8,288 | or/1-14 | 451,800 |
| 16 | 14 and 15 | 15,203 | (homework* or recess or recesses).tw. | 7,140 | (child* or youth* or adolescent* or pediatric* or paediatric*).tw. | 2,235,484 |
| 17 | Monitoring/ | 8,466 | or/1-16 | 294,883 | 15 and 16 | 43,250 |
| 18 | (activPAL* or ActiGraph* or Actical* or GENEActiv* or SenseWear* or Axivity* or acceleromet* or heartrate monit* or heart rate monit* or pedomet* or armband* or arm band* or inclinomet*).tw. | 7,857 | (child* or youth* or adolescent* or pediatric* or paediatric*).tw. | ##### | exp Physiologic Monitoring/ | 6,937 |
| 19 | (activity monitor* or activity tracker* or fitness tracker* or portable monitor* or wearable monitor* or Fitbit* or Vivofit* or Fuelband*).tw. | 1,204 | 17 and 18 | 32,419 | Accelerometer/ or Accelerometry/ | 19,059 |
| 20 | Self report/ or Questionnaires/ or Reproducibility of Results/ | 37,555 | exp Physiologic Monitoring/ | 177,735 | (activPAL* or ActiGraph* or Actical* or GENEActiv* or SenseWear* or Axivity* or acceleromet* or heartrate monit* or heart rate monit* or pedomet* or armband* or arm band* or inclinomet*).tw. | 39,782 |
| 21 | (report* or self-report* or questionnaire* or diary or diaries or scale* or interview* or journal* or teacher* report*).tw. | ##### | exp Accelerometry/ | 9,414 | (activity monitor* or activity tracker* or fitness tracker* or portable monitor* or wearable monitor* or Fitbit* or Vivofit* or Fuelband*).tw. | 7,610 |
| 22 | or/17-21 | ##### | (activPAL* or ActiGraph* or Actical* or GENEActiv* or SenseWear* or Axivity* or acceleromet* or heartrate monit* or heart rate monit* or pedomet* or armband* or arm band* or inclinomet*).tw. | 28,327 | Self report/ or Questionnaire/ or Rating Scale/ | 908,535 |
| 23 | 16 and 22 | 7,152 | (activity monitor* or activity tracker* or fitness tracker* or portable monitor* or wearable monitor* or Fitbit* or Vivofit* or Fuelband*).tw. | 5,423 | (report* or self-report* or questionnaire* or diary or diaries or scale* or interview* or journal* or teacher* report*).tw. | 6,912,960 |
| 24 | (school* or education* or teacher* or student*).mp. | ##### | Self report/ or Questionnaires/ or Reproducibility of Results/ | 873,933 | or/18-23 | 7,093,838 |
| 25 | 23 and 24 | 4,019 | (report* or self-report* or questionnaire* or diary or diaries or scale* or interview* or journal* or teacher* report*).tw. | ##### | 17 and 24 | 22,647 |
| 26 | limit 25 to yr="2014 -Current" | 2,256 | or/20-25 | ##### | (school* or education* or teacher* or student*).mp. | 2,170,404 |
|  | limit 26 to english language | 2,095 | 19 and 26 | 17,321 | 25 and 26 | 11,193 |
|  |  |  | (school* or education* or teacher* or student*).mp. | ##### | limit 27 to embase | 5,360 |
|  |  |  | 27 and 28 | 8,116 | limit 28 to yr="2014 -Current" | 3,197 |
|  |  |  | limit 29 to medline | 6,596 | limit 29 to english language | 3,096 |
|  |  |  | limit 30 to yr="2014 -Current" | 3,624 |  |  |
|  |  |  | limit 31 to english language | 3,519 |  |  |
