# Supplementary material for: School-related sedentary behaviours and indicators of health and well-being among children and youth: a systematic review
Source: Int J Behav Nutr Phys Act. 2022 Apr 5;19:40. doi: 10.1186/s12966-022-01258-4 (PMC8979786; doi:10.1186/s12966-022-01258-4)
Supplement: Supplementary file 2 — Additional file 2. [file 12966_2022_1258_MOESM2_ESM.docx]

Additional file 2

| **Supplementary Table 1: Adiposity Results Table** | | | | | |
| --- | --- | --- | --- | --- | --- |
| **Study** | **Sample** | **Study Design** | **Exposure** | **Outcome** | **Main Findings** |
| Meyer et al. 2014; Switzerland | N=289; grade 1 and 5 | Clustered RCT (duration: 9 months) | **Additional PA**  Intervention: Participants in the intervention group had two additional physical education lessons (45 minutes each) per week, had three to five short activity breaks (2-5 minutes each) during academic lessons, and received daily physical activity homework of about 10 minutes.  Control: Had normal classes, with weekly physical education lessons. | Three adiposity indicator outcomes were used: BMI z-scores were calculated using age- and sex-specific norms; Waist circumference; and the sum of the triceps, biceps, subscapular, and suprailiac skinfolds, measured to the nearest 0.5mm with callipers. | **Additional PA**  No significant intervention effects were observed for BMI (Mean difference: 0.01, 95%CI: -0.130, 0.151, p = 0.88), waist circumference (Mean difference: -0.051, 95%CI: -0.195, 0.092, p = 0.48), or the sum of 4 skinfolds (Mean difference: 0.076, 95%CI: -0.222, 0.069, p = 0.3) at 3 year follow-up. |
| Muller et al 2016; Germany | N=236; 11.5 years | Clustered RCT (duration: 4 years) | **Recess/PE**  Intervention: Participants in the intervention group received additional physical exercise daily, lasting 45 minutes, with at least 15 minutes of endurance training.  Control: Continued to receive normal classes, with two PE classes/week. | BMI z-scores were calculated using age- and sex-specific norms and percentiles were analyzed. | **Recess/PE**  No significant intervention effect was observed for BMI percentiles (Group difference: 1.66, 95%CI: -6.02, 9.35, p = 0.0638). |
| Parrish et al. 2018; Australia | N=88; 14.71 years | Clustered RCT (duration: 5 months) | **Standing desk**  Intervention: Participants in the intervention group received new furniture during classes, designed to reduce sitting time. In addition, standing strategies were implemented, such as requiring students to spend 30 minutes/day at standing desks. Also, structured learning modules were part of lessons, supporting behaviour change, and optional standing strategies such as outdoor lessons were included.  Control: Continued to receive normal classes. | BMI was calculated and analyzed. Body fat percentage was measured using bioelectrical impedance. | **Standing desk**  No significant intervention effects were observed for BMI (Mean difference (intervention  -control): 0.39 ±0.96) or body fat percentage (Mean difference (intervention  -control): 1.60 ±4.22). |
| Seljebotn et al. 2019; Norway | N=447; 9 to 10 years | Clustered RCT (duration: 10 months) | **Additional PA**  Intervention: Participants in the intervention group received additional physical activity lessons, physical activity homework, and physically active recesses. Active lessons of 45 minutes were included 2-3 times/week, on days without physical education. Active homework of 10 minutes/day was assigned by teachers, and physically active recesses (10min/day) were encouraged.  Control: Continued to receive normal classes. | BMI was calculated and analyzed. | **Additional PA**  No significant intervention effects were observed for BMI for the whole sample (Mean difference: 0.1, 95%CI: -0.4, 0.3, p = 0.152), girls (Mean difference: 0.2, 95%CI: -0.1, 0.4, p = 1.46), or boys (Mean difference: 0.1, 95%CI: -0.2, 0.3, p = 0.584). |
| Contardo Ayala et al. 2016; Australia | N=41.0; 11 to 12 years (control=11.7, intervention=11.5) | Non-Randomized Intervention (duration: 8 months) | **Standing desk**  Intervention: Each participant in the intervention class was provided with a manually adjustable height-adjustable workstation.  Control: The control classroom followed standard practice using traditional furniture. | BMI z-scores were calculated using age- and sex-specific norms. Waist circumference z-scores were also analyzed. | **Standing desk**  No significant intervention effect was observed for BMI z-score or waist circumference z-score, but the BMI z-score significantly decreased on the control group only in the follow-up (Mean difference: 0.07. 95%CI: -0.23, 0.38). The waist circumference z-score significantly increased in the intervention group only (Mean difference: 0.21, 95%CI: -0.05, 0.48). |
| Contardo Ayala et al. 2018; Australia | N=88; 14.8 years | Non-Randomized Intervention (duration: 17 weeks) | **Standing desk**  Intervention: traditional classroom furniture in the classroom was replaced with height-adjustable desks (Learnfit, Ergotron Inc., Minnesota, USA) and lab stools (Furnware Bodyfurn Lab stool, New Zealand) for every student and the teacher. Additional supportive prompts were also proposed, with messages explaining the health impacts of excessive sitting, breaking up sitting every 15 minutes, with tips and strategies to reduce and break classroom sitting, and how to properly adjust desks.  Control: followed standard pedagogical (usual) practice  in classrooms using traditional `seated' furniture. | BMI and waist circumference were analyzed. | **Standing desk**  A significant intervention effect was observed, favoring the intervention group for BMI at 4 weeks (Mean difference: 0.3, 95%ci: 0.1, 0.6), but not at 17 weeks (Mean difference: 0.3, 95%ci: -0.1, 0.6). Significant intervention effects were observed favoring the intervention group at 4 weeks (Mean difference: -3.5, 95%ci: -6.3, -0.8) and 17 weeks (Mean difference: -2.6, 95%ci: -5.0, -0.3) for waist circumference. |
| Chesham et al. 2018; Scotland | N=371; 8.4 years | Non-Randomized Intervention (duration: 7 months for intervention, 3 months for control) | **Active breaks**  Interventions: participants went outside to walk or run one mile per day, at a time of the classroom teacher’s choosing, in addition to scheduled breaks and physical education classes. The one mile was usually carried out in the football field or playground area, and happened on most days, regardless of weather conditions.  Control: the control group followed their usual curriculum. | Triceps, biceps, iliac crest and subscapular  Skinfolds were measured and the sum of skinfolds was analyzed (mm). | **Active breaks**  A significant intervention effect was observed, favouring the intervention group for the sum of skinfolds (Mean difference in change: -1.4, 95%CI: -2.0, -0.8, p = 0.0260). |
| Cronholm et al. 2018; Sweden | N=228; 7.7 years | Non-Randomized Intervention (duration: average follow-up of 7 years) | **Recess/PE**  Intervention: the intervention programme increased the level of physical education from 60 minutes a week to 200 minutes a week, provided daily 40-minute classes during all the compulsory school years.  Control: control schools followed the normal school national curriculum. | BMI was calculated and classified children as overweight or obese using age- and sex-specific growth curves. | **Recess/PE**  No significant differences were observed in intervention and control groups among boys or girls for BMI and occurrence of overweight or obesity (p > 0.05). |
| Lee et al. 2018; South Korea | N=884; 11.0 years | Longitudinal (follow-up: 1 year) | **Homework**  Participants reported how much time they spent studying after school (<1h, 1~3h, ≥3h). | Weight status was defined based on BMI as overweight ( = 85th percentile), obese ( = 95th percentile or 25 kg/m 2 ), and normal weight (<85th percentile). | **Homework**  Participants who developed obesity during follow-up spent greater amounts of time studying after school compared to those without obesity (p = 0.046).  Those who spent ≥3h of studying after school had higher odds for obesity compared to those who studied <1h (Odds Ratio: 13.3, 95%CI: 1.76, 100.27, p = 0.012). No significant associations were observed for the odds of obesity when comparing 1-3h of studying with <1h (OR: 3.71, 95%CI: 0.64, 21.62, p = 0.145). And no significant associations were observed for the odds of overweight when studying for 1-3h (OR: 1.59, 95%CI: 0.44, 5.72, p = 0.482) and ≥3h (OR: 5.1, 95%CI: 0.99, 26.4, p = 0.052) with the <1h as reference. |
| Maume 2017; USA | N=974; 12 years | Longitudinal (follow-up: 3 years) | **Homework**  Participants reported how much time they spent doing homework during the week. | BMI was calculated and percentiles were analyzed. | **Homework**  No significant associations were observed between change in homework from the age 12 to age 15, and BMI percentile at age 15, and between homework at ate 12 and BMI percentile at age 15. |
| Rutten, Boen, and Seghers 2014; Belgium | N=472; 10.97 years | Longitudinal (follow-up: 2 years) | **Homework**  Participants reported how many hours per day they typically spent doing homework on weekdays and weekend days, and a weighted time per week variable was calculated. | BMI was calculated and used to classify the weight status of the participants using age- and sex-specific growth curves. | **Homework**  No significant association between homework and weight status from baseline to follow-up were observed, and no interactions of sex, time, and weight status were observed either for the same period (p = 0.792).  A significant interaction was observed between time and weight status in the association between homework and weight status from baseline to follow-up (p = 0.036), showing that normal-weight children increased their academically relevant SB more (+2.83 hours/week) compared with overweight or obese children (+0.90 hours/week). |
| Cheung et al. 2019; USA | N=905 schools (approx 524700); Grade 1 to 5 | Cross-Sectional | **Active breaks & Additional PA & Recess/PE**  Participants answered questions on frequency and duration of PE classes and recesses, physical activity during the in-class time, and presence or absence of physical activity opportunities before and after school. | BMI was calculated and weight status was classified. | **Active breaks & Additional PA & Recess/PE**  For in-class physical activity, no significant associations were found with healthy BMI classification among boys (PR: 1.00, 99%CI: 0.97, 1.02) or girls (PR: 1.00, 99%CI 0.97, 1.02).  For in-school physical activity, no significant associations were found with healthy BMI classification among boys (PR: 1.01, 99%CI: 0.99, 1.02) or girls (PR: 1.00, 99%CI 0.99, 1.02).  For PE-based physical activity, no significant associations were found with healthy BMI classification among boys (PR: 1.00, 99%CI: 0.99, 1.02) or girls (PR: 1.00, 99%CI 0.99, 1.01).  For recess-based physical activity, no significant associations were found with healthy BMI classification among boys (PR: 1.01, 99%CI: 0.99, 1.03) or girls (PR: 1.01, 99%CI 0.99, 1.03). |
| Compernolle et al. 2018; Belgium | N=513; 15 years | Cross-Sectional | **Homework**  Participants reported how many hours per day they typically spent doing homework on weekdays and weekend days. | BMI was calculated and weight status was classified. | **Homework**  No significant differences in the proportion of time spent on homework during weekdays and weekend days were observed when comparing healthy weight and overweight/obese groups. |
| da Costa et al. 2018; Brazil | N=415; 12.3 years | Cross-Sectional | **Stationary time/sedentary time**  Participants wore accelerometers on their right hip during school time and sedentary bout frequency and total volume of sedentary behaviour in bouts were calculated for different bout lengths (at least 5-, 10-, 30-, and 60-minute bouts). | BMI was calculated and used to classify the weight status of the participants using age- and sex-specific growth curves. | **Stationary time/sedentary time**  No significant differences were found for time spent in sedentary/stationary behaviour bouts or frequency of sedentary behaviour bouts of any duration between weight status categories. |
| Farajian et al. 2014; Greece | N=4965; Grade 5 to 6 (Normal weight=10.93 years; overweight/obese =10.87 years) | Cross-Sectional | **Homework**  Participants reported how many hours per day they typically spent doing homework on weekdays and weekend days. | BMI was calculated and used to classify the weight status of the participants using age- and sex-specific growth curves. | **Homework**  Time spent on studying on weekdays was associated with higher odds of overweight/obesity, compared to normal weight (OR: 1.07, 95%CI: 1.02, 1.13), while no significant differences were found for time spent studying during the weekend between overweight/obese and normal-weight groups. |
| Gu et al. 2020; USA | N=374; 9.64 years | Cross-Sectional | **Stationary time/sedentary time**  Stationary time was measured with wrist-worn accelerometers, and minutes spent in sedentary/stationary behaviour were calculated. | BMI was calculated and analyzed. | **Stationary time/sedentary time**  A significant positive association was observed between sedentary/stationary time and BMI (Regression coefficient: 0.04, p < 0.05). |
| Hubbard et al. 2016; USA | N=453; 9.1 years | Cross-Sectional | **Stationary time/sedentary time**  Stationary time was measured with waist-worn accelerometers, and minutes spent in sedentary/stationary behaviour were calculated for each participant across four segments: total daily time (sum of minutes across all valid days divided by the number of valid days), school time, weekday out-of-school time, and weekend time. School-time hours were calculated for each participant, based on the specific start and end times of the school day for each day the accelerometer was worn. | BMI was calculated and used to classify the weight status of the participants using age- and sex-specific growth curves. | **Stationary time/sedentary time**  No significant associations between sedentary/stationary time and overweight/obese classification, compared to normal/under weight classification (p >= 0.05). |
| Ishihara et al. 2018; Japan | N=325; 12 to 13 years | Cross-Sectional | **Homework**  Learning duration was assessed using the time spent on learning after school on weekdays and weekends, and the use of a cram school or private teacher. | BMI was calculated and analyzed. | **Homework**  No significant associations were observed between BMI and learning duration for the whole sample, females, or males (p > 0.05). |
| Kantanista and Osinski 2014; Poland | N=3249; 14 to 16 years | Cross-Sectional | **Recess/PE**  Participation in PE classes was reported by the participants using the question: “How often have you participated in PE classes at school during recent several months?” The possible answers ranged from “I have participated in every or nearly every PE class” to “I have not participated in PE classes at all”.  **Homework**  Participants reported how many hours per day they typically spent doing homework on weekdays and weekend days, and a weighted time per week variable was calculated. | BMI was calculated and used to classify the weight status of the participants using age- and sex-specific growth curves. | **Recess/PE**  Lower participation in PE lessons was observed among overweight girls compared to normal weight girls and among overweight girls compared to underweight girls. Among boys, no differences were observed between overweight and underweight boys, but overweight boys participated less in PE classes compared to normal-weight boys.  **Homework**  Boys who were overweight spent more total time doing homework, and time during weekdays and weekend days compared to normal-weight peers. No differences were found between underweight boys and normal-weight boys.  Among girls, total time spent doing homework, time doing homework during weekdays, and weekend days did not differ between weight status subgroups. |
| Khan et al. 2019; Bangladesh | N=2989; 14.2 years | Cross-Sectional | **Recess/PE**  Participants reported their attendance in physical education glasses in the last 7 days. | BMI was calculated and used to classify the weight status of the participants using age- and sex-specific growth curves. | **Recess/PE**  When the whole sample was analyzed, occasionally attending PE classes (OR: 0.483, 95%CI: 0.284, 0.822) and regularly attending PE classes (OR: 0.592, 95%CI: 0.327, 0.682) were associated with lower odds of overweight/obesity, compared to never attending PE classes (reference). When only males were analyzed, occasionally attending PE classes (OR: 0.42, 95%CI: 0.281, 0.627) and regularly attending PE classes (OR: 0.488, 95%CI: 0.330, 0.722) were associated with lower odds of overweight/obesity, compared to never attending PE classes (reference). For females, occasionally attending PE classes (OR: 0.445, 95%CI: 0.266, 0.627) was associated with lower odds of overweight/obesity, compared to never attending PE classes (reference). However, no differences were observed between never attending PE classes and regularly attending PE classes (OR: 0.745, 95%CI: 0.461, 1.234). |
| Magriplis et al. 2019; Greece | N=4434; 10.9 years | Cross-Sectional | **Homework**  Participants reported how many hours per day they typically spent doing homework on weekdays and weekend days. | BMI was calculated and used to classify the weight status of the participants using age- and sex-specific growth curves. | **Homework**  A significant association was observed between study hours and higher odds of overweight/obese, compared to a healthy weight (OR: 1.06, 95%CI: 1.01, 1.12). |
| Michaud et al. 2015; Canada | N=511; 9.6 years | Cross-Sectional | **Homework**  Participants reported how many hours per day they typically spent doing homework on weekdays and weekend days. Preoccupations linked to schoolwork were also reported, using the following question: “During the past 3 months, have you been worried or stressed by schoolwork?”. | BMI was calculated and percentiles were also calculated using age- and sex-specific growth curves. BMI percentiles and z-score were analyzed.  Total fat mass and trunk body fat were measures using dual-energy x-ray absorptiometry. | **Homework**  When all boys were analyzed, no differences were found for BMI percentiles between those who spent <30min/day in homework and those who spent ≥30min/day in homework (p = 0.322). However, significant differences were found for fat mass (p = 0.013) and trunk body fat (p = 0.013), with those who spent ≥30min/day doing homework showing higher fat mass and trunk body fat.  When boys who were not stressed about homework were analyzed, no differences between those who spent <30min/day in homework and those who spent ≥30min/day in homework were observed for BMI percentiles (p = 0.373), fat mass (p = 0.258), and trunk body fat (p = 0.248).  For boys who reported being stressed about homework, significant differences were observed, with those who spent ≥30min/day in homework having higher BMI percentiles (p = 0.026), higher total fat mass (p = 0.001), and higher trunk body fat (p = 0.001) compared to those who spent <30min/day in homework.  When all girls were analyzed, no differences between those who spent <30min/day in homework and those who spent ≥30min/day in homework were observed for BMI percentiles (p = 0.471), fat mass (p = 0.461), and trunk body fat (p = 0.416). No significant associations were observed between homework time and BMI percentiles, total fat mass, and trunk body fat when girls stressed about homework and girls not stressed about homework were analyzed separately. |
| Pengpid and Peltzer 2015; Fiji, Kiribati, Samoa, Solomon Islands, Tonga, Vanuatu | N=10424; 13 to 16 years | Cross-Sectional | **Recess/PE**  Participants reported their attendance in physical education, and active commuting to school in the seven days preceding the study. | BMI was calculated and used to classify the weight status of the participants using age- and sex-specific growth curves. | **Recess/PE**  A significant association was observed with attending PE classes 3 or more times per week and odds of overweight status, compared to attending less than 3 times per week (OR: 1.23, 95%CI: 1.00, 1.50). No significant associations were observed when odds of obesity were the outcome (OR: 0.23, 95%CI 0.61, 1.11), or when odds of obesity and overweight were compared simultaneously to normal weight (OR: 1.08, 95%CI: 0.94, 1.25). |
| Pengpid and Peltzer 2019; Indonesia, Lao PDR, Philippines, Thailand, Timor-Leste | N=30145; 14 years | Cross-Sectional | **Recess/PE**  Participants reported their attendance in physical education. | BMI was calculated and used to classify the weight status of the participants using age- and sex-specific growth curves. | **Recess/PE**  No significant associations were observed between attendance to PE classes and underweight status (RR: 0.94, 95%CI: 0.80, 1.10), or overweight status (RR: 0.90, 95%CI: 0.74, 1.08) compared to normal weight status. |
| Rajala et al. 2019; Finland | N=420; 13.7 years | Cross-Sectional | **Stationary time/sedentary time**  Stationary time was measured with hip-worn accelerometers, and minutes spent in sedentary/stationary behaviour were calculated. Time spent in stationary time during the whole day and school time were calculated. | BMI was calculated and analyzed. | **Stationary time/sedentary time**  No significant association was observed between in-school stationary time and BMI (Regression coefficient: 0.01, SE: 0.04, p > 0.05). |
| Ren et al. 2017; China | N=5032; 9.7 years | Cross-Sectional | **Homework**  The parents of the participants reported how many hours per day their children typically spent doing homework on weekdays and weekend days, and how many hours of remedial academic work, or studying to supplement and enrich their academic activities outside of school hours, their children engaged in during weekdays and weekend days. | The parents of the participants reported their child’s current height and weight based on results from their most recent clinic visit or latest physical examination. BMI was calculated and used to classify the weight status of the participants using age- and sex-specific growth curves. BMI z-scores were also analyzed. | **Homework**  When all participants were analyzed, BMI z-scores was significantly associated with homework time on weekdays (B: 0.007, SE: 0.002, p = 0.004), but not with remedial academic work during weekdays (B: -0.03, SE: 0.02, p > 0.05), homework time during weekend days (B: 0.005, SE: 0.002, p > 0.05), or with remedial academic work during weekend days (B: 0.003, SE: 0.001, p > 0.05).  Among boys, BMI was significantly associated with time spent on homework during weekdays (B: 0.009, SE: 0.03, p = 0.002), but not significantly associated with weekday remedial academic work (B: -0.04, SE: 0.03, p > 0.05), homework on wekeend days (B: 0.01, se: 0.02, p > 0.05), or with remedial academic work during wekeend days (B: 0.02, SE: 0.02, p > 0.05).  Among girls, BMI z-scores were not significantly associated with time spent on homework during weekdays (B: 0.01, SE: 0.03, p > 0.05) or weekend days (B: -0.01, SE: 0.02, p > 0.05), or with remedial academic work during weekdays (B: -0.01, SE: 0.03, p > 0.05) or weekend days (B: -0.002, SE: 0.02, p > 0.05). |
| Sanders et al. 2019; Australia | N=4013; 10 to 15 years | Cross-Sectional | **Homework**  Participants reported in a time-use diary how much time they spent on education-related screen time. On the day following the diary completion, an interviewer confirmed the reports and added contextual information (e.g., where participants were). | Waist circumference was measured and analyzed. | **Homework**  No association between education screen time and waist circumference (standardized B: -0.008, 95%CI: 0.023, -0.039). |
| Sigmund et al. 2014; Czech Republic | N=338; 9.91 years | Cross-Sectional | **Stationary time/sedentary time**  Stationary time was measured with hip-worn accelerometers (Actigraph ActiTrainer), and minutes spent in sedentary/stationary behaviour were calculated. Time spent in stationary time during the school day, recesses, PE classes, and non-PE classes were recorded and analyzed. | BMI was calculated and used to classify the weight status of the participants using age- and sex-specific growth curves. | **Stationary time/sedentary time**  For boys, no differences were observed between normal-weight participants compared to overweight/obese participants for non-PE classes stationary time (Effect size (d): 0.17), for PE-classes stationary time (Effect size (d): 0.02), and for in-school stationary time (Effect size (d): 0.21), however, normal-weight participants spent more time in stationary time during recesses compared to overweight/obese participants (Median: 15.38 vs 11.88 minutes/recess, Effect size (d): 0.35).  For girls, no differences were observed between normal-weight participants compared to overweight/obese participants for non-PE classes stationary time (Effect size (d): 0.10), for PE-classes stationary time (Effect size (d): 0.01), for recess stationary time (Effect size (d): 0.25), and for in-school stationary time (Effect size (d): 0.10). |
| van Stralen et al. 2013; Belgium, Greece, Hungary, Netherlands, Switzerland | N=1025; 11.6 years | Cross-Sectional | **Stationary time/sedentary time**  Stationary time in school was measured with hip-worn accelerometers (ActiGraph ActiTrainers (triaxial), GT3Xs (triaxial) or GT1Ms). | BMI was calculated and used to classify the weight status of the participants using age- and sex-specific growth curves. | **Stationary time/sedentary time**  No association between the percent of time spent stationary in school and weight status in Belgium, Greece, Hungary, Switzerland, and for the total sample. However, a positive association between stationary time and weight status was observed in the sample from the Netherlands, with overweight children spending more time in stationary behaviour (B: 3.6, SE: 1.58, p < 0.05). |
| Xue et al. 2016; China | N=1586; 10.0 years | Cross-Sectional | **Homework**  Homework time was reported by participants 9 and older or by the participant’s parents for children younger than 9. Frequency and duration of homework were reported, and for children 9 and older, a face-to-face interview was conducted and participants were asked homework duration and frequency separately for weekdays and weekend days. A weighted time per week variable was calculated, and proportions of participants spending more than 1 or 2 hours per day on homework were calculated. | Participants had their height, weight, and skin-folds measured, and body fat percent was calculated using appropriate equations. Fat mass index was also calculated (FMI (kg/m2) = (weight x %BF)/height2). | **Homework**  Time spent on homework was significantly associated with higher fat mass index among boys (p = 0.04) and girls (p = 0.003), and significantly associated with higher percent body fat in boys (p = 0.03) and girls (p = 0.003). |
| Zhang et al. 2016; China | N=3766; 8.5 years | Cross-Sectional | **Homework**  Participants reported how many hours per day they typically spent doing homework. | BMI was calculated and used to classify the weight status of the participants using age- and sex-specific growth curves. | **Homework**  No significant association was observed between homework time and odds of overweight (OR: 1.04, 95%CI: 0.90, 1.19), but a significant association was observed between homework time and increased odds of obesity (OR: 1.24, 95%CI: 1.08, 1.43). |
| Zhang et al. 2017; China | N=17318; 9.18 years | Cross-Sectional | **Homework**  Participants reported how many hours per day they typically spent doing homework. | BMI was calculated and used to classify the weight status of the participants using age- and sex-specific growth curves. | **Homework**  No significant associations were observed between spending 2 or more hours of homework per day (OR: 1.11, 95%CI: 0.94, 1.31) and 1-2 hours of homework per day (OR: 1.01, 95%CI: 0.78, 1.31) and odds of obesity, compared to spending less than one hour on homework per day. |

| **Supplementary Table 2: Biomarkers Results Table** | | | | | |
| --- | --- | --- | --- | --- | --- |
| **Study** | **Sample** | **Study Design** | **Exposure** | **Outcome** | **Main Findings** |
| Meyer et al. 2014; Switzerland | N=289.0; Grades 1 and 5 | Clustered RCT (Follow-up: 3 years) | **Additional Physical Activity**  Intervention: The intervention group had two additional physical education lessons (45 minutes each) on the remaining school days that were taught by physical education teachers. Three to five short activity breaks (two to five minutes each) were introduced every day during academic lessons. Children also received daily physical activity homework of about 10 minutes.  Control: Children in both groups had three physical education lessons per week (45 minutes each) given by the usual classroom teachers. | **Systolic and diastolic blood pressure** measured five times after a resting period of five minutes using an automated oscillograph (Oscillomate, CAS Medical Systems, Branford, CT, USA). The mean of the three measurements with the smallest variation was taken and then z-transformed. **Blood glucose, insulin, high-density lipoprotein-cholesterol (HDL), and triglycerides** drawn in the morning while fasting and a **composite cardiovascular risk score** computed by averaging the z-scores of all components of the metabolic syndrome (waist circumference, blood pressure (mean of systolic and diastolic blood pressure z-score), glucose, inverted high density lipoprotein-cholesterol (HDL), and triglycerides. Skewed data were ln-transformed. Z-scores  were derived from published age- and gender-specific norm  values for BMI and blood pressure, with the other components z-transformed using sample specific grade and gender  means and standard deviations at  each measurement period. | **Additional Physical Activity**  No differences between intervention and control groups for **composite cardiovascular risk score (**Adjusted mean difference: -0.003; 95%CI: -0.208, 0.201), systolic blood pressure (Adjusted mean difference: 0.279; 95%CI: -0.001, 0.559), diastolic blood pressure (Adjusted mean difference: 0.146; 95%CI: -0.054, 0.347), glucose (Adjusted mean difference: -0.004; 95%CI: -0.401, 0.394), HDL (Adjusted mean difference: 0.147; 95%CI: -0.154, 0.449), and triglycerides (Adjusted mean difference: 0.143; 95%CI: -0.265, 0.550) at 3 years follow-up. |
| Muller et al 2016; Germany | N=236.0; Mean age = 11.5 years | Clustered RCT (Follow-up: 4 years) | **Recess/Physical Education**  Intervention classes were assigned to daily physical exercise (45 minutes) with at least 15 minutes of endurance training per school day. Totaling 5 units of physical education.  Control: Two units of physical education (45 min/unit) per week. | **Systolic blood pressure, as well as fasting low-density lipoprotein-cholesterol (LDL), HDL, and triglycerides**. No further information given on the measurement of these biomarkers. | **Recess/Physical Education**  No differences between intervention and control for systolic blood pressure (Mean difference: 0.97; 95%CI: -6.8, 8.7), HDL (Mean difference: -0.024; 95%CI: -0.17, 012), LDL (Mean difference: -0.11; 95%CI: -0.34, 0.13), or triglycerides (Mean difference: 0.023; 95%CI: -0.14, 019) at 4 years follow-up. |
| Contardo Ayala et al. 2016; Australia | N=41; age range: 11-12 years (mean age: intervention= 11.5 years, control=11.7 years) | Non-Randomized Intervention (Follow-up: 8 months) | **Standing desk**  Intervention: Each participant in the intervention class was provided with a manually adjustable height-adjustable workstation (LearnFit, Ergotron Pty Ltd., Sydney, Australia).  Control: The control class followed standard pedagogical practice and retained traditional classroom furniture. | **Systolic and diastolic blood pressure** average from three measurements using the automated OMRON HEM-907 in a seated position after resting for two minutes. | **Standing desk**  No differences between intervention and control groups for systolic (Mean difference:1.16; 95%CI: -2.7, 5.02) or diastolic (Mean difference: -2.13; 95%CI: -5.56, 1.29) blood pressure at 8 months. |
| Gopinath et al. 2013; Australia | N=698.0; mean age: 6.7 years | Longitudinal (Follow-up: 5 years) | **Homework**  Time spent doing homework was measured by: (1) “How many hours a day do you usually spend....” Response options were <1, 1-2, 3-4, 5-6, and =7 h/d. Responses were categorized as =2 h/d or >2 h/d for analysis. | **Systolic and diastolic blood pressure** using the automated OMRON HEM-907 in a seated position after resting for five minutes. Three separate measurements were taken and averaged for analysis at a 5-yr follow-up, while only a single measure of was taken at the baseline examination. **Mean arterial blood pressure** was calculated as one-third of the systolic plus two-thirds of the diastolic BP. | **Homework**  Time spent doing homework was not associated with changes in systolic (B: -1.12; 95%CI: -2.45, 0.21) diastolic (B: -0.48; 95%CI: -1.63, 0.67), or mean arterial (B: -0.68; 95%CI: -1.75, 0.39) blood pressure at 5 years. |

| **Supplementary Table 3: Cognitive Study Results** | | | | | |
| --- | --- | --- | --- | --- | --- |
| **Study** | **Sample** | **Study Design** | **Exposure(s)** | **Outcome(s)** | **Main Findings** |
| Altenburg, Chinapaw, and Singh 2016; Netherlands | N=56; 11.6 years | RCT  (duration: one morning) | **Active breaks**  Intervention group A: no physical activity (i.e., siting all morning working on simulated school tasks, control group);  Intervention group B: one 20-min physical activity bout halfway in the morning;  Intervention group C: two 20-min physical activity bouts, one at the start and one halfway in the morning. | Selective attention measures with the *Sky Search* subtest of the Test of Selective Attention in Children (TEA-Ch). Selective attention was measured at base-line (T0), after 20 min (T1; i.e., after the first physical activity bout in group); C), 110 min (T2; i.e., before the second bout in group); C), 130 min (T3; i.e., after the second physical activity bout in group); C) and at the end of the morning (T4; i.e., after 220 min). | **Active breaks**  Children with two activity bouts (C) had better selective attention compared to the sedentary control (A) (Unstandardized difference: -0.26, 95%CI: -0.45, -0.004), and no difference was observed between one activity bout (B) and the sedentary control group (A) for children's selective attention (Unstandardized difference: 0.06, 95%CI: -0.23, 0.36).  Children with activity bouts at the beginning of the morning (C) had better selective attention compared to the sedentary control and the group with one bout halfway in the morning (A+B) (Unstandardized difference: -0.29, 95%CI: -0.45, -0.13) |
| Pitchford 2015; Malawi | N=318; 8.08 years | RCT (duration: 8 weeks) | **Screen Time**  Intervention: participants used a tablet and math-related apps (onebillion©: Masamu (Chichewa for Maths) 1, Masamu 2, Count to 10, and Count to 20), based on the National Primary Curriculum related to core mathematical concepts in Malawi.  Placebo: participants used tablets for other educational but non-math related apps (Music Sparkles developed by Kids Game Club©, Drawing Pad developed by Darren Murtha Design©, and Toca Tailor and Toca Hair Salon developed by Toca Boca AB©).  Control: participants had normal instruction practice. | Outcomes related to mathematical ability were assessed. Math curriculum knowledge was evaluated by a test composed by 50 quiz items, taken from the apps that are specific to the Math intervention. A paper version was also used as a post-test only assessment with Math curriculum knowledge test. A Math concepts test, consisting of 48 questions assessing the conceptual understanding of mathematics such as symbolic understanding, counting, number sense, simple and complex addition, was also applied. A combined measure of mathematical ability (math curriculum knowledge generalization), using mean scores of the math concepts and the math curriculum tests was also assessed. | **Screen Time**  For students on standard 1, no intervention effects were observed for any of the mathematical abilities investigated.  For students on standard 2, significant intervention effects were observed for mathematical concepts, with students in the math app group outperforming the normal practice group (p = 0.033, Bonferroni post hoc analyses), but no differences between other groups (math app and non-math app groups p = 0.585; non-math app and normal practice groups p = 0.657).  In relation to math curriculum knowledge, the math apps group outperformed both the non-math app (p = 0.024) and the normal practice groups (p < 0.001), and no difference was found between the non-math apps and normal practice groups (p = 0.115).  For math curriculum knowledge generalization, the math app group outperformed the non-math app group (p=0.015), but no other differences were observed (non-math app and normal practice groups p= 0.474; math app and normal practice groups p = 0.497).  For students on standard 3, no significant differences in groups were observed for mathematical concepts. For math curriculum knowledge, the math apps group outperformed the non-math app (p < 0.001) and normal practice groups (p < 0.001) groups, but no difference was found between non-math app and normal practice groups (p = 0.996). Lastly, for math curriculum knowledge generalization, the math apps group outperformed the non-math app (p < 0.001) and normal practice groups (p < 0.001) groups, while the normal practice group outperformed the non-math app group (P < 0.001). |
| Pitchford and Outhwaite 2019; Malawi | N=241; 8.3 years | RCT  (duration: 8 weeks) | **Screen Time**  Intervention: participants used a tablet and math-related apps, such as for addiction and counting.  Placebo: participants used tablets for other activities such as music and drawing, but not math-related apps.  Control: participants had standard mathematical practice. | Manual processing speed was measured by a test where children were required to tap a green box displayed on the touch-screen continually using the index finger on their dominant hand, as fast as they could, which caused a blue balloon to increase in size. The task was complete when the child had tapped the green box 30 times causing the balloon to pop. An overall measure of manual processing speed was calculated from the mean completion time across the two trials.  Mathematics related curriculum and conceptual knowledge were assesses using a test consisting of 98 items. The conceptual questions were based on the Early Grade Mathematics Assessment and the Numerical Operations subtest of the WIAT-II. An overall mathematics score was determined from the total number of questions answered correctly.   Visual attention was measured by a test in which children were shown a single-colored dot, followed by an array of either 8, 12, or 16 same-colored dots. Then they were instructed to touch the dots as fast as possible. In the experimental trials, children were required to distinguish and touch all the colored dots presented in the practical trial from a display of different colored distractor dots. For each trial, time taken to complete the baseline trial was subtracted from the time taken to complete the experimental trial, thus generating a measure of visual attention that was not confounded by manual processing speed. An overall measure of visual attention was derived from the mean response times taken to complete the three experimental trials.  Short-term memory was assessed by a test in which children were presented with a three-by-three grid of yellow circles. A virtual instructor demonstrated the pattern to be recreated by the child by touching the yellow circles. When the demonstrator touched a yellow circle, it turned red, momentarily, until the demonstrator touched the next circle in the sequence. Children were then required to repeat the order they had been presented. The number of circles in the sequence increased in line with progression through the test; starting at one and increasing to nine. The task discontinued after three successive incorrect trials. An overall measure of short-term memory was indicated by the raw number of trials completed correctly. | **Screen Time**  No significant intervention effects were observed for manual processing speed, short-term memory.  A positive intervention effect, favoring the math app group, was observed for visual attention and mathematics. Also, there were no differences between placebo and control groups for visual attention of mathematics. |
| Aadland et al. 2019; Norway | N=1129; 10.2 years | Clustered RCT  (duration: 7 months) | **Additional PA**  Intervention: An additional 165 min/week of PA: (1) physically active educational lessons (3 × 30 min/week) in the subjects Norwegian, mathematics, and English, (2) PA breaks during classroom lessons (5 min/school day), and (3) PA homework (10 min/school day).  Control: typical amount of PA. | Cognitive flexibility measured with Verbal Fluency test;  Response inhibition measure with the Stroop Color Word test;  Working memory measured with the Wechsler Intelligence Scale for Children fourth edition, Digit Span test;  Cognitive flexibility, measured with the Trail Making Test Part B;  Executive functions composite score calculated using the mean of standardized scores for inhibition, working memory, and cognitive flexibility. | **Additional PA**  No intervention effect was observed for cognitive flexibility (Standardized intervention effect: 0.17, 95%CI: -0.31, 0.64, p = 0.491), response inhibition (Standardized intervention effect: 0.31, 95%CI: -0.32, 0.95, p = 0.333), Working memory (Standardized intervention effect: 0.01, 95%CI: -0.18, 0.21, p = 0.893), cognitive flexibility (Standardized intervention effect: 0.63, 95%CI: -4.26, 3.01, p = 0.736), and composite score of executive functions (Standardized intervention effect: 0.06, 95%CI: -0.03, 0.16, p = 0.191). |
| Bartholomew et al. 2018; USA | N=5432; Grade 4 | Clustered RCT  (duration: 15 minutes) | **Active lessons**  The I-CAN! program consisted of active lessons in the classroom for 10–15 min for 5 days during the school week.  Control: traditional sedentary lesson. | Time on task was measured with direct observation using five-second momentary time sampling for 15-min prior to a physically active lesson and for 15-min immediately following the active lesson. | **Active lessons**  A significant intervention effect on time on task on the intervention group. Moreover, the dose of PA (steps) during the intervention was positively associated with the increase in time on task (Standardized coefficient: 5.53, SE = 1.73). |
| Calvert et al. 2019; USA | N=156; 10 to 12 years | Clustered RCT  (duration: one school day) | **Active breaks**  Intervention: Classrooms were randomized to receive two different PA conditions, consisting of an exercise video that lasted 10 minutes, selected from the *Fresh Start Fit* GoNoodle channel. Conditions were created as follows: 1) sedentary control (SED) condition comprised of two control videos; 2) light (LIGHT) condition comprised of one control video followed by the light to moderate intensity video; 3) moderate (MOD) condition comprised of one control video followed by a high intensity video; and 4) vigorous (VIG) condition comprised of two high intensity videos.  Control: For the control condition, videos included a social studies lesson and two reading/grammar lessons from the Blazer Fresh GoNoodle channel. | Inhibitory control and attention were measured with the  Flanker Test, part of the NIH Toolbox.  Processing speed was measured with the Pattern Comparison Test, part of the NIH Toolbox.  Episodic memory was measured with the Picture Sequence Test, part of the NIH Toolbox.  Cognitive flexibility was measured with the Dimensional Change Card Sort Test, part of the NIH Toolbox. | **Active breaks**  For analyses, the sedentary group was only compared to the vigorous group. No intervention effect was observed between vigorous and sedentary (control) conditions for inhibitory control (Baseline to Post-test change: SED: 0.34; VIG: 0.06; p =0.74), processing speed (Baseline to Post-test change: SED: 13.87; VIG: 13.32; p = 0.823), episodic memory (Baseline to Post-test change: SED: 28.98; VIG: 16.48; p = 0.569), or cognitive flexibility (Baseline to Post-test change: SED: 0.11; VIG: 0.02; p = 0.74). |
| Grieco et al. 2016; USA | N=320; 9.5 years | Clustered RCT  (duration: 10-15 minutes) | **Active lessons**  Students were randomly assigned to a condition by classroom (n= 20, 5 classes for each condition). Conditions were: (1) traditional sedentary lesson; (2) sedentary game (high interest control), (3) low to moderate-intensity physically active (LMPA) game, and (4) moderate to vigorous intensity physically active (MVPA) game. Each condition lasted between 10-15 minutes. | Time on task was measured with direct observations in 5 second intervals for each child. On-task behavior was defined as any behavior in which a student is attentive to the teacher or actively engaged in the appropriate task, as assigned by the teacher. Off-task behavior was defined as actions whereby a student was disengaged or distracted from the assigned task (i.e., behavior outside of the specifications of “on-task” behavior). A score was then calculated as the percent of time the child was rated as on-task. | **Active lessons**  Time on task decreased from baseline to post-intervention on the control lesson group (69.8 ±23.3 to 54.5 ±26.5, p < 0.001), and significantly increased in the same period for low/moderate physical activity game (70.4 ±24.3 to 80.7 ±23.9), p < 0.01), and for MVPA game (56.2 ±23.6 to 82.7 ±19.6, p < 0.01). No significant changes were observed between Control game between pre- and post-intervention.  A significant intervention effect was observed for MVPA game compared to control game and control lesson, and between LMPA game and control lesson, favoring the physical activity groups. A significant effect was also observed between control game and control lesson, favoring control game. No differences were observed between MVPA game and low/moderate physical activity game. |
| Norris et al. 2015; England | N=85; 9.5 years | Clustered RCT  (duration: 30 minutes) | **Active lessons**  Intervention: students participated in a 30-minute virtual field trip (VFT), supervised by their teacher and created on Google Earth. The intervention VFT was tailored to promote physical activity, and was themed after the 2012 London Olympics, including activities such as running as an analogue of the Olympic Stadium or flapping their arms when ‘flying’ to the next location.  Control: students participated in the same building-specific VFT, but instead of promoting physical activity, participants were seated throughout the session and completed no related activities. | A ten-item quiz was given to students with contents of the field trip being tested. | **Active lessons**  No difference was observed in recall test scores between active and sedentary field trip groups. |
| Parrish et al. 2018; Australia | N=88; 14.71 years | Clustered RCT  (duration: 5 months) | **Standing desk**  Intervention: comprised four components: new furniture (designed to reduce sitting), standing strategies (e.g., 30 min/day at standing desks), structured learning modules (supporting behavior change) and optional standing strategies (e.g., standing assemblies, outdoor lessons).  Control: schools continued with their usual program. | Non-verbal reasoning was assessed using the Matrix Reasoning subtest of the WISC-1V, which asks participants to complete abstract patterns by selecting which image, from a range of options, completes the pattern.  Working memory (or mental-attentional) capacity was assessed by the Figural Intersections Task, which requires participants to find the area of common intersection amongst an array of overlapping shapes. | **Standing desk**  A significant intervention effect was observed for working memory, favoring the intervention group (Difference: -0.42 (±1.37), p = 0.048).  No significant effect was observed for non-verbal reasoning (Difference: 0.39 ±5.59), p = 0.53) |
| Pitchford et al. 2019, Exp2; Malawi | N=608; 7.7 years | Clustered RCT  (duration: 3 to 6 months) | **Screen Time**  Intervention: Seven intervention schools used an app to support teaching mathematics for three to six months. Each child received several 30-min sessions with the interactive apps. The total mean time of interaction with the apps across the sample of intervention children was 8.9h (SD = 6.4), which is approximately eighteen 30-min sessions per child across the 14-month assessment period.  Control: Students had normal classes, without using the math app. | Mathematics attainment was assessed using the Early Grade Mathematics Assessment. The test was administered using a standardized procedure, including subtests assessing number identification, discrimination between two quantities, completing a pattern of numbers, solving word problems, and solving additions and subtractions. | **Screen Time**  A significant intervention effect was observed favoring the math app intervention group for math attainment scores compared to the control (F-score(1, 252): 9.34, p = 0.002). |
| Pitchford et al. 2019, Exp3; Malawi | N=360; 7.7 years | Clustered RCT  (duration: 14 weeks) | **Screen Time**  Experiment 3: During the intervention period, children assigned to the intervention group received instruction with the new reading app for 60 min a day (maximum possible time with app = 70h). For all 180 intervention children in each school to attend daily sessions in the learning centre, intervention children missed some standard class-based lessons, including teacher-led reading instruction, as well as other subjects, because of timetabling and hardware constraints.  Control: Students had normal classes, without using the reading app. | Reading was assessed using the Early Grade Reading Assessment. The test was administered using a standardized procedure, including subtests assessing letter naming, syllable segmentation, knowledge of initial sounds, syllable reading, familiar word reading, nonword reading, fluency in reading a written passage, reading comprehension, and listening comprehension. | **Screen Time**  A positive significant intervention effect was observed, with the intervention group increasing reading attainment scores compared to the control group after 14 weeks (F(1,316): 12.36, p < 0.001). No significant interactions were observed for gender (F(1, 316): 0.31, p = 0.576). |
| Resaland et al. 2016; Norway | N=1129; 10.2 years | Clustered RCT  (duration: 7 months) | **Additional PA**  Intervention: the intervention consisted of three components aimed at providing children with the opportunity to engage in additional 165 min of PA/week: i) physically active lessons for 90 min/week, conducted in the playground; physically active educational lessons were delivered in three core subjects – Norwegian (30 min/week), mathematics (30 min/week) and English (30 min/week); ii) PA physical activity breaks (5 min/day) implemented in the classroom during academic lessons; and iii) PA homework (10 min/day) prepared by teachers. In addition, pupils attending intervention schools participated in the curriculum-prescribed 90 min/week of PE and the curriculum-prescribed 45 min/week of PA. Thus, PA (165 min/week) and PE/PA (135 min/week) components provided children opportunities to engage in school-based physical activities 300min/week. The intervention was established as part of the mandatory school curriculum for all pupils attending intervention schools.  Control: Control schools were asked to provide the “normal practice” school curriculum, including usual amounts of PA/PE, being approximately 135 min/week. | Academic performance was measured for numeracy, reading, and English language, using standardized Norwegian national tests designed and administered by The Norwegian Directorate for Education and Training. A composite score of academic performance was also calculated using the measures of all subjects. | **Additional PA**  No significant intervention effects were observed for English (Mean difference: 0.48, 95%CI: -0.79, 1.76), Reading (Mean difference: -0.61, 95%CI: -1.90, 0.69), Numeracy (Mean difference: 0.30, 95%CI: -0.96, 1.56), or the composite score of academic performance (Mean difference: 0.01, 95%CI: -0.10, 0.13). |
| Hallstedt and Ghaderi 2018; Sweden | N=94; Grade 3 | Cross-Over Trial  (duration: 45 minutes, with 3-7 day wash out) | **Screen Time**  Participants answered a test on paper and on tablet. | Participants answered the Heidelberger Rechen Test 1–4 (HRT), which is a speed-test developed in Germany, used between grades 1-5 to test basic Math skills. Classes were randomized and answered the HRT on paper and on tablet on different occasions. The test provides scores in the following scales: addition, subtraction, missing term, count amount, and speed. | **Screen Time**  Scores were higher on all scales when the HRT was answered on paper compared to tablet (p < 0.001). |
| Frangou et al. 2019; Finland | N=63 and 43; 10.5 and 16 years | Cross-Over Trial  (duration: unclear length, tested 1 week later) | **Screen Time**  Experiment 1: participants (10-11-year-olds) were read three stories (2: Weschler Memory Scale-Revised; 1: created for the study; 40 words long) and told to write down everything that was dictated to them. Participants wrote notes using three modalities (order randomized): handwriting, keyboarding on a laptop, and keyboarding on a touchscreen keyboard of a tablet. Story recollection was assessed after 1 week.  Experiment 2: participants (16-years-old) were read three stories (2: Weschler Memory Scale-Revised; 1: created for the study; 40 words long) and told to write down everything that was dictated to them. Participants wrote notes using three modalities (order randomized): handwriting, keyboarding on a laptop, and keyboarding on a touchscreen keyboard of a smartphone. Story recollection was assessed after 1 week. | After 1 week, children aged 10-11 years were individually asked to orally recall everything they remembered about the stories, in a free-recall condition with as much time as  needed. A 20-item list of details was used with one point  earned for each recalled detail (a maximum of 20 points).  Testing for 16-year-old children was identical, except a 25-item list was used to assess memory. | **Screen Time**  In experiment 1, no significant effects of writing mode were found for 10-year-olds after 7 days (F(2, 60) = .001, p = 0.99), but for 11-year-olds, handwriting was better recalled compared to touch screen typing (t(31) = 2.15, p < 0.05) and keyboard-based writing (t(31) = 3.32, p < 0.01).  In experiment 2, the recollection score was better with handwriting compared to laptop-keyboard typing (t(42) = 3.09, p <= 0.01), but not different compared to mobile phone-keyboard typing (t(42) = 1.76 p = 0.085)). |
| Hanghoj et al. 2018; Denmark | N=190; 11.48 years | Non-Randomized Intervention  (duration: 3 weeks) | **Screen Time**  At-risk students participated in an intervention using games (e.g., Torchlight II, Minecraft), and game-related assignments with analogue gamified tools to promote positive student behavior and classroom participation in Mathematics and Danish. | Mathematics and Danish performance were evaluated by the school teachers who were asked to rate students’ general performance on these subjects. | **Screen Time**  The intervention positively impacted performance in Danish during and post-intervention (ANOVA effect size = 0.01, p ≤ 0.01). No significant effects were observed for Mathematics (ANOVA effect size = 0.02, p = 0.07). |
| Sherry et al. 2020; England | N=49; 9.7 years | Non-Randomized Intervention  (duration: 8 months) | **Standing desk**  Intervention: pupils of a single class received a height adjustable sit-stand desk that allows the user to manually shift between sitting and standing.  Control: in another school, a single class functioned as a control group, continuing with traditional classroom furniture and normal lessons. | Executive function was assessed using a computer version of the Stroop test. In this test, participants must correctly select the font color of a target word, ignoring the actual target color spelled out. Reaction time was the key outcome.  Visual spatial working memory capacity was assessed using a computer-based version of the Corsi Block Tapping test. For this test, participants were presented with a 3 × 3 grid of squares and a sequence occurred by individual squares temporarily changing color in which the participant had to accurately repeat. | **Standing desk**  A significant difference was observed at 4 months, favoring the intervention group for reaction time, compared to the control group (Standardized coefficient: 133.67, 95%CI: 3.72, 263.62, p = 0.044). However, no difference was observed at 8 months (Standardized coefficient: 0.11, 95%CI: -0.64, 0.86, p = 0.769).  In relation to visual spatial working memory capacity, no differences were observed between groups at 4 (Standardized coefficient: -0.33, 95%CI: -1.08, 0.43, p = 0.398) or 8 months (Standardized coefficient: 37.37, 95%CI: -92.58,167.32, p = 0.573). |
| Wick et al. 2018; Switzerland | N=38; 10.8 years | Non-Randomized Intervention  (duration: 11 weeks) | **Standing desk**  Intervention: Standing desks were provided, and teachers were asked to encourage pupils to work for about 60 min a day at the standing desks. Pupils stood on an unstable surface while standing at the desk. The teacher reminded the children regularly to use the standing desks during the lessons, but the children were allowed to manage their standing time by themselves. Once a child was standing, the standing time needed to be maintained for at least 10 min.  Control: the control class attended their regular classes in the 11 weeks with no restrictions regarding the activity level of the lessons. | Cognitive function was assessed using several indicators.  In the forward digit span test, participants first had to repeat a series of numbers in order, followed by repeating a series of numbers in reverse order. The length of the sequence number was constantly increased by one number in the course of the experiment, if the subjects answered right at the first and/or the second trial. Only two wrong answers were allowed. The time window in which the subjects had to answer was not limited. The maximum attained sequence length on digit span backward and the sum of correct trials on digit span forward was analyzed.  In the backward digit span test, participants first had to repeat a series of numbers in order, followed by repeating a series of numbers in reverse order. The length of the sequence number was constantly increased by one number in the course of the experiment, if the subjects answered right at the first and/or the second trial. Only two wrong answers were allowed. The time window in which the subjects had to answer was not limited. The maximum attained sequence length on digit span backward and the sum of correct trials on digit span forward was analyzed.  Inhibitory control was assessed with the Eriksen flanker task test, which measures the ability to suppress distractors and attend relevant information. Participants were asked to respond as quickly and accurately as possible with a key press depending on the direction of a centrally presented arrow (left or right), which was showed always in the same place above a fixation point. The arrow was flanked by congruous (facing in the same direction) or incongruous (facing the opposite direction) arrows, which had to be ignored. After 10 test trials, participants completed three series of 100 experimental trials with equiprobable and random congruency and incongruency (each 150 trials). The fixation point was shown for 1000 ms and the arrows were presented for 200 ms with interstimulus intervals of 1000, 1250, 1500, 1750 and 2000 ms. Answers had to be given within 1000 ms. Measures of median reaction time (in ms) and accuracy (in%, range 0–1) were analyzed for congruous and incongruous stimuli. | **Standing desk**  No differences were observed between control and intervention groups for forward digit span number of trials (p = 0.59) or maximum sequence length (p=0.58), for backward digit span number of trials (p = 0.56) or maximum sequence length (p = 0.28), for reaction time with congruous (p = 0.92) or incongruous (p = 0.85) stimuli, or for accuracy with congruous (p = 0.52) or incongruous (p = 0.82) stimuli.  No significant time*group interactions were observed for any of the cognitive function outcomes (p > 0.05). |
| Aggio et al. 2016; England | N=8462; 7.0 years | Longitudinal  (Follow-up: 4 years) | **Homework**  Mothers reported how many minutes their child typically spent per week doing home-work, categorized into 3 equal groups for each domain of parent-reported physical activity and sedentary behavior (low, medium, and high). | Verbal reasoning and verbal knowledge measures with the British Abilities Scale Verbal Similarities. | **Homework**  After four years, high volume of homework was associated with higher verbal reasoning and verbal knowledge score, compared to low homework (Beta coefficient: 0.5, 95%CI: 0.0, 0.9). No differences were observed when comparing Low and Medium homework (Beta coefficient: 0.1, 95%CI: -0.4, 0.5). |
| Corder et al. 2015; England | N=845; 14.5 years | Longitudinal  (Follow-up: 1.5 years) | **Homework**  Participants reported daily time spent on homework for week and weekend days and a weighted mean of daily time was calculated. | Academic performance was measured with the scores of the  General Certificate of Secondary Education (GCSE) exams. This exam is taken at the end of Year 11 by the majority students at the end of compulsory education in England, Wales and Northern Ireland. In most schools at the time, it was compulsory to take GCSE in English, Mathematics, Sciences, and a foreign language with a choice of subjects such as History and Geography. | **Homework**  Time spent doing homework was positively associated with academic achievement (Unstandardized coefficient: 17.4, 95%CI: 5.6, 29.3, p = 0.007). |
| Esteban-Cornejo et al. 2015; Spain | N=1146; 12.1 years | Cross-Sectional | **Homework**  Participants were asked about how much time, on average, they spent doing homework/studying with a computer and doing homework/studying without a computer during the weekdays and weekend days separately. An average daily weighted mean was calculated based on the reports. | Academic performance was analyzed using the grades for language, math, math & language, and grade point average, assessed through school records at the end of the academic year. | **Homework**  No significant associations were observed between doing homework with a computer and grade point average (Standardized coefficient: -0.002, p = 0.943), language grade (Standardized coefficient: -0.019, p = 0.511), math grade (Standardized coefficient: 0.051, p = 0.065), and math & language grades (Standardized coefficient: 0.018, p = 0.527).  A positive association was observed between time spent doing homework without a computer and grade point average (Standardized coefficient: 0.112, p < 0.001), language grade (Standardized coefficient: 0.081, p = 0.003), math grade (Standardized coefficient: 0.058, p = 0.033), and math & language grade (Standardized coefficient: 0.075, p = 0.006). |
| Fernandez-Alonso et al 2017; Spain | N=26543; 14.4 years | Cross-Sectional | **Homework**  Participants reported how much time they spent on homework per day. The average time spent on homework by students of each school were used as an indicator of amount of homework assigned per school. | Academic achievement was assessed for four subjects: Spanish, Mathematics, Science, and Citizenship. The scores for each subject were calculated from a test, completed on paper. | **Homework**  At the student level, negative associations were observed between student homework time and academic achievement related to Citizenship (Standardized coefficient: -0.055, SE: 0.007), Sciences (Standardized coefficient: -0.053, SE: 0.006), Spanish (Standardized coefficient: -0.055, SE: 0.006), and Mathematics (Standardized coefficient: -0.050, SE: 0.008).  However, at the school level, homework assigned by the school were positively related to academic achievement in Citizenship (Standardized coefficient: 0.083, SE: 0.011), Sciences (Standardized coefficient: 0.075, SE: 0.009), Spanish (Standardized coefficient: 0.068, SE: 0.011), and Mathematics (Standardized coefficient: 0.046, SE: 0.011). |
| Huang, Zeng, and Ye 2019; China | N=1164.0; 14.9 years | Cross-Sectional | **Homework**  Participants were asked about how much time, on average, they spent doing homework/studying with a computer and doing homework/studying without a computer during the weekdays and weekend days separately. An average daily weighted mean was calculated based on the reports. | Academic performance was evaluated based on four courses, including Chinese, mathematics, English, and science, which were also the official final examinations at the end of 2015 autumn semester. Each course (original test score) was T-scored and summed up for the final values of academic performance. The tests of all courses were conducted by the registered relevant academic teachers. | **Homework**  No associations were observed between study-based sedentary behavior on weekends and academic performance for boys (Standardized coefficient: 0.33, SE: 0.57) and between study-based sedentary behavior on weekdays for girls (Standardized coefficient: 0.50, SE: 0.26).  Time spent on study-based sedentary behaviors (weighted) was positively associated with academic performance for boys (Standardized coefficient: 0.77, SE: 0.22), and girls (Standardized coefficient 0.67, SE: 0.20). Positive associations were also observed between study-based sedentary behaviors on weekdays and academic performance for boys (Standardized coefficient: 0.99, SE: 0.32), and study-based sedentary behaviors on weekends and academic performance for girls (Standardized coefficient: 1.23, SE: 0.60). |
| Hunter, Leatherdale, and Carson 2018; Canada | N=4408; 14.7 years | Longitudinal | **Homework**  Students were asked to report the average amount of time per day they spent doing homework. Values were categorized into quartiles for each year: Year 1: Q1: <60 minutes/day, Q2: 61-90 minutes, Q3: 90-120 minutes, Q4: >121 minutes/day; Year 2: Q1: <60 minutes/day, Q2: 61-90 minutes, Q3: 90-150 minutes, Q4: >151 minutes/day; Year 3: Q1: <60 minutes/day, Q2: 61-90 minutes, Q3: 90-180 minutes, Q4: >181 minutes/day. | Academic achievement was measured for Mathematics and English by one item where participants reported their overall mark on each subject (ranging from less than 50% to 90-100%, and dichotomized using the 80% cutoff). | **Homework**  A positive association was observed between the fourth quartile of doing homework (those who spent most time on homework, >121/151/181 min/day),compared to Q1 of homework (<60 minutes/day at baseline) and English achievement (>80% marks, OR: 1.16, 95%CI: 1.02, 1.31).  No significant associations were observed between doing homework and Math achievement. And no differences were observed between quartiles 2 and 3 compared to quartile 1 of doing homework for English achievement. |
| Ishihara et al. 2018; Japan | N=325; 12 to 13 years | Cross-Sectional | **Homework**  Learning duration was assessed using the time spent on learning after school on weekdays and weekends, and the use of a cram school or private teacher. | Academic achievement was assessed using the total grade points of eight school subjects, including Japanese, Mathematics, Social Studies, Sciences, English, Music, Arts, and Home Economics/Vocational Technology, as evaluated at the end of the year by the participants' schoolteachers. | **Homework**  A positive direct association between learning duration and academic achievement was observed (Unstandardized coefficient of SEM: 0.18, SE: 0.05, p ≤ 0.001; Unstandardized coefficient of regression model: 0.15, SE: 0.05, p ≤ 0.001). This association was confirmed for girls (Unstandardized coefficient of SEM: 0.15, SE: 0.07, p ≤ 0.001) and boys (Unstandardized coefficient of SEM: 0.18, SE: 0.07, p ≤ 0.001). |
| Peiro-Velert et al. 2014; Spain | N=3095.0; 12 to 18 years | Cross-Sectional | **Homework**  Participants were asked about how much time, on average, they spent doing homework/studying with a computer. | Academic achievement or performance was assessed using the number of subjects each participant failed in the previous academic year. | **Homework**  A negative association was observed between time spent using a computer for homework and academic achievement (Spearman's correlation: -0.37, 95%CI: -0.48, -0.25). |
| Rajala et al. 2019; Finland | N=420; 13.7 years | Cross-Sectional | **Stationary time/sedentary time**  Stationary time was measured using accelerometers, positioned at the right side of the hip. Whole-day and school-time stationary time were calculated. | School performance was assessed using the following question: “How would you rate your performance in the following subjects compared to your peers?” Participants reported their performance in the following subjects: mother tongue, English, other foreign languages, mathematics, biology/geography, physics/chemistry, religion/ethics, history, music, visual arts, crafts, and physical education. | **Stationary time/sedentary time**  A positive significant association was observed between school-time stationary time and school performance (unstandardized coefficient: 0.16, SE: 0.06, p < 0.05). |
| Rasberry et al. 2017; USA | N=15624; grades 9-12 | Cross-Sectional | **Recess/PE**  Physical Education classes attendance was reported by the participants, if they had to attend one or more days in an average week when they were in school. | Academic achievement was measured with a question on self-reported grades in school: “During the past 12 months, how would you describe your grades in school?” Students could select one of the following response options: mostly A’s, mostly B’s, mostly C’s, mostly D’s, mostly F’s, none of these grades, and not sure. | **Recess/PE**  No significant associations were observed between attending physical education classes and academic achievement. |
| Sanders et al. 2019; Australia | N=4013; 10 to 15 years | Cross-Sectional | **Homework**  Participants filled time-use diaries, and were interviewed in the following day to confirm the reports and to add more information. Screen time related to educational activities, such as homework were among the screen time activity categories. | School achievement was assessed using the records of the National Assessment Program - Literacy and Numeracy data, which are standardized tests. Numeracy was measured using a single test and literacy was measured using four tests covering reading, writing, spelling, and grammar. Principal component analysis on the four literacy scores formed a single factor score to represent literacy. | **Homework**  A positive association between education/homework screen time and school achievement was observed (β = 0.06, 95%CI: 0.029, 0.092). |
| Tang and Patrick 2018; USA | N=40389; grade 8 and grade 10 | Cross-Sectional | **Homework**  Participants were asked about how much time, on average, they spent doing homework/studying with a computer and doing homework/studying without a computer. | Academic achievement was assessed using the school grades reported by the participants. | **Homework**  A positive association between homework time and odds of high grades was observed (Odds Ratio: 1.27, SE: 0.03, p < 0.001).  Time using a computer for school was also positively associated with higher odds of high grades (Odds Ratio: 1.16, SE: 0.03, p < 0.001). |
| Wang et al. 2020; China | N=176428; 12.11 years | Cross-Sectional | **Homework**  Weekday time spent on math homework was reported by students with options of no homework, 1–15 min, 16–30 min, 31–60 min, 1–2 h, and above 2 h. | Mathematics achievement was assesses using a test developed by mathematics teachers and experts in mathematics education and educational measurement. The test involved three content strands including numbers & algebra, space & shape, and statistics & uncertainty. | **Homework**  A significant positive correlation was observed between math homework time on weekdays and math achievement (r = 0.182). |
| Yamada et al. 2019; Japan | N=1663; 9.5 years | Cross-Sectional | **Homework**  Participants reported in a questionnaire if they regularly attended Cram School, and how much time they spent studying at home. | Academic performance was assessed by asking the participants if they understand school lessons well. | **Homework**  No significant associations were observed between Cram school attendance and low academic performance for the whole sample (OR: 1.01, 95%CI: 0.73, 1.40), or when analyses were stratified for Senior grade (OR: 1.03, 95%CI: 0.66, 1.61) and Junior grade (OR: 1.01, 95%CI: 0.62, 1.66).  Positive associations were observed between studying for <1h/day (compared to ≥1h/day) and odds of low academic performance for the whole sample (OR: 1.82, 95%CI: 1.34, 2.49), or when analyses were stratified for Senior grade (OR: 1.92, 95%CI: 1.29, 2.85) and Junior grade (OR: 1.81, 95%CI: 1.08, 3.02). |

| **Supplementary Table 4: Musculoskeletal Growth** | | | | | |
| --- | --- | --- | --- | --- | --- |
| **Study** | **Sample** | **Study Design** | **Exposure** | **Outcome** | **Main Findings** |
| Cronholm et al. 2018; Sweden | N=228; Mean age: 7.7 years | Non-Randomized Intervention (duration: 7 years) | **Recess/Physical Education**  Intervention: Increased level of physical education from 60 minutes a week to 200 minutes a week, provided as daily 40-minute classes.  Control: Continued 60 minutes/week of physical education classes. | Objectively measured **height** and **weight**. | **Recess/PE**  No difference between intervention and control groups for boys height (Baseline: Intervention: 128.6 ± 6.8, Control:130.8 ± 5.9; Follow-up: Intervention: 172.8 ± 9.2, Control: 173.4 ± 9.2), boys weight (Baseline: Intervention: 27.7 ± 5.4, Control: 28.6 ± 4.9; Follow-up: Intervention: 61.4 ± 11.8, Control: 61.3 ± 11.5), girls height (Baseline: Intervention: 127.6 ± 5.9, Control:129.2 ± 7.7; Follow-up: Intervention: 166.3 ± 6.1, Control: 164.5 ± 7.5), or girls weight (Baseline: Intervention: 27.3 ± 5.4, Control: 27.3 ± 5.2; Follow-up: Intervention: 58.5 ± 11.0, Control: 54.3 ± 10.6) at 7 years. |
| Muller et al 2016; Germany | N=236; Mean age: 11.5 years | Clustered RCT (duration: 4 years) | **Recess/Physical Education**  Intervention: Participants in the intervention group received additional physical exercise daily, lasting 45 minutes, with at least 15 minutes of endurance training.  Control: Continued to receive normal classes, with two PE classes/week. | **Fat-free mass** was measured using bioelectrical impedance. | **Recess/Physical Education**  No difference between intervention and control for fat-free mass (Group difference: -0.81, 95%CI: -3.3, 1.6, p= 0.483) at 4 years. |
| Xue et al. 2016; China | N=1586; Mean age: 10.0 years | Cross-Sectional | **Homework**  The daily frequency and duration of homework time for weekdays and weekends were self-reported for children ≥ 9 years and parent-reported for children < 9 years, through in-person interviews. Homework was defined as work assigned by the teacher and completed on paper (including work assigned by the teacher for children to do in the school). Mean daily homework time was calculated based on weekend and weekday estimates, and categorized into tertiles (0.5 hours/day, 0.8 hours/day, and 1.8 hours/day) for analysis. | **Fat-free mass index** was calculated using objectively measured skinfold measurements, height, and weight. Weight and standing height were measured in duplicate to the nearest 0.1 kg and 0.1 cm, respectively. Skin-fold thicknesses were measured in duplicate on the right side of the body to the nearest 0.1 mm using a caliper (Holtain Ltd., Crosswell, UK). Percent body fat was calculated using the equations of Slaughter et al. with fat-free mass index = (weight x (1 - %BF))/height^2^. | **Homework**  Positive association between homework time (tertiles) and fat-free mass index, for girls (Least square means: Tertile 1 (T1): 13.6, 95%CI: 13.5, 13.8; T2: 13.9, 95%CI: 13.7, 14.0; T3: 14.0, 95%CI: 13.9, 14.1; p for trend 0.03).  No association between homework time (tertiles) and fat-free mass index, for boys (Least square means: Tertile 1 (T1): 14.5, 95%CI: 14.4, 14.7; T2: 14.7, 95%CI: 14.6, 14.9; T3: 14.6, 95%CI: 14.5, 14.7; p for trend = 0.5). |

| **Supplementary Table 5: Risks (Injury)/Harm** | | | | | |
| --- | --- | --- | --- | --- | --- |
| **Study** | **Sample** | **Study Design** | **Exposure** | **Outcome** | **Main Findings** |
| Contardo Ayala et al. 2016; Australia | N=41; age range: 11-12 years (mean age: intervention= 11.5 years, control=11.7 years) | Non-Randomized Intervention (follow-up: 8 months) | **Standing desk**  Intervention: Each participant in the intervention class was provided with a manually adjustable height-adjustable workstation (LearnFit, Ergotron Pty Ltd., Sydney, Australia).  Control: The control class followed standard pedagogical practice and retained traditional classroom furniture. | **Back musculoskeletal pain/discomfort, Lower limbs musculoskeletal pain/discomfort, and Upper limb musculoskeletal pain/discomfort** during last month assessed via the modified Nordic Musculoskeletal Questionnaire (self-report). | **Standing desk**  The intervention was not associated with odds of back musculoskeletal pain (OR= 1.48; 95% CI: 0.38, 5.72; p= 0.567), odds of lower limb pain (OR= 1.24; 95% CI: 0.30, 5.10; p= 0.765), odds of upper limb pain (OR= 0.79; 95% CI: 0.18, 3.47; p= 0.758) at 8 months. |
| Contardo Ayala et al. 2018; Australia | N=88; mean age: 14.8 years | Non-Randomized Intervention (follow-up: 17 weeks) | **Standing desk** Intervention: Traditional classroom furniture in the intervention classroom was replaced with height-adjustable desks (LearnFit, Ergotron Inc., Minnesota, USA) and lab stools (Furnware Bodyfurn Lab stool, New Zealand) for every student and the teacher.  Control: Students and teachers followed standard pedagogical practice in classroom using traditional ‘seated’ furniture. | **Back musculoskeletal pain/discomfort, Lower limbs musculoskeletal pain/discomfort, and Upper limb musculoskeletal pain/discomfort** during last month assessed via the modified Nordic Musculoskeletal Questionnaire (self-report). | **Standing desk**  There were no significant differences between control and intervention for back pain  (OR= 1; 95% CI: 0.34, 2.90), for lower limb pain (OR= 1.94; 95% CI: 0.62, 6.04), and for upper limb pain (OR= 0.76; 95% CI: 0.26, 2.21) at 17 weeks. |
| Sherry et al. 2020; England | N=49; mean age: 9.7 years | Non-Randomized Intervention (follow-up: 8 months) | **Standing desk**  Intervention: All pupils within a single class received a height adjustable sit-stand desk that allows the user to manually shift between sitting and standing.  Control: Within the other school, a single class functioned as a control group, continuing with traditional classroom furniture and normal lessons. | **Musculoskeletal discomfort (whole body, upper limb, neck and back, and lower limb**) assessed via self-report questionnaire. | **Standing desk**  No intervention effects were observed for whole body musculoskeletal discomfort (β= ‑0.27; 95% CI: -0.62,0.08; p= 0.132), for upper limb musculoskeletal discomfort (β= ‑0.38; 95% CI: -1.10,0.35; p= 0.31), for neck and back musculoskeletal discomfort  (β = -0.79; 95% CI: -1.72,0.15; p= 0.099), for lower limb musculoskeletal discomfort (β= ‑0.39; 95% CI: -1.85,1.07; p= 0.603) at 4 months.  No intervention effects were observed for whole body musculoskeletal discomfort  (β= -0.07; 95% CI: ‑0.42, 0.29; p= 0.71), for upper limb musculoskeletal discomfort (β= ‑0.08; 95% CI: -0.81,0.64; p= 0.818), for neck and back musculoskeletal discomfort  (β = -0.09; 95% CI: -1.02,0.84; p= 0.851), for lower limb musculoskeletal discomfort  (β= 0.08; 95% CI: -1.36,1.53; p= 0.911) at 8 months. |
| Ee et al. 2018; Australia | N=47; age range: 10-11 years | Cross-Over Trial (Duration: 21 days/condition; total 42 days) | **Standing desk**  In both 2016 and 2017, the class was divided into two groups—one group used the standing desks while the other group used the traditional seated desks. After 21 school days (three rotations of the school’s seven-day timetable), the students changed desks. Students who had used the traditional seated desks previously started using the standing desks and vice versa. Students remained in this condition for a further 21 school days. | **Musculoskeletal discomfort at nine body parts (neck, shoulders, elbows, wrists/hands, upper back, lower back, hips/ thighs, knees, and ankles/ feet)** assessed via the modified version of the Nordic Musculoskeletal Questionnaire (self-report). | **Standing desk**  Standing condition was associated with less neck pain, compared to the sitting condition  (B= -0.28; SE: 0.1; p= 0.005)  Compared to sitting condition, standing condition was not associated with ankle/feet pain (B= -0.179; SE: 0.123; p= 0.145), lower back pain (B= -0.111; SE: 0.103; p= 0.281), elbow pain (B= -0.092; SE: 0.106; p= 0.384), hip/thigh pain (B= -0.177; SE: 0.097; p= 0.07), knee pain (B= -0.172; SE: 0.121; p= 0.156), shoulder pain (B= -0.15; SE: 0.102; p= 0.142), upper back pain (B= -0.132; SE: 0.10; p= 0.189), wrist/hand pain (B= -0.084; SE: 0.104; p= 0.419).  Compared to sitting condition, standing condition was associated with lower odds of any elbow pain (OR= 0.74; 95% CI: 0.57, 0.96; p= 0.023), lower odds of any lower back pain (OR= 0.71; 95% CI: 0.55, 0.91; p= 0.007), lower odds of any neck pain  (OR= 0.52; 95% CI: 0.41, 0.67; p< 0.001), and lower odds of any shoulder pain  (OR= 0.54; 95% CI: 0.42, 0.70; P< 0.001).  Compared to sitting condition, standing condition was not associated with odds of any ankle pain (OR= 1.12; 95% CI: 0.88, 1.44; p= 0.35), odds of any hip/thigh pain (OR= 0.79; 95% CI: 0.62, 1.02; p= 0.07), odds of any knee pain (OR= 0.8; 95% CI: 0.62, 1.03; p= 0.079), odds of any upper back pain (OR= 0.81; 95% CI: 0.63, 1.04; p= 0.103), odds of any wrist/hands pain (OR= 0.8; 95% CI: 0.62, 1.03; p= 0.089). |
| Ku et al. 2019; Taiwan | N=1958; age range: 7-12 years | Cross-Sectional & Longitudinal (follow-up: 4 years) | **Homework**  Homework time assessed via self-report questionnaire. | **Myopia**  According to the Implementation Regulations Governing Health Examinations to Elementary and Junior High School Students in Taiwan, the annual vision screening was conducted in the school. Parent whose child (aged ≥ 7 years) failed the vision screening were required by the school to take their child to a local eye clinic for regular eye examinations. It is compulsory that documentation of the examination signed by an ophthalmologist is submitted to the school health center. | **Homework**  Compared to < 0.5 hours/day, ≥ 2 hours/day of cram school attendance was associated with higher odds of prevalent myopia  (OR= 1.65; 95% CI: 1.24, 2.19; p< 0.001), but 0.5-1.9 hours/day of cram school attendance was not associated with odds of prevalent myopia (OR= 1.28; 95% CI: 0.87, 1.90; p= 0.214).  Compared to < 0.5 hours/day, ≥ 2 hours/day of cram school attendance was associated with higher risk of incident myopia  (RR= 1.31; 95% CI: 1.03, 1.68; p= 0.03), but  0.5-1.9 hours/day of cram school attendance was not associated with risk of incident myopia (RR= 1.19; 95% CI: 0.85, 1.67; p= 0.302).  Cram school attendance was positively associated with incidence of myopia (<0.5 hours/day: 23.8%, 0.5-1.9 hours/day: 30.5%, ≥2.0 hours/day: 30.2%; p= 0.029), prevalence of myopia (<0.5 hours/day: 15.9%, 0.5-1.9 hours/day: 27.8%, ≥2.0 hours/day: 34.5%;  p< 0.001) |
| Dianat, Alipour, and Jafarabadi 2018; Iran | N=1611; mean age: 13.4 years | Cross-Sectional | **Homework**  Homework time assessed via self-report questionnaire. | **Neck pain and shoulder pain** assessed via self-report questionnaire. | **Homework**  Compared to just right homework group, Too Much homework group was positively associated with odds of shoulder pain  (OR= 1.45; 95% CI: 1.03, 2.03; p= 0.032), but not associated with neck pain (OR= 1.29; 95% CI: 1.00, 1.66; p= 0.052).  Compared to just right homework group, Not Enough homework group was not associated with shoulder pain (OR= 1.39; 95% CI: 0.90, 2.14; p= 0.141), and neck pain (OR= 1.22; 95% CI: 0.91, 1.64; p= 0.134). |
| Gelfand et al. 2020; USA | N=1012; mean age: 16.5 years | Cross-Sectional | **Homework**  Homework time assessed via self-report questionnaire. | **Headache frequency** assessed via self-report questionnaire. | **Homework**  There was a positive association between hours of homework on school nights and headache frequency (β= 0.7; 95% CI: 0.4, 1.0). |
| Gheysvandi et al. 2019; Iran | N=693; mean age: 9.7 years | Cross-Sectional | **Homework**  Homework time assessed via self-report questionnaire. | **Neck pain and shoulder pain** assessed via self-report questionnaire with the pre-shaded manikin pictures in order to show the desired areas. | **Homework**  Compared to just right amount of homework group, too much homework group had higher odds of neck pain (OR= 2.59; 95% CI: 1.49, 4.51; p= 0.001), and higher odds of shoulder pain (OR= 1.94; 95% CI: 1.10, 3.42; p= 0.022).  There were no differences in odds of neck pain (OR= 0.89; 95% CI: 0.46, 1.72; p= 0.74) and in odds of shoulder pain (OR= 0.82; 95% CI: 0.42, 1.62; p= 0.579) between just right and not enough homework. |
| Guan et al. 2019; China | N=19934; mean age: 10.6 years | Cross-Sectional | **Homework**  Studying time assessed via self-report questionnaire. | **Myopia and Visual Acuity** assessed by a nurse and staff assistant in each school, who had been trained by optometrists. | **Homework**  There was a positive relationship between myopia and study times of >60 minutes, compared to 0 minutes (β= 0.318; 95% CI: 0.070, 0.566; p= 0.012).  There were no differences in myopia when comparing study times of 0 minutes to 1-30 minutes (β= 0.101; 95% CI: -0.148, 0.349;  p= 0.428), and to 31-60 minutes (β= 0.176; 95% CI: -0.071, 0.423; p= 0.163).  There were no differences in visual acuity when comparing study times of 0 minutes to 1-30 minutes (β= -0.003; 95% CI: -0.022, 0.016; p= 0.738), to 31-60 minutes (β= ‑0.002; 95% CI: -0.020, 0.017; p= 0.866), and to >60 minutes (β= -0.016; 95% CI: ‑0.036, 0.003; p= 0.099). |
| Harris et al. 2015; Australia | N=1351; mean age: 13.2 years | Cross-Sectional | **Screen Time**  School computer use assessed via self-report questionnaire. | **Musculoskeletal soreness** assessed via self-report questionnaire. | **Screen Time**  There was a direct positive relationship between school computer exposure and musculoskeletal soreness (β= 0.163;  p< 0.001). |
| Martinez-Lopez et al. 2015; Spain | N=2293; mean age: 14.18 years | Cross-Sectional | **Homework**  Homework time assessed via self-report questionnaire. | **Self-perceived health, pain, and well-being** assessed via the self-report Health Behaviour in School-Aged Children (HBSC) questionnaire. | **Homework**  There was positive association between homework on weekdays and odds of sometimes having pain (compared to never) for boys (OR= 1.206; 95% CI: 1.006, 1.445; p= 0.043), but no association for girls  (OR= 0.917; 95% CI: 0.747, 1.127; p= 0.411).  Homework on weekdays was not associated with odds of low self-perceived health (compared to excellent) for boys  (OR= 11.012; 95% CI: 0.834, 1.227;  p= 0.907) and for girls (OR= 1.025; 95% CI: 0.819, 1.284; p= 0.83), odds of sometimes having well-being (compared to always) for boys (OR= 1.246; 95% CI: 0.937, 1.657;  p= 0.13) and for girls (OR= 0.891; 95% CI: 0.641, 1.238; p=0.492).  Homework on weekends was not associated with odds of sometimes having pain (compared to never) for boys (OR= 1.069; 95% CI: 0.890, 1.284; p= 0.475) and  for girls (OR= 0.936; 95% CI: 0.762, 1.148; p= 0.525), odds of low self-perceived health (compared to excellent) for boys (OR= 0.942; 95% CI: 0.775, 1.145; p= 0.55) and for girls (OR= 1.079; 95% CI: 0.861, 1.353; p= 0.51),  and odds of sometimes having well-being (compared to always) for boys (OR= 1.147; 95% CI: 0.859, 1.531; p= 0.354) and for girls (OR= 0.788; 95% CI: 0.572, 1.085; p= 0.144) |
| Sanders et al. 2019; Australia | N=4013; age range: 10-15 years | Cross-Sectional | **Homework**  Educational screen time measured using time-use diaries administered to the child and a face-to-face interview on the day following the diary. | **Global health** assessed via proxy-report questionnaire. | **Homework**  There was no association between educational screen time and global health (β= -0.005; 95% CI: -0.025, 0.014). |
| Shan et al. 2014; China | N=2842; age range: 15-19 years | Cross-Sectional | **Recess/PE**  Recess time assessed via self-report questionnaire. | **Neck and shoulder pain** assessed via self-report questionnaire. | **Recess/PE**  Compared to sufficient recess, there were higher neck or shoulder pain rates for those with insufficient class recess, (Sufficient: 33.7%, Insufficient: 45.04%; p< 0.001).  There was a positive association between insufficient class recess and odds of neck or shoulder pain (OR= 1.34; 95% CI: 1.12, 1.59). |
| Singh et al. 2019; India | N=1234; mean age: 10.5 years | Cross-Sectional | **Homework**  Studying time obtained using a questionnaire. The parents and the children jointly estimated studying time during the oral questionnaire in increments of 0.5 hours. | **Myopia**  Children were screened on site by two optometrists. All the children who were unable to read 6/9 letter size in either eye, were wearing spectacles, or complained of blurred vision with the existing spectacles were referred to the hospital for a detailed eye examination. Myopia was assessed via a detailed ophthalmic examination at the hospital. | **Homework**  There was a positive association between studying >4-8 hours, compared to 0-4 hours, and odds of myopia (OR= 2.94; 95% CI: 1.32, 6.57; p= 0.008). |
| Wang et al. 2020; China | N=176428; mean age: 12.11 years | Cross-Sectional | **Homework**  The homework time spent on math assessed via self-report questionnaire. | **Visual acuity** Standard logarithmic visual acuity chart with a lightbox was used to examine students’ visual acuity. | **Homework**  There was a negative association between math homework on the weekday and visual acuity (β= -0.024; t-score= -3.78). |
| Xu et al. 2014; China | N=815; mean age: 14.1 years | Cross-Sectional | **Homework**  Homework time was assessed via self-report questionnaire. | **Best Worst Scaling method for Health Related Quality of Life** and **Standard Gamble method for Health Related Quality of Life** assessed via the self-report Child Health Utility 9D (CHU9D)-Chinese version. | **Homework**  Homework time was negatively associated with best worst scaling method CHUD9D utility scores (Adjusted mean difference= ‑0.021; SE: 0.005; p< 0.01) and standard gambling method CHUD9D utility scores (Adjusted mean difference= -0.019; SE: 0.004; p< 0.01). |
| Yu et al. 2020; China | N=253301; Grade 1-12. | Cross-Sectional | **Homework**  Homework time assessed via questionnaire. Children and parents jointly filled out the questionnaire on the Internet according to their own situation and submitted the questionnaire directly online. | **Visual acuity, Light visual impairment, Mild visual impairment, and Severe visual impairment**  The students’ uncorrected visual acuity (UCVA) was examined in all schools by trained optometrists by the same standard logarithmic visual acuity charts (Chinese standard for logarithmic visual acuity charts, GB11533-2011) on a light box with 300 to 500 lux illumination, following regular procedures. | **Homework**  Compared to ≤ 1 hour/day, those with  > 3 hours/day of homework have a higher prevalence of light visual impairment (Prevalence: > 3 hours/day: 7.49%,  ≤ 1 hour/day: 6.13%; 95% CI: > 3 hours/day (7.15, 7.83), ≤ 1 hour/day: (5.92, 6.34);  p< 0.001), a higher prevalence of mild visual impairment (Prevalence: > 3 hours/day: 12.6%, ≤ 1 hour/day: 7.93%; 95% CI:  > 3 hours/day: (12.2, 13.1), ≤ 1 hour/day: (7.70, 8.17); p< 0.001), a higher prevalence of severe visual impairment (Prevalence:  > 3 hours/day: 3.96%, ≤ 1 hour/day: 1.00%; 95% CI: > 3 hours/day: (3.70, 4.21),  ≤ 1 hour/day: (0.91, 1.08); p< 0.001),  a positive association with the odds of UCVA <6/18 (OR= 1.1; 95% CI: 1.03, 1.17;  p= 0.004), but no association with odds of UCVA <6/12 (OR= 1.05; 95% CI: 0.99, 1.11; p= 0.092).  Compared to ≤ 1 hour/day, those with  2-3 hours/day of homework have no difference in prevalence of light visual impairment (Prevalence: 2-3 hours/day: 6.38%, ≤ 1 hour/day: 6.13%; 95%CI:  2-3 hours/day: (6.17, 6.60), ≤ 1 hour/day: (5.92, 6.34)), a higher prevalence of mild visual impairment (Prevalence: 2-3 hours/day: 8.87%, ≤ 1 hour/day: 7.93%; 95% CI:  2-3 hours/day: (8.62, 9.12), ≤ 1 hour/day: (7.70, 8.17); p< 0.001), a higher prevalence of severe visual impairment (Prevalence:  2-3 hours/day: 1.76%, ≤ 1 hour/day: 1.00%; 95% CI: 2-3 hours/day: (1.64, 1.87),  ≤ 1 hour/day: (0.91, 1.08); p< 0.001),  a positive association with the odds of UCVA <6/18 (OR= 1.07; 95% CI: 1.01, 1.13;  p= 0.026), but no association with the odds of UCVA <6/12 (OR= 1.05; 95% CI: 1.00, 1.10; p= 0.059).  Compared to ≤ 1 hour/day, those with 1-2 hours/day of homework have a lower prevalence of light visual impairment (Prevalence: 1-2 hours/day: 5.42%,  ≤ 1 hour/day: 6.13%; 95% CI: 1-2 hours/day: (5.25, 5.59), ≤ 1 hour/day: (5.92, 6.34);  p< 0.001), a lower prevalence of mild visual impairment (Prevalence: 1-2 hours/day: 6.16%, ≤ 1 hour/day: 7.93%; 95% CI:  1-2 hours/day: (5.97, 6.34), ≤ 1 hour/day: (7.70, 8.17); p< 0.001), a lower prevalence of severe visual impairment (Prevalence:  1-2 hours/day: 0.81%, ≤ 1 hour/day: 1.00%; 95% CI: 1-2 hours/day: (0.75, 0.88),  ≤ 1 hour/day: (0.91, 1.08); p< 0.001), but no association with the odds of UCVA <6/18(OR= 0.97; 95% CI: 0.91, 1.03;  p= 0.287), and no association with the odds of UCVA <6/12 (OR= 1; 95% CI: 0.95, 1.05;  p= 0.891). |
| Zhang et al. 2015; China | N=3000; age range: 16-18 years | Cross-Sectional | **Homework**  Studying time assessed via self-report questionnaire. | **Neck and shoulder pain, Lower back pain, Headaches,** and **Abdominal pain** assessed via self-report questionnaire. | **Homework**  Compared to < 4 hours, > 10 hours of studying was not associated with odds of neck and shoulder pain (OR= 0.96; 95% CI: 0.74, 1.26), odds of lower back pain (OR= 1.15; 95% CI: 0.87, 1.53), odds of headaches  (OR= 1.16; 95% CI: 0.86, 1.55), and odds of abdominal pain (OR= 1.26; 95% CI: 0.91, 1.75).  Compared to < 4 hours, 8-10 hours of studying was negatively associated with odds of neck and shoulder pain (OR= 0.64; 95% CI: 0.48, 0.86) and odds of lower back pain  (OR= 0.73; 95% CI: 0.54, 0.98), but not associated with odds of headaches (OR= 0.95; 95% CI: 0.7, 1.29) and odds of abdominal pain (OR= 0.95; 95% CI: 0.67, 1.36).  Compared to < 4 hours, 6-8 hours of studying was negatively associated with odds of neck and shoulder pain (OR= 0.6; 95% CI: 0.44, 0.82) and odds of lower back pain (OR= 0.48; 95% CI: 0.34, 0.68), buy not associated with odds of headaches (OR= 0.67; 95% CI: 0.47, 0.94) and odds of abdominal pain (OR= 0.86; 95% CI: 0.59, 1.27).  Compared to < 4 hours, 4-6 hours of studying was not associated with odds of neck and shoulder pain (OR= 0.92; 95% CI: 0.65, 1.28), odds of lower back pain (OR= 1.26; 95% CI: 0.89, 1.77), odds of headaches  (OR= 0.95; 95% CI: 0.66, 1.37), and odds of abdominal pain (OR= 1.18; 95% CI: 0.79, 1.77).  Having extra learning tasks after class was positively associated with odds of headaches (OR= 1.44; 95% CI: 1.20, 1.73), odds of lower back pain (OR= 1.33; 95% CI: 1.11, 1.59), odds of neck and shoulder pain  (OR= 1.33; 95% CI: 1.12, 1.58), but not associated with odds of abdominal pain (Values not reported). |
| Zhang et al. 2020; China | N=2623; age range: 6-9 years | Cross-Sectional | **Homework**  Homework time assessed via proxy-report questionnaire. | **Myopia**  Pupils participated in an ophthalmic examination with autorefractor applied with cycloplegia. The cycloplegic refraction is measured using tropicamide phenylephrine eye drops every 5 min, 3 times, and then refractive error is measured 30 min after the first drop of tropicamide by autorefractor with five repeated measurements. | **Homework**  Homework (> 3 hours) was not associated with myopia (β= 0.079; 95% CI: -0.213, 0.76; p= 0.268), but positively associated with risk of myopia (RR= 1.692; 95% CI: 1.079, 2.652; p= 0.022).  Homework (2-3 hours) was not associated with myopia (β= -0.063; 95% CI: -0.285, 0.113; p= 0.393), but positively associated with risk of myopia (RR= 1.44; 95% CI: 1.127, 1.840; p= 0.004). |

| **Supplementary Table 6: Social-Emotional Indicators** | | | | | |
| --- | --- | --- | --- | --- | --- |
| **Study** | **Sample** | **Study Design** | **Exposure** | **Outcome** | **Main Findings** |
| Meyer et al. 2014; Switzerland | N=289; grade 1 and 5 | Clustered RCT (follow-up: 3 years) | **Additional PA**  Intervention: Children had three physical education lessons per week (45 minutes each) given by the usual classroom teachers and two additional physical education lessons (45 minutes each) on the remaining school days that were taught by physical education teachers. In addition, three to five short activity breaks (two to five minutes each) were introduced every day during academic lessons. The children also received daily physical activity homework of about 10 minutes.  Control: Children had three physical education lessons per week (45 minutes each) given by the usual classroom teachers. | **Quality of Life (physical and psychological)** assessed via the self-report Child Health Questionnaire at baseline and three year follow-up. | **Additional PA**  There was no significant intervention effect on quality of life (physical) (Adjusted mean difference= 0.91; 95% CI: -1.473, 3.293; p= 0.45), quality of life (psychological) (Adjusted mean difference = 1.424; 95% CI: ‑0.661, 3.509; p= 0.18). |
| Riley et al. 2015; Australia | N=54; mean age: 10.53 years | Clustered RCT (follow-up: 6 weeks) | **Active lessons** Intervention: The integration of PA within pre-existing mathematics program for 6-weeks with 3 × 60 min sessions per week.  Control: Routine mathematics program. | **On task behaviour** was observed using a momentary time sampling procedure at baseline, midpoint (3 weeks) and post-test (6 weeks). Six students per class group were selected at random. Two trained research assistant observers observed simultaneously. | **Active lessons**  Intervention group increased % of time on task in math class at 6 weeks, compared to control group (Control: 4.3, Intervention: 24.2, Difference= 19.9; 95% CI: Control: -8.6, 17.1, Intervention: 12.3, 36.0, Difference: 2.4, 37.4).  No difference at 3 weeks (Control: 5.3, Intervention: 20.2, Difference: 14.9; 95% CI: Control: -8.9, 19.5, Intervention: 3.9, 36.6, Difference: -6.7, 36.5). |
| Sherry et al. 2020; England | N=49; mean age: 9.7 years | Non-Randomized Intervention (follow-up: 4 months) | **Standing desk** Intervention: One school was allocated to receive the intervention. All pupils within a single class received an height adjustable sit-stand desk that allows the user to manually shift between sitting and standing.   Control: The other school, a single class functioned as a control group, continuing with traditional classroom furniture and normal lessons. | **Behavior-related mental health** assessed via the proxy-report Strength and Difficulties questionnaire. | **Standing desk**  There was a significant deterioration in behavior-related mental health measures in the intervention group relative to the control group at 4 months (β= 5.31; 95% CI: 2.55, 8.08; p= 0.001), at 8 months (β= 7.92; 95% CI: 5.18, 10.66; p= 0.001). |
| Babic et al. 2017; Australia | N=322; mean age: 14.4 years | Longitudinal (follow-up: 6 months) | **Homework** Computer use for homework assessed via self-report questionnaire. | **Physical self-concept** assessed via the self-report subscale from the Marsh's Physical Self-Description Questionnaire.  **Psychological difficulties** assessed via the self-report Strength and Difficulties Questionnaire.  **Psychological well-being** assessed via the self-report Deiner and colleagues' Flourishing Scale. | **Homework**  There were no associations between homework and physical self-concept at 6 months (B= 0.019; SE= 0.012; p= 0.124), between homework and psychological difficulties at 6 months (B= -0.001; SE= 0.023; p= 0.968), between homework and psychological well-being at 6 months (B= -0.002; SE= 0.015; p= 0.874). |
| Carlson et al. 2015; USA | N=1322; mean age: 8.8 years | Longitudinal (duration: 5-9 months) | **Active breaks** Teacher reported physical activity breaks in the past week. | Thirteen social-emotional outcomes were assessed by the teacher-reported 10-item questionnaire adopted from the 60-item Classroom Behavior and Assets Scale. Questions included four asset items reflecting positive behaviors (items 1–4), and six problem behavior items (items 5-10).  **Student behavior:**  1. Pay attention in class  2. Cooperate with peers, ability to work with others  3. Have a positive, cheerful attitude  4. Produce work and assignments that are high quality  5. Are defiant or noncompliant  6. Lack effort or motivation or give up easily  7. Have excessive movement or are out of seat often  8. Are off task or inattentive during class time  9. Are unable to change activities or make transitions smoothly  10. Are unhappy, sad or depressed  Three social-emotional outcomes were assessed using ten social-emotional outcomes described above:   - Assets Scale (mean of items 1-4) - Problem Behavior Scale (mean of items 5-10) - Abbreviated Problem Behavior Scale (mean of items 6, 8-10). | **Active breaks**  Teacher reported PA breaks in the past week were negatively associated with ‘Lack effort or motivation or give up easily’ (β= ‑0.17; 95% CI: -0.33, -0.01; P= 0.042).  Teacher reported PA breaks in the past week were not associated with 'Abbreviated Problem Behavior Scale' (β= -0.12; 95% CI: ‑0.28, 0.05; p= 0.163), 'Are defiant or noncompliant ' (β= 0.04; 95% CI: -0.12, 0.20; p= 0.659), 'Are off task or inattentive during class time' (β= ‑0.06; 95% CI: -0.23, 0.10; p= 0.49), 'Are unable to change activities or make transitions smoothly' (β= ‑0.07; 95% CI: -0.23, 0.09; p= 0.401), 'Are unhappy, sad or depressed' (β= -0.09; 95% CI: -0.25, 0.07; p= 0.276), 'Assets Scale' (β= 0.01; 95% CI: ‑0.16, 0.17; p= 0.941), 'Cooperate with peers, ability to work with others' (β= -0.05; 95% CI: -0.22, 0.11; p= 0.531), 'Have a positive, cheerful attitude ' (β= 0.09; 95% CI: ‑0.07, 0.25; p= 0.278), 'Have excessive movement or are out of seat often' (β= ‑0.07; 95% CI: -0.23, 0.10; p= 0.421),  'Pay attention in class' (β= -0.07; 95% CI: -0.23, 0.09; p= 0.382), 'Problem Behavior Scale' (β= ‑0.09; 95% CI: -0.25, 0.07; p= 0.28), 'Produce work and assignments that are high quality' (β= 0.06; 95% CI: -0.10, 0.22; p= 0.473). |
| Hamer et al. 2016; United Kingdom | N=2038; age (baseline): 16 years | Longitudinal (follow-up: 26 years) | **Homework** Homework time assessed via self-report questionnaire. | **Psychological distress** assessed via the self-report Malaise Inventory consisting of items on depressive mood and anxiety.  **Positive mental wellbeing** assessed via the Warwick-Edinburgh Mental Well-being Scale (WEMWBS). | **Homework**  Compared to no homework at age 16, there was a negative association between up to 2 hours of homework at age 16 and psychological distress at age 42 (B= ‑0.26; 95% CI: ‑0.46, ‑0.07), but there were no associations between =2 hours of homework at age 16 and mental wellbeing at age 42 (B= 0.53; 95% CI: -0.47, 1.52), between =2 hours of homework at age 16 and psychological distress at age 42 (B= -0.14; 95% CI: -0.37, 0.08), between up to 2 hours of homework at age 16 and mental wellbeing at age 42 (B= 0.45; 95% CI: -0.43, 1.33).  There was a negative trend for homework time at age 16 and psychological distress at age 42 (p= 0.027), but no trend for homework time at age 16 and mental wellbeing at age 42 (p= 0.23).  Note: Statistical analysis used was not reported and linear regression was assumed for B. |
| Maume, 2017; United States | N=974; age: 12 years, follow-up age: 15 years | Longitudinal (follow-up: 3 years) | **Homework** Homework time assessed via self-report questionnaire. | **Depressive symptoms** assessed via the self-report short form of the Child Depression Inventory. | **Homework**  There was a positive association between change in homework from age 12-15 and depressive symptoms at age 15 (β= 0.1), but no association between homework at age 12 and depressive symptoms at age 15 (β= 0.05). |
| Anez et al. 2018; Spain | N=1501; mean age: 14.2 years | Cross-Sectional | **Homework** Weekday and weekend computer use for homework assessed via self-report questionnaire from the EAT Project Inventory. | **Body dissatisfaction** assessed via the self-report BD subscale from the Eating Disorders Inventory-3 (EDI-3). | **Homework**  Weekday homework was negatively associated with body dissatisfaction, for females (B= -0.69; 95% CI: -1.21, -0.18; p= 0.009).  There were no associations between weekday homework and body dissatisfaction, for males (B= -0.06; 95% CI: -0.52, 0.39, p= 0.781), between weekend homework and body dissatisfaction, for males (B= 0.09; 95% CI: -0.39, 0.57; p= 0.705), between weekend homework and body dissatisfaction, for females (B= 0.34; 95% CI: -0.18, 0.86; p= 0.196). |
| Calpbinici & Arslan, 2018; Turkey | N=426; mean age: 16.05 years | Cross-Sectional | **Homework** Homework assessed via self-report questionnaire. | **Anxiety, hostility, negative self-esteem, somatization, and depression** assessed via the self-report Brief Symptom Inventory (BSI). | **Homework**  Compared to those that did not use the internet for studying, those that used the internet for studying had lower anxiety scores (T-score= -3.445; p= 0.001), lower hostility scores (T-score= ‑3.344; p= 0.001), lower negative self-esteem scores (T-score= -2.789; p= 0.006), lower somatization scores (T-score= -2.415; p= 0.017), lower depression scores (T-score= -3.08; p= 0.002). |
| da Costa et al. 2020; Brazil | N=1010; mean age: 16.4 years | Cross-Sectional | **Homework** Screen-based studying assessed via self-report questionnaire. | **Health-Related Quality of Life** (HRQoL) assessed via the self-report Kidscreen-10. | **Homework**  Spending >4 hours/day studying on screens was not associated with health-related quality of life scores (β= 0.01; 95% CI: -1.30, 1.33).  Spending 2-4 hours studying on screens was not associated with health-related quality of life scores (β= 0.43; 95% CI: -0.64, 1.50). |
| Ellis, Dumas, & Forbes, 2020; Canada | N=1054; mean age: 16.68 years | Cross-Sectional | **Homework** School work during the COVID-19 pandemic assessed via self-report questionnaire. | **COVID-19 Stress** assessed via self-report questionnaire.  **Loneliness** assessed via the self-report revised UCLA Loneliness Scale.  **Depression** assessed via the self-report six-item depression subscale from the Brief Symptom Inventory (BSI). | **Homework**  There was a positive association between school work and COVID-19 stress (r= 0.06).  School work was negatively associated with loneliness (B= -0.02; SE= 0.01; p= 0.05), depression (B= -0.04; SE: 0.02; p= 0.048). |
| Gilchrist et al. 2021; Canada | N=46413; grade 9 to 12 | Cross-Sectional | **Homework** Homework time assessed via self-report questionnaire. | **Anxiety** assessed via the self-report Generalised Anxiety Disorder 7-item Scale (GAD-7).  **Flourishing** assessed via the self-report Flourishing Scale  **Depression** assessed via the self-report 10-item Center for Epidemiologic Studies Depression Scale Revised 10 (CESD-R-10). | **Homework**  Among those with <8 hours of sleep,  -Adding 15 minutes of homework (partition model, holding all screen time, MVPA, and sleep constant) was associated with higher anxiety (B= 0.07; 95% CI: 0.06, 0.08), higher flourishing (B= 0.12; 95% CI: 0.11, 0.13), lower depression (B= -0.06; 95% CI: ‑0.07, -0.05).  -Adding 15 minutes of homework while subtracting 15 minutes of MVPA was associated with higher anxiety, (B= 0.05; 95% CI: 0.04, 0.07), lower flourishing (B= -0.03; 95% CI: -0.04, -0.01), lower depression (B= -0.03; 95% CI: -0.05, -0.02).  -Adding 15 minutes of homework while subtracting 15 minutes of screen time was associated with higher anxiety, (B= 0.05; 95% CI: 0.04, 0.06), higher flourishing (B= 0.13; 95% CI: 0.12, 0.15), lower depression (B= -0.07; 95% CI: -0.08, -0.06).  -Adding 15 minutes of homework while subtracting 15 minutes of sleep was associated with higher anxiety, (B= 0.09; 95% CI: 0.07, 0.10), higher flourishing (B= 0.05; 95% CI: 0.03, 0.07), higher depression (B= 0.09; 95% CI: 0.08, 0.11).  -Homework duration was positively associated with anxiety (B= 0.06; 95% CI: 0.05, 0.07), positively associated with flourishing (B= 0.13; 95% CI: 0.12, 0.14), but negatively associated with depression (B= -0.06; 95% CI: -0.07, -0.05).  Among those with = 8 hours sleep,  -Adding 15 minutes of homework (partition model, holding all screen time, MVPA, and sleep constant) was associated with higher anxiety (B= 0.05; 95% CI: 0.04, 0.06), higher flourishing (B= 0.11; 95% CI: 0.10, 0.13), lower depression (B= -0.05; 95% CI: ‑0.06, -0.03).  -Adding 15 minutes of homework while subtracting 15 minutes of MVPA was associated with higher anxiety (B= 0.06; 95% CI: 0.05, 0.08), lower flourishing, (B= -0.03; 95% CI: -0.05, -0.01), lower depression (B= -0.05; 95% CI: -0.06, -0.03).  -Adding 15 minutes of homework while subtracting 15 minutes of screen time was associated with higher anxiety, (B= 0.04; 95% CI: 0.02, 0.05), higher flourishing (B= 0.14; 95% CI: 0.12, 0.15), lower depression (B= -0.08; 95% CI: -0.09, -0.07).  -Adding 15 minutes of homework while subtracting 15 minutes of sleep was associated with higher anxiety, (B= 0.05; 95% CI: 0.04, 0.06), higher flourishing (B= 0.09; 95% CI: 0.06, 0.12), lower depression (B = -0.05; 95% CI: -0.07, -0.02).  -Homework duration was positively associated with anxiety (B= 0.05; 95% CI: 0.04, 0.06), was positively associated with flourishing, (B= 0.13; 95% CI: 0.11, 0.14), but negatively associated with depression (B= -0.06; 95% CI: -0.07, -0.04). |
| Gu et al. 2020; USA | N=374; mean age: 9.64 years | Cross-Sectional | **Stationary time/sedentary time** Stationary time objectively measured with accelerometer (Actical activity monitors). | **Health-Related Quality of Life** assessed via the self-report PedsQL 4.0^TM^ Spanish version. | **Stationary time/sedentary time**  There was no association between stationary time and health-related quality of life (B= -0.01). |
| Lin et al. 2014; Taiwan | N=9510; mean age: 14.69 years | Cross-Sectional | **Homework**  Internet use for study assessed via self-report questionnaire. | **Suicidal attempt** and **Suicidal ideation** assessed via the self-report questions from the Epidemiological version of the Kiddie Schedule for Affective Disorders and Schizophrenia. | **Homework**  There was no association between studying online and odds of suicidal attempt (OR= 0.95; 95% CI: 0.75, 1.20; p= 0.667).  Children that did studying online had higher odds of suicidal ideation (OR= 1.19; 95% CI: 1.03, 1.38; p= 0.017). |
| Lushington et al. 2015; Australia | N=398; mean age: 17 years | Cross-Sectional | **Homework** Homework time assessed via self-report questionnaire. | **Coping** assessed via the self-report 4-item Brief Resilient Coping Scale.  **Depressive mood** assessed via the self-report 6-item Depressive Mood Scale.  **Study and interpersonal stress** assessed via the self-report 4-item Study and Interpersonal Stress subscale from the Student-life Stress Inventory. | **Homework**  There was no association between homework time and coping, in Asian-Australians (B= 0.48; SE= 0.42), depressive mood, in Caucasian-Australians (B= 0.58; SE= 0.43), and depressive mood, in Asian-Australians (B= 0.04; SE= 0.73), and study and interpersonal stress, in Asian-Australians (B= 0.12; SE= 0.57).  There was a positive association between homework time and coping, in Caucasian-Australians (B= 0.69; SE= 0.32), and study and interpersonal stress, in Caucasian-Australians (B= 1.27; SE= 0.35). |
| Rajala et al. 2019; Finland | N=420; mean age: 13.7 years | Cross-Sectional | **Stationary time/sedentary time** Stationary time objectively measured with accelerometer (ActiGraph GT3X). | **Society subjective social status** and **School subjective social status** assessed via the self-report youth version of the Subjective Social Status Scale. | **Stationary time/sedentary time**  There was a negative association between school-time sedentary time and school subjective social status (B = -0.09, SE = 0.03, p = 0.03), but no association between school-time stationary time and society subjective social status (r = -0.06). |
| Sanders et al. 2019; Australia | N=4013; age range: 10-15 years | Cross-Sectional | **Homework** Educational screen time assessed via time-use diaries administered to the child and face-to-face interview. | **Low conduct problems, Low emotional problems, Low hyperactivity, Low peer problems, and Prosociality** assessed via the proxy-report Strengths and Difficulties Questionnaire (SDQ).  **Low reactivity and Persistence** assessed via self-report School-Age Temperament Inventory.  **Social quality of life** assessed via the proxy-report Paediatric Quality of Life Inventory (PedsQL). | **Homework**  There was no association between education screen time and emotional problems (β= 0.034; 95% CI: 0.069, ‑0.001), hyperactivity (β= 0.033; 95% CI: 0.067, -0.002), conduct problems (β= 0.032; 95% CI: 0.069, -0.005), prosociality (β= -0.009; 95% CI: -0.045, 0.027), reactivity (β= 0.021; 95% CI: 0.058, -0.017), social quality of life (β= 0.013; 95% CI: -0.023, 0.049), peer problems (β= -0.002; 95% CI: 0.034, -0.039).  There was a positive association between education screen time and persistence (β= 0.056; 95% CI: 0.023, 0.090). |
| Zhang et al. 2017; China | N=17318; mean age: 9.18 years | Cross-Sectional | **Homework** Homework time assessed via the Chinese version of the International Children’s Leisure Activities Study Survey Questionnaire (CLASS-C). | **Children’s mental health status** assessed via the Chinese version of the parent-reported Strengths and Difficulties Questionnaire (SDQ). | **Homework**  There was a negative association between 1-2 hours/day of homework, compared to < 1 hour/day, and odds of poor mental health (1-2 hours associated with decreased odds) (OR= 0.7; 95% CI: 0.57, 0.85).  There was no association between ≥ 2 hours/day of homework, compared to < 1 hour/day, and odds poor mental health (OR= 1.02; 95% CI: 0.86, 1.20). |

| **Supplementary Table 7: Fitness** | | | | | |
| --- | --- | --- | --- | --- | --- |
| **Study** | **Sample** | **Study Design** | **Exposure** | **Outcome** | **Main Findings** |
| Aadland et al. 2019; Norway | N=1,129; mean age: 10.2 years | Clustered RCT (follow-up: 7 months) | **Additional Physical Activity**  Intervention: The intervention constituted three PA elements totaling an additional 165 min/week of PA: (1) physical educational lessons (3 × 30 min/week) in the subjects Norwegian, mathematics, and English, (2) PA breaks during classroom lessons (5 min/school day), and (3) PA homework (10 min/school day). Intervention length was 7 months.  Control: It was  specified to the control schools that they should carry out the amount of PA and physical education  that they would have done regardless of the study. | **Aerobic fitness**  VO_2peak_ was measured with the Andersen intermittent field running test at baseline and post-intervention.  **Motor skills**  A motor skill composite score (mean of standardized  individual scores) of three tests was determined at baseline and post-intervention: (1) Catching with one hand; (2) Throwing at a wall target; and (3) Shuttle Run 10 × 5 meters. | **Additional Physical Activity**  There was no significant intervention effect on VO_2peak_ (mean difference=6.9; 95%CI: -8.9, 22.6; p=0.387) and motor skills (mean difference=0.12; 95%CI: -0.1, 0.25, p=0.071). |
| Ekstrom et al. 2019; Sweden | N=56; age (baseline): 7-9 years | Clustered RCT (follow-up: 6 weeks) | **Active breaks** Intervention: Modified Tabata interval training. The total time of the Tabata interval training was 4 min. These 4 min were allocated as 20 s of exercise and 10 s of rest (2:1 work-rest ratio). In total, eight intervals were carried out during the 4 min and were performed by the pupils in the classroom setting. The teacher in each class chose the time during the day when the intervals were performed every school day, that is, a total of 5 days a week, during the 6-week study period.  Control: No information reported detailing the control condition. Assumed to be usual classroom conditions. | **Balance** assessed using 360° rotation on stool (i.e., participant did four 360° rotations as fast as possible) in the week before the intervention started and the week after the intervention ended.  **Coordination** assessed using a ball bounce from stool (i.e., standing on an upside-down stool bouncing a volleyball on the floor; number of bounces was counted) and “running number” test (i.e., run to specified numbered cones between 1-5 as fast as possible) in the week before the intervention started and the week after the intervention ended.  **Strength** assessed using standing long jump (i.e., jump as far as possible from stance with both feet behind mat and arms hanging freely at the side), and push-up and kneeling push-up (i.e., number of push-ups that could be done in 10 s, both regular and kneeling) in the week before the intervention started and the week after the intervention ended. | **Active breaks**  There was no significant interaction effect indicating no difference between intervention and control groups for the rotation test (F = 1.8, p = 0.19), the ball bounce (F = 0.2, p = 0.65), and the “running number” (F = 0.9, p = 0.34).  In the standing long jump test, the intervention group showed an increase, while the control group showed equal results pre- and post-intervention, demonstrated by an interaction effect (F = 7.2, p = 0.01). For both the push-up and kneeling push-up test, there was an interaction effect, showing improvements in the intervention group, while the control group displayed similar results pre- and post-intervention (F = 10.5, p = 0.002 and F = 4.4, p = 0.008) for respective variables). |
| Kolle et al. 2020; Norway | N=1,981; mean age: 14.0 years | Clustered RCT (follow-up: 9 months) | **Additional Physical Activity** Intervention 1: The PAL-intervention arm included three components: (1) An additional physical education lesson per week; (2) a 30 min/week lesson of physically active learning where play-based activities were integrated into other curriculum subjects (i.e., math, English, Norwegian); (3) a 30 min/week lesson of PA that included a variety of activities, preferably of at least moderate intensity, and it should be enjoyable.  Intervention 2: The “Don’t worry, be happy” intervention arm included two components: (1) a “Be happy” lesson and (2) a “Don’t worry” lesson (each lesson was 60min/week). Groups of 3–8 students were formed based on their hobbies. The groups were expected to choose an activity (e.g. football or handball) to perform in the chosen activity  in the “Be happy” lesson. In the “Don’t worry” lesson, the students returned  to their normal classes and either continued or introduced their class peers to their activity.  Control: The schools in the control arm continued current practice including the usual amount of mandatory physical education that was part of the curriculum. | **Aerobic fitness** assessed at baseline and at 9 months using the Andersen intermittent running test (run back and forth between two lines 16m apart) lasting for 10 min, with 15 s work periods and 15 s breaks standing still.  **Strength** in the lower body was measured at baseline and at 9 months using a standing broad jump test. Upper limb strength–handgrip strength was measured using a handheld dynamometer. Abdominal muscle endurance was tested using a sit-up test (number of correctly performed sit-ups within 30 s). | **Additional Physical Activity**  A higher aerobic fitness for Intervention 1, compared to the control group was observed at 9 months (mean difference in change = 19.7; 95%CI: 10.4, 29.0). A lower aerobic fitness for Intervention 2, compared to the control group was observed at 9 months (mean difference in change = -11.5; 95%CI: -21.9, -1.1).  There was no intervention effect on aerobic fitness observed in Intervention 1 girls, compared to control girls at 9 months (mean difference in change = 3.2; 95%CI: 10.3, 16.8; p = 0.643). A lower aerobic fitness for Intervention 2 girls, compared to control girls was observed at 9 months (mean difference in change = -24.6; 95% CI: -39.8, -9.3; p = 0.002). A higher aerobic fitness for Intervention 1 boys, compared to control boys was observed at 9 months (mean difference in change = 36.7; 95% CI: 24.0, 49.3; p = 0.001). There was no intervention effect on aerobic fitness observed in Intervention 2 boys, compared to control boys at 9 months (mean difference in change = 1.4; 95%CI: −12.6, 15.5, p = 0.842).  There was no intervention effect on standing broad jump in Intervention 1, compared to the control group (mean difference in change = 1.7; 95%CI: -0.3, 3.7) or in Intervention 2, compared to the control group (mean difference in change = 0.2; 95%CI: -2.0, 2.4) at 9 months.  A higher standing broad jump for Intervention 1 girls, compared to control girls was observed at 9 months (mean difference in change = 2.5; 95% CI: 0.1, 4.8; p = 0.036). There was no intervention effect on standing broad jump observed in Intervention 2 versus control girls at 9 months (mean difference in change = -1.1; 95%CI: -3.7, 1.4; p = 0.380). There was no intervention effect on standing broad jump observed in Intervention 1 versus control boys (mean difference in change = 1.3; 95%CI: -1.7, 4.4; p = 0.401) or in Intervention 2 versus control boys (mean difference in change = 1.2; 95%CI: -2.0, 4.4; p = 0.464) at 9 months.  A lower handgrip strength for Intervention 1, compared to the control group was observed at 9 months (mean difference in change = -1.1; 95%CI: -1.7, -0.5). There was no intervention effect for Intervention 2, compared to the control group, at 9 weeks (mean difference in change = -0.6; 95%CI: -1.2, 0.0).  A lower handgrip strength for Intervention 1 girls compared to control girls (mean difference in change = -1.8; 95%CI: 2.5, -1.1; p = 0.001) and for Intervention 2 girls compared to control girls (mean difference in change = -0.8; 95%CI: -1.5, -0.1; p = 0.039) was observed at 9 months. There was no intervention effect on handgrip strength observed in Intervention 2 versus control boys (mean difference in change = -0.1; 95%CI: -1.0, 0.7; p = 0.778) or in Intervention 2 versus control boys (mean difference in change = -0.5; 95%CI: -1.4, 0.3; p = 0.236) at 9 months.  A higher number of sit-ups for Intervention 1, compared to control the control group was observed at 9 months (mean difference in change = 0.5; 95%CI: 0.1, 0.9).There was no significant intervention effect on sit-ups observed in Intervention 2 versus the control group (mean difference in change = -0.9; 95%CI: -1.3, -0.5).  There was no intervention effect on situps observed in Intervention 1 versus control girls (mean difference in change = 0.4; 95% CI: -0.1, 1.0; p = 0.071) or Intervention 2 girls versus control girls (mean difference in change = 0.2; 95% CI: -0.8, 0.3; p = 0.459) at 9 months.  A higher number of sit-ups for Intervention 1 boys, compared to control boys was observed at 9 months (mean difference in change = 0.6; 95%CI: 0.1, 1.2; p = 0.040). There was no intervention effect observed for sit-ups in Intervention 2 versus control boys at 9 months (mean difference in change = 0.1; 95%CI: -0.6, 0.6; p = 0.913). |
| Meyer et al. 2014; Switzerland | N=289; grade 1 and 5. | Clustered RCT (follow-up: 3 years) | **Additional Physical Activity** Intervention: Three physical education lessons per week (45 minutes each) given by the usual classroom teachers and two additional physical education lessons (45 minutes each) on the remaining school days that were taught by physical education teachers. In addition, three to five short activity breaks (two to five minutes each) were introduced every day during academic lessons and children received daily PA homework of about 10 minutes.  Control: Three physical education lessons per week (45 minutes each) given by the usual classroom teachers. | **Aerobic fitness** was assessed using a 20-m shuttle run test at baseline at 3-years follow-up. | **Additional Physical Activity**  Children in the intervention arm compared with controls had a significantly higher average level of aerobic fitness at 3 years follow-up (adjusted mean difference = 0.343; 95%CI: 0.111, 0.574; p = 0.004). |
| Muller et al 2016; Germany | N=236; mean age: 11.5 years | Clustered RCT (follow-up: 4 years) | **Recess/Physical Education** Intervention: Classes were assigned to 1 unit of 45 min of physical exercise, including at least 15 minutes of endurance training, per school day (i.e., 5 units/week).  Control: The control classes receive 2 units of physical exercise per week. | **Aerobic fitness**  VO_2peak_, maximum running duration, maximum power and relative power, and heart rate were measured at baseline and follow-up with the treadmill modified Bruce protocol for children with a portable spirometry system.  **Coordination** assessed at baseline and follow-up using a body coordination test for children of four items forming one motor quotient: Balancing backwards, one-legged obstacle jumping, lateral jumping, sideways movements. | **Recess/Physical Education**  There was no significant intervention effect on VO_2peak_ (linear mixture models group difference at follow-up = 0.14; 95%CI: -3.5, 3.8; p = 0.934), maximum running duration (linear mixture models group difference at follow-up = -20.1; 95%CI: -51.7, 11.4; p = 0.189), maximum power (linear mixture models group difference at follow-up = 1.03; 95%CI: -29.4, 31.4; p = 0.942), relative power (linear mixture models group difference at follow-up = -0.34; 95%CI: -0.94, 0.26; p = 0.822) and heart rate at rest (linear mixture models group difference at follow-up = 0.023; 95%CI: -7.1, 7.1; p = 0.995).  There was no significant intervention effect on coordination motor quotient (linear mixture models group difference at follow-up = 2.67; 95%CI: -8.47, 3.12; p = 0.343). |
| Seljebotn et al. 2019; Norway | N=447; age: 9-10 years | Clustered RCT (follow-up: 10 months) | **Additional Physical Activity** Intervention: The “Active School” consisted of one primary component (physically active lessons) and two secondary components (physically active homework and physically active recess). The main component, 45-min PA lessons, was conducted 2–3 times per week, on days without physical education or other curricular PA. For the other two study intervention components, teachers were encouraged to assign homework in physical education (10 min/day) and offer inspiration for physically active recess.  Control: Control schools were asked to continue their normal routine, which included approximately 135 min/week of PA. | **Aerobic fitness** was assessed at baseline and 10 months by a 10-minute interval running test. | **Additional Physical Activity**  Children with low aerobic fitness (in first tertile of aerobic fitness) increased their running distance compared to controls at 10 months (d = 0.46; p = 0.01). However, no significant difference was found for the whole sample (p = 0.52) or for the second (p = 0.19) and third tertiles (p = 0.76). Overall, there was no significant intervention effect on aerobic fitness. |
| Harris and Chen 2018; USA | N=116;  age (baseline): 10-11 years | Non-Randomized Intervention (follow-up: 7 weeks) | **Active breaks**  Intervention 1: The intervention consisted of ‘Physical Activities Engaging the Brain’ and a Fitbit Challenge. The teacher showed a six-minute activity video once a day after the students had been sitting for 20 min, the students followed the video to immediately perform the activity for five days per week over the four-week intervention. In addition, students wore a Fitbit Charger + Heart RateTM device daily, five school days per week for four weeks.  Intervention 2: Fitbit only condition using the same procedures as Intervention 1.  Control: Neither wore the Fitbit and nor participated in any PA-related classroom breaks. Instead, they had regular classroom breaks based on the school schedule. Thus, determining the difference in school-related sedentary exposure is not possible. | **Aerobic fitness** assessed using the FitnessGram® test (i.e., Progressive Aerobic Cardiovascular Endurance Run [PACER]) at weeks 2 and 7 of the study. | **Active breaks**  There was a significant interaction between time × treatment (F = 21.946, p = 0.000, η = 0.303). Subsequently, the post hoc analysis revealed significant comparisons in the mean fitness scores from  pre- to post-test between the Physical Activities Engaging the Brain condition and the control group (F = 29.327, p = 0.000), and between the Fitbit only condition and the control group (F = 25.007, p = 0.000). |
| Chesham et al. 2018; Scotland | N=371; mean age: 8.4 years | Non-Randomized Intervention (duration: 7 months for intervention, 3 months for control) | **Active breaks** Intervention: The daily mile intervention involves children going outside, at a time of the classroom teacher’s choosing, for ~15 min of exercise at a pace self-selected by each individual child.  Control: Usual curriculum. | **Aerobic fitness** was assessed using a 20-m shuttle run test at baseline and post-intervention. | **Active breaks**  The intervention school increased shuttle run distance compared to control (Mean difference in change (follow-up - baseline) = 37.2; 95% CI = 20.1, 54.3; p =0.0460). |
| Burns et al. 2019; USA | N=435; mean age: 8.4 years | Cross-Sectional | **Stationary time/sedentary time** Stationary time objectively measured with accelerometer (wGT3X-BT). | **Motor skills**  The Test for Gross Motor Development-third edition (TGMD-3) was used to assess gross locomotor skills. These were divided into a locomotor subtest score and a ball skills subtest score. | **Stationary time/sedentary time**  The proportion of stationary time was not associated with overall TGMD-3 scores (Compositional multilevel general linear mixed-effects pivot coordinates γ-coefficient = 5.05, p = 0.26), and locomotor (γ-coefficient = 9.07, p = 0.39) and ball subset scores (γ-coefficient = 2.09, p = 0.61). |
| Cheung et al. 2019; USA | N=905 schools (Approx. N=52,4700); grade 1 to 5. | Cross-Sectional | **Active breaks** In-class PA time was obtained from School PA Survey questions. | **Aerobic fitness** obtained from the publicly available 2013-2014 Georgia FitnessGram® (i.e., PACER) test data. | **Active breaks**  There was no association between in-class PA breaks and healthy aerobic capacity classification, for girls (prevalence ratio = 0.99; 99% CI: 0.94, 1.05) and boys (prevalence ratio = 1.00; 99% CI: 0.96, 1.03). |
| Gu et al. 2020; USA | N=374; mean age: 9.6 years | Cross-Sectional | **Stationary time/sedentary time** Stationary time objectively measured with accelerometer (Actical). | **Aerobic fitness** was assessed using the PACER test from the FitnessGram® test battery. | **Stationary time/sedentary time**  There was a negative association between stationary time and aerobic fitness (Hierarchical linear regression B-coefficient = -0.04, β-coefficient = -0.22, p < 0.01). |
| Huang, Zeng, and Ye 2019; China | N=1,164; mean age: 9.6 years | Cross-Sectional | **Homework** Total study-based sedentary behaviours and study-based sedentary behaviours on weekdays and weekends were measured with a self-administered sedentary behaviour questionnaire that was developed based on Chinese adapted scale version of the Adolescent Sedentary Activity Questionnaire. | **Composite physical fitness** was created from physical fitness, including vital capacity, standing long jump, 50 m run, flexibility, sit-up (for girls) or pull-up (for boys), and 800m(for girls) or 1,000m(for boys) run tests, performed according to the Chinese National Student Physical Fitness Standard (CNSPFS) battery at the end of 2015 autumn semester. | **Homework**  There was no significant association between study-based sedentary behaviors and physical fitness, for boys (linear mixed-effects models β-coefficient = -0.05; SE: 0.14) and girls (linear mixed-effects models β-coefficient = 0.12; SE: 0.12).  There was no significant association between weekday study-based sedentary behaviors and physical fitness, for boys (linear mixed-effects models β-coefficient = -0.07; SE: 0.19) and girls (linear mixed-effects models β-coefficient = 0.16; SE: 0.15).  There was no significant association between weekend day study-based sedentary behaviors and physical fitness, for boys (linear mixed-effects models β-coefficient = 0.02; SE: 0.31) and girls (linear mixed-effects models β-coefficient = 0.06; SE: 0.36). |
| Ishihara et al. 2018; Japan | N=325; age: 12-13 years | Cross-Sectional | **Homework** Learning duration was assessed using the time spent on learning after school on weekdays and weekends, and the use of a cram school or private teacher through the use of a questionnaire. | **Aerobic fitness** was assessed using a 20-m shuttle run test. | **Homework**  There was no association between learning duration and aerobic fitness for the total sample (structural equation modeling, direct effect B = -0.06; SE: 0.05; p>0.05), nor for girls (structural equation modeling, direct effect B = -0.1; SE: 0.08; p>0.05) and for boys (structural equation modeling, direct effect B = -0.05; SE: 0.07; p>0.05).  There was no correlation between learning duration and aerobic fitness for the total sample (Pearson’s r = -0.01, p > 0.05), nor for girls (Pearson’s r = -0.02, p > 0.05) and for boys (Pearson’s r = -0.01, p > 0.05). |

| **Supplementary Table 8: Other Movement Behaviours** | | | | | |
| --- | --- | --- | --- | --- | --- |
| **Study** | **Sample** | **Study Design** | **Exposure** | **Outcome** | **Main Findings** |
| Calvert et al. 2019; USA | N=156; age 10-12 years | Clustered RCT (duration: one school day) | **Active breaks**  Intervention 1: “Light” condition in  which students were seated and watched one seated video followed by engaging in a light to moderate intensity video.  Intervention 2: “Moderate” condition in  which students watched one seated video followed by engaging in a high-intensity video.  Intervention 3: “Vigorous” condition where students engaged in two high-intensity videos.  Control: “Sedentary” condition in which students remained seated while watching two educational videos set to music. | **Physical activity**  The Axis 1 (vertical axis) average counts per minute were objectively measured with accelerometer (ActiGraph GT3X-BT accelerometer). This represents an average PA intensity across time for each participant, and was measured during the 10-minute intervention condition. | **Active breaks**  Counts per minute did not differ significantly between Intervention 1 and control group (Intervention 1: mean [SD] counts per minute=517.04 [228.40]; Control mean counts per minute=64.78 [76.98], p=0.216).  Counts per minute were significantly higher in the Intervention 2 than in the control group (Intervention 2: mean [SD] counts per minute=2,576.96 [1,343.51]; Control mean counts per minute=64.78 [76.98], p<0.001).  Counts per minute were significantly higher in the Intervention 3 than in the control group (Intervention 3: mean [SD] counts per minute=5,014.29 [1,923.26]; Control mean counts per minute=64.78 [76.98], p<0.001). |
| Kolle et al. 2020; Norway | N=1,981; mean age: 14.0 years | Clustered RCT (duration: 9 months, follow-up: 1 year) | **Additional physical activity** Intervention 1: The PAL-intervention arm included three components: (1) An additional physical education lesson per week; (2) a 30 min/week lesson of physically active learning where play-based activities were integrated into other curriculum subjects (i.e., math, English, Norwegian); (3) a 30 min/week lesson of PA that included a variety of activities, preferably of at least moderate intensity, and it should be enjoyable.  Intervention 2: The “Don’t worry, be happy” intervention arm included two components: (1) a “Be happy” lesson and (2) a “Don’t worry” lesson (each lesson was 60min/week). Groups of 3–8 students were formed based on their hobbies. The groups were expected to choose an activity (e.g. football or handball) to perform in the chosen activity  in the “Be happy” lesson. In the “Don’t worry” lesson, the students returned to their normal classes and either continued or introduced their class peers to their activity.  Control: The schools in the control arm continued current practice including the usual amount of mandatory physical education that was part of the curriculum.  Intervention length was 9 months. | **Moderate‐to‐vigorous-intensity physical activity**  Objectively measured with accelerometer (ActiGraph GT3X+) at baseline and 12-months follow-up.  **Physical activity**  Objectively measured with accelerometer (ActiGraph GT3X+) at baseline and 12-months follow-up.  **School moderate‐to‐vigorous-intensity physical activity**  Objectively measured with accelerometer (ActiGraph GT3X+) at baseline and 12-months follow-up.  **School physical activity**  Objectively measured with accelerometer (ActiGraph GT3X+) at baseline and 12-months follow-up. | **Additional physical activity**  A higher total MVPA for Intervention 1, compared to the control group was observed at 12 months (mean difference in change=4.7; 95%CI: 0.6, 8.8). There was no intervention effect on total MVPA observed in the Intervention 2, compared to the control group at 12 months (mean difference in change=-0.8; 95%CI: -5.2, 3,6).  There was no intervention effect on total MVPA observed in Intervention 1 girls, compared to control girls (mean difference in change=4.7; 95% CI: -0.5, 9.5; p=0.052) and for Intervention 1 boys, compared to control boys (mean difference in change=4.0; 95% CI: -3.1, 11.2; p=0.268) at 12 months. There was no intervention effect on total MVPA observed in Intervention 2 girls, compared to control girls (mean difference in change=1.7; 95% CI: -6.9, 3.4; p=0.510) and for Intervention 2 boys, compared to control boys (mean difference in change=0.1; 95% CI: -7.5, 7.7; p=0.981) at 12 months.  A higher total PA for Intervention 1, compared to the control group was observed at 12 months (mean difference in change=34.7; 95%CI: 4.1, 65.3). There was no intervention effect on total PA observed in the Intervention 2, compared to the control group at 12 months (mean difference in change=-7.3; 95%CI: -40.2, 25.6).  There was no intervention effect on total PA observed in Intervention 1 girls, compared to control girls (mean difference in change=27.6; 95% CI: -6.8, 62.0; p=0.116) and for Intervention 1 boys, compared to control boys (mean difference in change=44.0; 95% CI: -11.5, 100.8; p=0.119) at 12 months. There was no intervention effect on total PA observed in Intervention 2 girls, compared to control girls (mean difference in change=-11.8; 95% CI: -49.4, 25.7; p=0.537) and for Intervention 2 boys, compared to control boys (mean difference in change=-3.3; 95% CI: -62.7, 56.1; p=0.913) at 12 months.  A higher school MVPA for Intervention 1, compared to the control group was observed at 12 months (mean difference in change=5.6; 95%CI: 3.5, 7.7). There was no intervention effect on school MVPA observed in the Intervention 2, compared to the control group at 12 months (mean difference in change=0.4; 95%CI: -1.9, 2.6).  A higher school MVPA for Intervention 1 girls, compared to control girls (mean difference in change=6.3; 95% CI: 3.8; 8.8; p=0.001) and for Intervention 1 boys, compared to control boys was observed at 12 months (mean difference in change=5.1; 95% CI: 1.5, 8.6; p=0.005). There was no intervention effect on school MVPA observed in Intervention 2 girls, compared to control girls (mean difference in change=1.0; 95%CI: -1.6, 3.7; p=0.462) and for Intervention 2 boys, compared to control boys (mean difference in change=-0.4; 95% CI: -4.1, 3.3; p=0.842) at 12 months.  A higher school PA for Intervention 1, compared to the control group was observed at 12 months (mean difference in change=86.4; 95%CI: 52.1, 120.7). There was no intervention effect on school PA observed in the Intervention 2, compared to the control group at 12 months (mean difference in change=-25.4; 95%CI: -62.1, 11.3).  A higher school PA for Intervention 1 girls, compared to control girls (mean difference in change=92.3; 95% CI: 52.2, 132.5; p=0.001) and for Intervention 1 boys, compared to control boys was observed at 12 months (mean difference in change=76.7; 95% CI: 18.8, 134; p=0.009). There was no intervention effect on school PA observed in Intervention 2 girls, compared to control girls (mean difference in change=-9.4; 95% CI: -53.0, 34.0; p=0.670) and for Intervention 2 boys, compared to control boys (mean difference in change=-49.9; 95% CI: -110.5, 10.5; p=0.106) at 12 months. |
| Meyer et al. 2014; Switzerland | N=289; grade 1 and 5. | Clustered RCT (duration: 3 years) | **Additional physical activity** Intervention: Three physical education lessons per week (45 minutes each) given by the usual classroom teachers and two additional physical education lessons (45 minutes each) on the remaining school days that were taught by physical education teachers. In addition, three to five short activity breaks (two to five minutes each) were introduced every day during academic lessons and children received daily PA homework of about 10 minutes.  Control: Three physical education lessons per week (45 minutes each) given by the usual classroom teachers. | **Moderate‐to‐vigorous-intensity physical activity**  Objectively measured with accelerometer (ActiGraph MTI/CSA 7164/GT1M) at baseline and 3-year follow-up.  **Physical activity**  Objectively measured with accelerometer (ActiGraph MTI/CSA 7164/GT1M) at baseline and 3-year follow-up. | **Additional physical activity**  There was no significant intervention effect on total MVPA (mean difference=0.143; 95%CI: -0.204, 0.490, p=0.42) and total PA (mean difference=0.32; 95%CI: -0.012, 0.651, p=0.06) at 3 years. |
| Müller et al 2016; Germany | N=236; mean age: 11.5 years | Clustered RCT (duration: 4 years) | **Recess/Physical education** Intervention: Classes were assigned to 1 unit of 45 min of physical exercise, including at least 15 minutes of endurance training, per school day (i.e., 5 units/week).  Control: The control classes receive 2 units of physical exercise per week. | **Leisure activity index**  Participants answered a questionnaire evaluating PA in general and in leisure time containing 13 items at baseline and 4-year follow-up.  **Physical exercise index**  Participants answered a questionnaire evaluating PA in general and in leisure time containing 13 items at baseline and 4-year follow-up. | **Recess/Physical education**  There was no significant intervention effect on leisure activity index (mean difference=-0.01; 95%CI: -0.19, 0.17, p=0.929) and physical exercise index (mean difference=-0.10; 95%CI: -0.24, 0.04, p=0.174) at 4 years. |
| Norris et al. 2015; England | N=85; mean age: 9.5 years | Clustered RCT (duration: one day) | **Active lessons**  Intervention: Active field trip group. A teacher-operated virtual field trip was created using Google Earth. Intervention pupils stood throughout the 30-minute session, completing prompted activities such as running the 100 m finals on-the-spot in the Olympic Stadium or flapping their arms when ‘flying’ to the next location.  Control: Sedentary field trip group. A teacher-operated virtual field trip was created using Google Earth. Control participants were seated throughout the session and completed no related activities. | **Accelerometer X-counts during field trip**  Objectively measured with accelerometer (Actigraph GT1M). The X/anteroposterior axis reflects vertical movements such as jumping.  **Accelerometer Y-counts during field trip**  Objectively measured with accelerometer (Actigraph GT1M). The Y axis reflects accelerating, ambulatory movement such as running.  **Light-intensity physical activity after virtual field trip** Objectively measured with accelerometer (Actigraph GT1M).  **Moderate-intensity physical activity after virtual field trip** Objectively measured with accelerometer (Actigraph GT1M).  **Vigorous-intensity physical activity after virtual field trip** Objectively measured with accelerometer (Actigraph GT1M).  **Light-intensity physical activity during virtual field trip**  Objectively measured with accelerometer (Actigraph GT1M).  **Moderate-intensity physical activity during virtual field trip**  Objectively measured with accelerometer (Actigraph GT1M).  **Vigorous-intensity physical activity during virtual field trip** Objectively measured with accelerometer (Actigraph GT1M).  **Light-intensity physical activity during school day, including field trip**  Objectively measured with accelerometer (Actigraph GT1M).  **Moderate-intensity physical activity during school day, including field trip**  Objectively measured with accelerometer (Actigraph GT1M).  **Vigorous-intensity physical activity during school day, including field trip**  Objectively measured with accelerometer (Actigraph GT1M). | **Active lessons**  Higher X-axis counts recorded during the field trip in the intervention group, compared to the control group (mean [SD] Intervention: 22,308.53 [8,635.34], Control: 11,754.42 [7,742.71], t(81)=5.87, p < 0.001).  Higher X-axis counts recorded during the field trip in the intervention group, compared to the control group (mean [SD] Intervention: 16,804.21 [9,684.87], Control: 8,826.29 [7,742.71], t(81)=4.21, p < 0.001).  There was no difference in LPA after the field trip in the intervention group, compared to the control group (mean [SD] Intervention: 15.89 [5.42], Control: 13.15 [4.99], p=0.11).  The intervention group had more MPA after the field trip, compared to the control group (mean [SD] Intervention: 0.88 [0.81], Control: 0.55 [0.48], p=0.004).  The intervention group had less VPA after the field trip, compared to the control group (mean [SD] Intervention: 0.15 [0.33], Control: 0.71 [0.46], p < 0.001).  LPA was higher during the field trip in the intervention group, compared to the control group (β=-0.39, p ≤ 0.001).  MPA was higher during the field trip in the intervention group, compared to the control group (β=-0.22, p < 0.05).  VPA was higher during the field trip in the intervention group, compared to the control group (β=-0.35, p ≤ 0.001).  There was no difference in LPA during the school day, including the field trip, in the intervention group, compared to the control group (mean [SD] Intervention: 113.90 [21.51], Control: 109.65 [28.26], p=0.450).  There was no difference in MPA during the school day, including the field trip, in the intervention group, compared to the control group (mean [SD] Intervention: 21.41 [8.07], Control: 21.56 [10.75], p=0.947).  There was no difference in VPA during the school day, including the field trip, in the intervention group, compared to the control group (mean [SD] Intervention: 15.11 [9.46], Control: 13.54 [7.73], p=0.409). |
| Parrish et al. 2018; Australia | N=88; mean age: 14.7 years | Clustered RCT (duration: 5 months) | **Standing desk**  Intervention: The intervention comprised four components: 1) new furniture designed to reduce sitting, 2) standing strategies (e.g., 30 min/day at standing desks), 3) structured learning modules sup-porting behavior change and 4) optional standing strategies (e.g., standing assemblies, outdoor lessons)  Control: Control schools continued with their usual school program. | **Breaks in sitting/day** Objectively measured with posture-based activPAL3 at baseline and 5-months follow-up.  **Standing**  Objectively measured with posture-based activPAL3 at baseline and 5-months follow-up.  **Stepping**  Objectively measured with posture-based activPAL3 at baseline and 5-months follow-up. | **Standing desk**  There was no significant intervention effect on number of sitting breaks/day (mean difference [SD]=4.10 [9.66], p=0.78), proportion of the day standing (mean difference [SD]=-0.30 [8.11], p=0.19), and proportion of the day stepping (mean difference [SD]=3.00 [4.54], p=0.66) at 5-months follow-up. |
| Resaland et al. 2016; Norway | N=1,129; mean age: 10.2 years | Clustered RCT (duration: 7 months) | **Additional physical activity**  Intervention: Three components aimed at providing children with the opportunity to engage in 165 min of PA/week more than the control group: 1) physically active lessons in three core subjects for 90 min/week, conducted in the playground; 2)  PA breaks (5 min/day) implemented in the classroom during academic lessons; and 3) PA homework (10 min/day) prepared  by teachers. In addition, pupils attending I-schools participated in the curriculum-prescribed 90 min/week of PE and the curriculum-pre-  scribed 45 min/week of PA.  Control: Schools were asked to provide the “normal practice” school curriculum, including usual amounts of PA/PE, being approximately 135 min/week." | **Light-intensity physical activity**  Objectively measured with accelerometer (ActiGraph GT3X+) at baseline and 7-months follow-up.  **Moderate‐to‐vigorous-intensity physical activity**  Objectively measured with accelerometer (Actigraph GT3X+) at baseline and 7-months follow-up.  **Physical activity**  Total counts per minute were objectively measured with accelerometer (Actigraph GT3X+) at baseline and 7-months follow-up.  **School light-intensity physical activity**  Objectively measured with accelerometer (ActiGraph GT3X+) at baseline and 7-months follow-up.  **School moderate‐to‐vigorous-intensity physical activity**  Objectively measured with accelerometer (Actigraph GT3X+) at baseline and 7-months follow-up.  **School physical activity**  Total counts per minute were objectively measured with accelerometer (Actigraph GT3X+) at baseline and 7-months follow-up. | **Additional physical activity**  There was no significant intervention effect (all p ≥ 0.370) on total LPA (mean difference=-1.1; 95%CI: -5.2, 2.8, total MVPA (mean difference=-1.1; 95%CI: -6.1, 4.0, and total PA (-2.1; 95%CI: -47.5, 43.2) at 7-months follow-up.  There was no significant intervention effect (all p ≥ 0.399) on school LPA (mean difference=0.9; 95%CI: -1.2,3.0, school MVPA (mean difference=0.1; 95%CI: -3.0, 3.2, and school PA (13.5; 95%CI: -36.9, 64.0) at 7-months follow-up. |
| Reznik et al. 2015; USA | N=988; mean age: 6.0 years | Clustered RCT (duration: 8 weeks) | **Additional physical activity**  Intervention: Schools received an audio CD consisting of 10-minute, education-focused aerobic activities led by teachers three times a day in addition to their regular PE classes.  Control: Schools continued to provide PE class to their students as usual. | **Steps**  Number of steps per school  day was measured at baseline and at 8-week follow-up with a pedometer (Yamax Digi-Walker  SW-200). | **Additional physical activity**  Eight weeks post–intervention, the intervention group took significantly greater mean (SD) number of steps than controls  (2839 [1262] vs. 2545 [1153]; p=0.0048).  Eight weeks post–intervention, the students in the BMI < 85th percentile intervention subgroup took significantly greater mean number of steps than controls (2833 [984] vs. 2536 [819]; p=0.019).  Eight weeks post–intervention, the students in the BMI ≥ 85th percentile intervention subgroup took significantly greater mean number of steps than controls (2911 [1004] vs. 2540 [903]; p=0.0029). |
| Ridgers et al. 2018; Australia | N=156; mean age: 11.3 years | Clustered RCT (duration: one class) | **Additional physical activity**  Schools were randomized to 3 experimental condition, occurring on 1 occasion: Additional MVPA, additional LPA, or restricted LPA and MVPA.  The additional MVPA intervention group increased MVPA in the form of a sports session delivered during morning class time; ~120-min session.  The additional LPA intervention group increased LPA through a  standing lesson delivered by the children’s schoolteacher during one class period (∼120 min).    Restricted LPA and MVPA intervention condition, a block of recess and lunchtime (∼90 min) was replaced with the schools wet weather/excessive heat protocol, sedentary indoor play activities (e.g., games, drawing). | **Energy expenditure**  Energy expenditure was objectively measured with Sensewear.  **Light-intensity physical activity**  Objectively measured with accelerometer (ActiGraph GT3X+).  **Moderate‐to‐vigorous-intensity physical activity**  Objectively measured with accelerometer (Actigraph GT3X+).  **Physical activity**  Total counts per minute were objectively measured with accelerometer (Actigraph GT3X+). | **Additional physical activity**  For the additional MVPA condition, there was evidence of more mean energy expenditure (119.7; 95% CI: 19.1, 220.4), MVPA (25.8; 95% CI, 15.3 to 36.2) and PA (160.4; 95% CI: 76.0, 244.9) between the day on which the experiment took place compared with the comparison day in the previous week. There was also evidence of a difference in mean MVPA (−26.3; 95% CI: −36.8, −15.8) and PA (−199.5; 95% CI: −284.2, −114.7) between the day on which the experiment took place compared with the following day. There was no evidence of a difference in mean energy expenditure between the day that the experiment took place and the following day (−90.6; 95% CI: −190.7, 9.5). There was no evidence of a difference between any outcomes on the day after the experiment compared with their usual activity for that day.  For the additional LPA condition, there was no evidence of an interaction between day and week for energy expenditure (β coefficient: -19.28; 95%CI: -134.89, 96.34, p>0.05), LPA (β coefficient: -9.85; 95%CI: -26.90, 7.20, p>0.05), MVPA (β coefficient: 0.12; 95%CI: -14.76, 15.00, p>0.05) or PA (β coefficient: 54.13; 95%CI: -47.40, 155.65, p>0.05).  For the restricted PA condition, there was evidence of an interaction between day and week for energy expenditure (β coefficient: 238.79; 95%CI: 122.57, 355.01, p < 0.001), LPA (β coefficient: 25.17; 95%CI: 11.19, 39.15, p < 0.001), MPA (β coefficient: 16.72; 95%CI: 3.22, 30.23, p < 0.05) and PA (β coefficient: 114.52; 95%CI: 23.53, 205.51, p < 0.05).  Post hoc analyses for the restricted PA condition showed that there was evidence of a lower mean energy expenditure (−174.9; 95% CI: −281.0, −68.7), LPA (−20.2; 95% CI: −33.1, −7.2), MVPA (−25.8; 95% CI: −38.4, −13.3), and PA (−179.5; 95% CI: −264.0, −94.9) on the day of the experiment compared with the comparison day in the previous week. In addition, there was evidence that energy expenditure (173.9; 95% CI: 66.2, 281.6), LPA (15.5; 95% CI: 2.7, 28.3), MVPA (18.2; 95% CI: 5.8, 30.6) and PA (124.0; 95% CI: 40.4, 207.5) were higher the day after the experimental condition compared with the day of the experiment. There was no evidence of a difference between any outcomes on the day after the experiment compared with their usual activity for that day. |
| Riley et al. 2015; Australia | N=54; mean age: 10.5 years | Clustered RCT (duration: 6 weeks) | **Active lessons**  Intervention: The embedding of PA across the pre-existing mathematics program for 3 × 60 min sessions per week.  Control: Usual school program. | **Mathematics light-intensity physical activity**  Objectively measured with accelerometer (Actigraph GT3X) at baseline, 3 and 6 weeks.  **Mathematics moderate-intensity physical activity**  Objectively measured with accelerometer (Actigraph GT3X) at baseline, 3 and 6 weeks.  **Mathematics moderate‐to‐vigorous-intensity physical activity**  Objectively measured with accelerometer (Actigraph GT3X) at baseline, 3 and 6 weeks.  **Mathematics vigorous-intensity physical activity**  Objectively measured with accelerometer (Actigraph GT3X) at baseline, 3 and 6 weeks.  **School light-intensity physical activity**  Objectively measured with accelerometer (Actigraph GT3X) at baseline, 3 and 6 weeks.  **School moderate-intensity physical activity**  Objectively measured with accelerometer (Actigraph GT3X) at baseline, 3 and 6 weeks.  **School moderate‐to‐vigorous-intensity physical activity**  Objectively measured with accelerometer (Actigraph GT3X) at baseline, 3 and 6 weeks.  **School vigorous-intensity physical activity**  Objectively measured with accelerometer (Actigraph GT3X) at baseline, 3 and 6 weeks. | **Active lessons**  A higher % of the mathematics class was spent in LPA in the intervention group, compared to the control group at 3 weeks (mean difference in change=17.0; 95%CI: -11.5, -4.3, p<0.001) and 6 weeks (mean difference in change=12.6; 95%CI: 8.3, 17.0, p<0.001) follow-up.  There was no intervention effect on % of the mathematics class spent in MPA observed in the intervention, compared to the control group at 3 weeks follow-up (mean difference in change=0.5; 95%CI: -0.4, 1.4, p=0.29). A higher % of the mathematics class was spent in MPA in the intervention group, compared to the control group at 6 weeks follow-up (mean difference in change=3.2; 95%CI: 2.1, 4.2, p<0.001).  A higher % of the mathematics class was spent in MVPA in the intervention group, compared to the control group at 3 weeks (mean difference in change=7.0; 95%CI: 5.4, 8.7, p<0.001) and 6 weeks (mean difference in change=9.7; 95%CI: 7.6, 11.8, p<0.001) follow-up.  A higher % of the mathematics class was spent in VPA in the intervention group, compared to the control group at 3 weeks (mean difference in change=6.5; 95%CI: 5.1, 8.0, p<0.001) and 6 weeks (mean difference in change=6.4; 95%CI: 4.8, 8.0, p<0.001) follow-up.  There was no intervention effect on % of school time spent in LPA observed in the intervention, compared to the control group at 3 weeks follow-up (mean difference in change=1.2; 95%CI: -2.7, 5.1, p=0.54). A higher % of school time was spent in LPA in the intervention group, compared to the control group at 6 weeks follow-up (mean difference in change=12.9; 95%CI: 8.2, 17.6, p<0.001)  There was no intervention effect on % of school time spent in MPA observed in the intervention, compared to the control group at 3 weeks (mean difference in change=0.5; 95%CI: -0.4, 1.4, p=0.29). A higher % of school time was spent in MPA in the intervention group, compared to the control group at 6 weeks (mean difference in change=3.2; 95%CI: 2.1, 4.2, p<0.001).  A higher % of school time was spent in MPA in the intervention group, compared to the control group at 3 weeks (mean difference in change=1.8; 95%CI: 2.7, 6.0, p<0.02) and 6 weeks (mean difference in change=4.3; 95%CI: 2.7, 6.0, p<0.001) follow-up.  A higher % of school time was spent in MVPA in the intervention group, compared to the control group at 3 weeks (mean difference in change=6.1; 95%CI: 3.3, 8.9, p<0.001) and 6 weeks (mean difference in change=8.7; 95%CI: 5.8, 11.6, p<0.001) follow-up.  A higher % of school time was spent in VPA in the intervention group, compared to the control group at 3 weeks (mean difference in change=4.3; 95%CI: 2.3, 6.3, p<0.001) and 6 weeks (mean difference in change=4.3; 95%CI: 2.6, 5.9, p<0.001) follow-up. |
| Seljebotn et al. 2019; Norway | N=447; age: 9-10 years | Clustered RCT (duration: 10 months) | **Additional physical activity**  Intervention: The intervention consisted of one primary component (physically active lessons) and two secondary components (physically active homework and physically active recess). The main component, physically active lessons, was conducted 2–3 times per week, on days without physical education or other curricular PA. All lessons lasted 45 min**.**  Control: Control schools were asked to continue their normal routine, which included approximately 135 min/week of PA. | **Counts per minute**  Objectively measured with accelerometer (ActiGraph  GT1M/GT3X/GT3X+; Axis 1) at baseline and 10-months follow-up.  **Light-intensity physical activity**  Objectively measured with accelerometer (ActiGraph  GT1M/GT3X/GT3X+) and 10-months follow-up.  **Moderate‐to‐vigorous-intensity physical activity**  Objectively measured with accelerometer (ActiGraph  GT1M/GT3X/GT3X+) and 10-months follow-up.  **Steps**  Objectively measured with accelerometer (ActiGraph  GT1M/GT3X/GT3X+) and 10-months follow-up. | **Additional physical activity**  A higher total counts per minute for the intervention group, compared to the control group was observed at 10 months (mean difference in change=60; 95%CI: 15, 105, p=0.008).  A higher total counts per minute for the intervention girls, compared to the control girls was observed at 10 months (mean difference in change=73; 95%CI: 14,132, p=0.016). There was no intervention effect on total counts per minute observed in the intervention boys, compared to the control boys at 10 months (mean difference in change=48; 95%CI: -21, 116, p=0.164).  There was no intervention effect on LPA observed in the intervention group, compared to the control group at 10 months (mean difference in change=-5; 95%CI: -12, 3, p=0.232).  There was no intervention effect on LPA observed in the intervention girls, compared to the control girls at 10 months (mean difference in change=3; 95%CI: -8, 14, p=0.587). There was no intervention effect on LPA observed in the intervention boys, compared to the control boys at 10 months (mean difference in change=7; 95%CI: −4, 17, p=0.244).  A higher MVPA for the intervention group, compared to the control group was observed at 10 months (mean difference in change=8; 95%CI: 3,13, p=0.001).  There was no intervention effect on MVPA observed in the intervention girls, compared to the control girls at 10 months (mean difference in change=5; 95%CI: -1, 11, p=0.102). A higher total counts per minute for the intervention boys, compared to the control boys was observed at 10 months (mean difference in change=11; 95%CI: 4,19, p=0.004).  A higher number of steps for the intervention group, compared to the control group was observed at 10 months (mean difference in change=940; 95%CI: 341, 1,540, p=0.002).  There was no intervention effect on steps observed in the intervention girls, compared to the control girls at 10 months (mean difference in change=548; 95%CI: -282, 1,377, p=0.194). A higher number of steps for the intervention boys, compared to the control boys was observed at 10 months (mean difference in change=1,328; 95%CI: 459, 2,198, p=0.003). |
| Silva et al. 2018; Portugal | N=49; mean age: 11.7 years | Clustered RCT (duration: 16 weeks) | **Standing desk** Intervention: The intervention involved replacing traditional seated classroom desks for standing desks, for a total duration of 16 weeks, in addition to performing teacher training and holding education/motivation sessions with students and parents.  Control: No details, aside from within the same school. Assumed to be typical classroom. | **Out of school light-intensity physical activity**  Objectively measured with accelerometer (ActiGraph GT3X+) at baseline and 16 weeks.  **Out of school sit-to-stand transitions**  Objectively measured with accelerometer (ActiGraph GT3X+) at baseline and 16 weeks.  **Out of school standing**  Objectively measured with accelerometer (ActiGraph GT3X+) at baseline and 16 weeks.  **Out of school step counts**  Objectively measured with accelerometer (ActiGraph GT3X+) at baseline and 16 weeks.  **Out of school stepping**  Objectively measured with accelerometer (ActiGraph GT3X+) at baseline and 16 weeks.  **School light-intensity physical activity**  Objectively measured with accelerometer (ActiGraph GT3X+) at baseline and 16 weeks.  **School sit-to-stand transitions**  Objectively measured with accelerometer (ActiGraph GT3X+) at baseline and 16 weeks.  **School standing**  Objectively measured with accelerometer (ActiGraph GT3X+) at baseline and 16 weeks.  **School step counts**  Objectively measured with accelerometer (ActiGraph GT3X+) at baseline and 16 weeks.  **School stepping**  Objectively measured with accelerometer (ActiGraph GT3X+) at baseline and 16 weeks.  **Weekend light-intensity physical activity**  Objectively measured with accelerometer (ActiGraph GT3X+) at baseline and 16 weeks.  **Weekend sit-to-stand transitions**  Objectively measured with accelerometer (ActiGraph GT3X+) at baseline and 16 weeks.  **Weekend standing**  Objectively measured with accelerometer (ActiGraph GT3X+) at baseline and 16 weeks.  **Weekend step counts**  Objectively measured with accelerometer (ActiGraph GT3X+) at baseline and 16 weeks.  **Weekend stepping**  Objectively measured with accelerometer (ActiGraph GT3X+) at baseline and 16 weeks.  **Whole week light-intensity physical activity**  Objectively measured with accelerometer (ActiGraph GT3X+) at baseline and 16 weeks.  **Whole week sit-to-stand transitions**  Objectively measured with accelerometer (ActiGraph GT3X+) at baseline and 16 weeks.  **Whole week standing**  Objectively measured with accelerometer (ActiGraph GT3X+) at baseline and 16 weeks.  **Whole week step counts**  Objectively measured with accelerometer (ActiGraph GT3X+) at baseline and 16 weeks.  **Whole week stepping**  Objectively measured with accelerometer (ActiGraph GT3X+) at baseline and 16 weeks. | **Standing desk**  There was no difference between the intervention and control group in mean change in out of school LPA, sit-to-stand transitions, standing, step counts and stepping (all group*time interaction p-values>0.05) from baseline to 16-week follow-up.  Group differences in mean change in standing time during school time between the intervention group and control group were observed (significant time*group interactions p < 0.05). There was no difference between the intervention and control group in mean change in during school LPA, sit-to-stand transitions, step counts and stepping (p-values for group*time interaction>0.05) from baseline to 16-week follow-up.    There was no difference between the intervention and control group in mean change in weekend LPA, sit-to-stand transitions, standing, step counts and stepping (all group*time interaction p-values>0.05) from baseline to 16-week follow-up.  Group differences in mean change in whole week standing time between the intervention group and control group were observed (significant time*group interactions p < 0.05). There was no difference between the intervention and control group in mean change in during school LPA, sit-to-stand transitions, step counts and stepping (p-values for group*time interaction>0.05) from baseline to 16-week follow-up. |
| Verloigne et al. 2018; Belgium | N=322; mean age: 12.9 years | Clustered RCT (duration: 6 months) | **Standing desk**  Intervention: Three standing desks  were added to each intervention class. Teachers were asked to use a rotation system to make sure that all pupils had equal access to the desks.  Control: No information reported, assumed to be usual classroom control. | **Screen-time**  Questionnaire asking about screen-time behaviours (i.e. television watching and computer use) at home at baseline, and mid- (3 months) and post-intervention (6 months).  **Standing-time (school-time)**  Right thigh worn activPAL accelerometers worn Monday or Tuesday until Friday of the same week at baseline, and mid- (3 months) and post-intervention (6 months).  **Stepping-time (school-time)**  Right thigh worn activPAL accelerometers worn Monday or Tuesday until Friday of the same week at baseline, and mid- (3 months) and post-intervention (6 months). | **Standing desk**  There was no intervention effect on screen time observed in the intervention group, compared to the control group (Beta: 12.14, SE: 17.11, p > 0.05) at 6-months.  **Standing-time (school-time)**  Intervention group increased standing time compared to control at 6 months (Beta: 34.464, SE: 10.110, p < 0.05), for primary school children. No intervention effect for secondary school children (Beta: -22.854, SE: 22.858, p > 0.05) at 6-months.  **Stepping-time (school-time)**  No intervention effect at 6-months for primary (Beta: -18.796, SE: 10.038, p > 0.05) or secondary (Beta: -16.087, SE: 11.374, p > 0.05) school children. |
| Wells, Myers, and Henderson 2014; USA | N=227; mean age: 9.3 years | Clustered RCT (duration: 18 months) | **Active lessons**  Intervention: The intervention consisted of four components: (1) The garden was a 4'×8' raised bed for each class; (2) Access to a curriculum of lessons based on a review of extant garden curricula; (3) Resources for the school included information about food safety in the garden and related topics, and; (4) The garden implementation guide provided guidance regarding planning, planting and maintaining the garden throughout the year. | **Light-intensity physical activity**  Objectively measured with accelerometer (ActiGraph GT3X+ or GT1M) at baseline and 6-month, 1-year and 18-month follow-up (waves 2,3,4).  **Moderate-intensity physical activity**  Objectively measured with accelerometer (ActiGraph GT3X+ or GT1M) at baseline and 6-month, 1-year and 18-month follow-up (waves 2,3,4).  **Moderate‐to‐vigorous-intensity physical activity**  Objectively measured with accelerometer (ActiGraph GT3X+ or GT1M) at baseline and 6-month, 1-year and 18-month follow-up (waves 2,3,4).  **Usual physical activity** was assessed using the Girls Health Enrichment Multisite Study Activity Questionnaire at baseline and 6-month, 1-year and 18-month follow-up (waves 2,3,4).  **Vigorours-intensity physical activity**  Objectively measured with accelerometer (ActiGraph GT3X+ or GT1M) at baseline and 6-month, 1-year and 18-month follow-up (waves 2,3,4).  **Yesterday’s physical activity** was assessed using the Girls Health Enrichment Multisite Study Activity Questionnaire at baseline and 6-month, 1-year and 18-month follow-up (waves 2,3,4). | **Active lessons**  There was no intervention effect on LPA observed in the intervention group, compared to the control group at post-intervention (combined waves 2, 3 & 4; mean difference in change=0.57; p=0.492).  A higher MPA for the intervention group, compared to the control group, was observed at post-intervention (combined waves 2, 3 & 4; mean difference in change=0.58; p=0.010).  A higher MVPA for the intervention group, compared to the control group, was observed at post-intervention (combined waves 2, 3 & 4; mean difference in change=1.00; p=0.044).  There was no intervention effect on usual PA observed in the intervention group, compared to the control group at post-intervention (combined waves 2, 3 & 4; mean difference in change=-0.37; p=0.083).  There was no intervention effect on VPA observed in the intervention group, compared to the control group at post-intervention (combined waves 2, 3 & 4; mean difference in change=0.44; p=0.213).  There was no intervention effect on yesterday’s PA observed in the intervention group, compared to the control group at post-intervention (combined waves 2, 3 & 4; mean difference in change=-0.20; p=0.312). |
| Ee et al. 2018; Australia | N=47; age: 10-11 years | Cross-Over Trial (duration: 21 days/condition, total: 42 days) | **Standing desk**  The class was divided into two groups—one group used the standing desks while the other group used the traditional seated desks. After 21 school days (three rotations of the school’s seven-day timetable), the students changed desks for another 21 school days. | **Light-intensity physical activity**  Objectively measured with accelerometer (ActiGraph GT9X).  **Moderate-intensity physical activity**  Objectively measured with accelerometer (ActiGraph GT9X).  **Standing during school time**  Objectively measured with accelerometer (ActiGraph GT9X).  **Stationary time**  Objectively measured with accelerometer (ActiGraph GT9X).  **Vigorous-intensity physical activity**  Objectively measured with accelerometer (ActiGraph GT9X). | **Standing desk**  There was no difference in LPA between standing desk (mean [SE]=241 [7]) and sitting desk (mean [SE]=256 [6]) conditions (p=0.111).  There was no difference in MPA between standing desk (mean [SE]=39 [2]) and sitting desk (mean [SE]=42 [2]) conditions (p=0.260).  Standing time in school was significantly higher in the standing desk condition (mean [SE]=84 [4]) compared to sitting desk condition (mean [SE]=63 [3]; p < 0.001).  There was no difference in stationary time between standing desk (mean [SE]=674 [23]) and sitting desk (mean [SE]=686 [26]) conditions (p=0.790).  There was no difference in VPA between standing desk (mean [SE]=18 [1]) and sitting desk (mean [SE]=20 [1]) conditions (p=0.330). |
| Kariippanon et al. 2019; Australia | N=203; mean age: 13.2 years | Cross-Over Trial (duration: one day/condition, total 2 days) | **Standing desk**  Students spent one day in a traditionally furnished and another day in a flexible learning space containing a variety of  furniture and layout options, such as grouped tables, standing workstations, and write-able tables and walls. | **Breaks in sitting/hour**  Objectively measured with posture-based activPAL.  **Standing**  Objectively measured with posture-based activPAL.  **Stepping**  Objectively measured with posture-based activPAL. | **Standing desk**  The number of breaks in sitting per hour was significantly greater in the flexible learning space compared to the traditional classroom (mean difference=2.13; 95%CI: 0.75, 3.51, p=003).  The proportion of time was significantly greater in the flexible learning space compared to the traditional classroom (mean difference=15.36; 95%CI: 12.68, 18.03, p=001).  The proportion of stepping time was significantly greater in the flexible learning space compared to the traditional classroom (mean difference=2.55; 95%CI: 2.02, 3.07, p=001). |
| Rees-Punia et al. 2017; USA | N=86; grade 2 to 4. | Cross-Over Trial (duration: 3 days/condition, total: 6 days) | **Active lessons**  PA was objectively (accelerometer ActiGraph GT3X) monitored on 3 school days with a garden class and 3 school days without a garden class, facilitating a within subjects’ comparison of children’s PA on garden and no-garden school days. | **Light-intensity physical activity**  Objectively measured with accelerometer (ActiGraph GT3X) and analyzed per school (A, B, C, and D).  **MET-minutes**  Objectively measured with accelerometer (ActiGraph GT3X) and analyzed per school (A, B, C, and D).  **Moderate-intensity physical activity**  Objectively measured with accelerometer (ActiGraph GT3X) and analyzed per school (A, B, C, and D).  **Vigorous-intensity physical activity**  Objectively measured with accelerometer (ActiGraph GT3X) and analyzed per school (A, B, C, and D). | **Active lessons**  Children in School A (garden class mean [SD]=29.3 [6]; non-garden class mean [SD]=37.4 [6], p<0.001), B (garden class mean [SD]=36.0 [7]; non-garden class mean [SD]=28.6 [6], p<0.001), and D (garden class mean [SD]=24.6 [6]; non-garden class mean [SD]=21.0 [6], p=0.01) had more LPA during garden class days, compared to non-garden class days. There was no difference in LPA for children in School C on garden class days and non-garden class days (garden class mean [SD]=31.6 [5]; non-garden class mean [SD]=30.6 [6], p=0.10).  Children in School A (p<0.0001), B (p<0.0001), C (p<0.05) and D (p<0.0001) had higher MET-minutes during garden class days, compared to non-garden class days.  Children in School A (garden class mean [SD]=6.1 [1]; non-garden class mean [SD]=3.5 [1], p<0.001), B (garden class mean [SD]=5.3[2]; non-garden class mean [SD]=3.5 [2], p<0.001), C (garden class mean [SD]=4.4 [2]; non-garden class mean [SD]=3.6 [2], p=0.04) and D (garden class mean [SD]=3.3 [1]; non-garden class mean [SD]=2.2 [1], p<0.001) had more LPA during garden class days, compared to non-garden class days.  Children in School A (garden class mean [SD]=2.2 [1]; non-garden class mean [SD]=1.1 [1], p<0.001), B (garden class mean [SD]=2.3 [2]; non-garden class mean [SD]=1.3 [1], p<0.001), and D (garden class mean [SD]=1.4 [1]; non-garden class mean [SD]=0.7 [1], p<0.001) had more LPA during garden class days, compared to non-garden class days. There was no difference in LPA for children in School C on garden class days and non-garden class days (garden class mean [SD]=2.9 [2]; non-garden class mean [SD]=2.5 [2], p=0.31). |
| Contardo Ayala et al. 2016; Australia | N=41; age: 11-12 yr (control mean age: 11.7 yr; intervention mean age: 11.5 yr). | Non-Randomized Intervention (duration: 8 months) | **Standing desk**  Intervention: Each participant in the intervention class was provided with a manually adjustable height-adjustable workstation.  Control: The control classroom followed standard practice using traditional  furniture. | **Classroom light-intensity physical activity**  Objectively measured with accelerometer (ActiGraph GT3X) at baseline and 8-months follow-up.  **Classroom sit-to-stand transitions**  Objectively measured with posture-based activPAL at baseline and 8-months follow-up.  **Classroom standing time**  Objectively measured with posture-based activPAL at baseline and 8-months follow-up.  **Classroom stepping time**  Objectively measured with posture-based activPAL at baseline and 8-months follow-up.  **Light-intensity physical activity**  Objectively measured with accelerometer (ActiGraph GT3X) at baseline and 8-months follow-up.  **School light-intensity physical activity**  Objectively measured with accelerometer (ActiGraph GT3X) at baseline and 8-months follow-up.  **School sit-to-stand transitions**  Objectively measured with posture-based activPAL at baseline and 8-months follow-up.  **School standing time**  Objectively measured with posture-based activPAL at baseline and 8-months follow-up.  **School stepping time**  Objectively measured with posture-based activPAL at baseline and 8-months follow-up.  **Sit-to-stand transitions**  Objectively measured with posture-based activPAL at baseline and 8-months follow-up.  **Standing time**  Objectively measured with posture-based activPAL at baseline and 8-months follow-up.  **Stepping time**  Objectively measured with posture-based activPAL at baseline and 8-months follow-up. | **Standing desk**  There was no intervention effect on classroom LPA (mean difference in change=5.78; 95%CI: -7.18, 18.73, p=0.382), sit-to-stand transitions (mean difference in change=5.21; 95%CI: -0.28, 10.7, p=0.063), standing time (mean difference in change=3.97; 95%CI: -7.24, 15.19, p=0.488), and stepping time (mean difference in change=4.62; 95%CI: -1.46, 10.70, p=0.136) observed in the intervention group, compared to the control group at 8 months.  There was no intervention effect on total LPA (mean difference in change=4.85; 95%CI: -10.87, 20.57, p=0.546).  There was no intervention effect on school LPA (mean difference in change=-3.88; 95%CI: -33.24, 25.48, p=0.796), sit-to-stand transitions (mean difference in change=9.87; 95%CI: -1.31, 21.05, p=0.084), standing time (mean difference in change=10.5; 95%CI: -34.25, 13.24, p=0.386), and stepping time (mean difference in change=-8.47; 95%CI: -34.42, 17.48, p=0.522) observed in the intervention group, compared to the control group at 8 months.  A higher number of total sit-to-stand transitions for the intervention group, compared to the control group, was observed at 8 months (mean difference in change=7.26; 95%CI: 1.2, 13.32, p=0.019).  There was no intervention effect on total standing time (mean difference in change=3.66; 95%CI: -9.49, 16.81, p=0.586) and stepping time (mean difference in change=6.24; 95%CI: -3.26, 15.73, p=0.198) observed in the intervention group, compared to the control group at 8 months. |
| Contardo Ayala et al. 2018; Australia | N=88; mean age: 14.8 years | Non-Randomized Intervention (duration: 17 weeks) | **Standing desk**  Intervention: Traditional classroom furniture in the intervention classroom was replaced with height-adjustable desks and lab stools.  Control: The control classes only used the traditional ‘seated’ furniture. | **Energy expenditure**  Objectively measured with accelerometer (Sensewear) at 4 weeks and 17-weeks follow-up. | **Standing desk**  A higher energy expenditure for the intervention group, compared to the control group, was observed at 4 weeks (mean difference in change=29.4; 95%CI: 11.1, 47.7, p<0.05) and 17 weeks (mean difference in change=37.7; 95%CI: 16.9, 58.5, p<0.05). |
| Carlson et al. 2015; USA | N=1,322; mean age: 8.8 years | Non-Randomized Intervention (duration: 5-9 months) | **Active breaks**  Intervention: Each district developed a plan unique to their district to support all classroom teachers from grades 1–6 in a minimum of two schools to implement at least one 10-minute PA break daily.  No control group. | **Moderate‐to‐vigorous-intensity physical activity**  Objectively measured with accelerometer (ActiGraph GT3X+) directly post-intervention and 6-months follow-up. | **Active breaks**  Between post-intervention and 6-month follow-up, mean school MVPA increased from 25.5 (SD=1.3) to 27.8 (SD=12.6) min/day (p < 0.001). |
| Chesham et al. 2018; Scotland | N=371; mean age: 8.4 years | Non-Randomized Intervention (duration: 7 months for intervention, 3 months for control) | **Active breaks** Intervention: The daily mile intervention involves children going outside, at a time of the classroom teacher’s choosing, for ~15 min of exercise at a pace self-selected by each individual child.  Control: Usual curriculum. | **Moderate‐to‐vigorous-intensity physical activity**  Objectively measured at baseline and one year later with five models ActiGraph accelerometers: wGT3X-BT, wGT3X+, GT3X+,  GT3X and GT1M. | **Active breaks**  A higher MVPA for the intervention group, compared to the control group, was observed at 1-year follow-up (mean difference in change=9.5; 95%CI: 5.4, 13.5, p=0.021). |
| Clemes et al. 2016, AUS study; Australia | N=48; mean age: 11.6 years | Non-Randomized Intervention (duration: 9-10 weeks) | **Standing desk**  Intervention: The Australian intervention class replaced all standard desks with standing desks.  Control: Control classrooms retained their normal seated  desks. | **Total standing time**  Waking weekday, objectively measured with posture-based activPAL3 at baseline and 9/10-weeks follow-up.  **Standing time during class time**  During class time, objectively measured with posture-based activPAL3 at baseline and 9/10-weeks follow-up.  **Total stepping time**  Waking weekday, objectively measured with posture-based activPAL3 at baseline and 9/10-weeks follow-up.  **Stepping time during class time**  During class time, objectively measured with posture-based activPAL3 at baseline and 9/10-weeks follow-up.  **Total steps**  Waking weekday, objectively measured with posture-based activPAL3 at baseline and 9/10-weeks follow-up.  **Steps in school**  During class time, objectively measured with posture-based activPAL3 at baseline and 9/10-weeks follow-up. | **Standing desk**  The percent of total standing time increased between baseline and follow-up for the intervention (mean change [SD]=4.2% [7.4], p=0.01), however, did not significantly change in the control group (mean change [SD]=1.4% [4.4], p>0.05).  The percent of standing time during class time increased between baseline and follow-up for both intervention (mean change [SD]=8.3% [7.6], p<0.001) and control group (mean change [SD]=5.6% [6.4], p=0.001).  The percent of total stepping time did not significantly change between baseline and follow-up for both intervention (mean change [SD]=-1.1% [5.1], p>0.05) and control group (mean change [SD]=-1.3% [7.7], p>0.05).  The percent of stepping time during class time did not significantly change between baseline and follow-up for both intervention (mean change [SD]=1.1% [4.9], p>0.05) and control group (mean change [SD]=0.3% [6.2], p>0.05).  The number of total steps decreased between baseline and follow-up for both intervention (mean change [SD]=-1908 [3268], p=0.01) and control group (mean change [SD]=-2165 [4238], p=0.03).  The number of steps during class time did not significantly change between baseline and follow-up for both intervention (mean change [SD]= -143 [1245], p>0.05) and control group (mean change [SD]= -109 [1449], p>0.05). |
| Clemes et al. 2016, UK study; England | N=54; mean age: 10.0 years | Non-Randomized Intervention (duration: 9-10 weeks) | **Standing desk**  Intervention: The UK intervention class replaced 3 standard desks with 6 standing desks.  Control: Control classrooms retained their normal seated. | **Total standing time**  Waking weekday, objectively measured with posture-based activPAL3 at baseline and 9/10-weeks follow-up.  **Standing time during class time**  During class time, objectively measured with posture-based activPAL3 at baseline and 9/10-weeks follow-up.  **Total stepping time**  Waking weekday, objectively measured with posture-based activPAL3 at baseline and 9/10-weeks follow-up.  **Stepping time during class time**  During class time, objectively measured with posture-based activPAL3 at baseline and 9/10-weeks follow-up.  **Total steps**  Waking weekday, objectively measured with posture-based activPAL3 at baseline and 9/10-weeks follow-up.  **Steps in school**  During class time, objectively measured with posture-based activPAL3 at baseline and 9/10-weeks follow-up. | **Standing desk**  The percent of total standing time did not significantly change between baseline and follow-up for the intervention (mean change [SD]=1.3% [9.1], p>0.05) and the control group (mean change [SD]= -0.1% [12.1], p>0.05).  The percent of standing time during class time did not significantly change between baseline and follow-up for both intervention (mean change [SD]=3.4% [14.9], p>0.05) and control group (mean change [SD]= -2.1% [26.2], p>0.05).  The percent of total stepping time did not significantly change between baseline and follow-up for both intervention (mean change [SD]=1.1% [4.7], p>0.05) and control group (mean change [SD]=2.4% [7.4], p>0.05).  The percent of stepping time during class time increased between baseline and follow-up for the intervention group (mean change [SD]=6.3% [6.8], p=0.002), however, did not significantly change for the control group (mean change [SD]=5.4% [9.1], p>0.05).  The number of total steps did not significantly change between baseline and follow-up for both intervention (mean change [SD]=81 [4223], p>0.05) and control group (mean change [SD]=1321 [4712], p>0.05).  The number of steps during class time increased between baseline and follow-up for the intervention (mean change [SD]=1370 [1937], p=0.013), however, did not significantly change for control group (mean change [SD]=1163 [2028], p>0.05). |
| Cronholm et al. 2018; Sweden | N=228; mean age: 7.7 years | Non-Randomized Intervention (duration: 7 years) | **Recess/Physical education**  Intervention: The intervention program increased the level of PE from 60 minutes a week to 200 minutes a week, provided as daily 40-minute classes during all the compulsory school years.  Control: The control schools did the standard 60 minutes of PE. | **Organized leisure-time physical activity**  Organized leisure-time physical activity was assessed using a questionnaire at baseline and 7-year follow-up.  **Physical activity**  Physical activity was assessed using a questionnaire at baseline and 7-year follow-up.  **Screen time**  Screen time was assessed using a questionnaire at baseline and 7-year follow-up. | **Recess/Physical education**  A higher organized leisure-time PA for the intervention group, compared to the control group was observed at 7 years (mean difference in change=1.6; 95%CI: 0.4, 2.9, p=0.01).  There was no intervention effect on organized leisure-time PA observed in the intervention girls, compared to the control girls at 7 years (mean difference in change=1.2; 95%CI: -0.2, 2.6, p=0.09). A higher organized leisure-time PA for the intervention boys, compared to the control boys was observed at 7 years (mean difference in change=1.9; 95%CI: 0.0, 3.9, p=0.05).  A higher total PA for the intervention group, compared to the control group was observed at 7 years (mean difference in change=1.6; 95%CI: 0.4, 2.9, p=0.01).  There was no intervention effect on total PA observed in the intervention girls, compared to the control girls at 7 years (mean difference in change=1.2; 95%CI: -0.2, 2.6, p=0.09). A higher total PA for the intervention boys, compared to the control boys was observed at 7 years (mean difference in change=1.9; 95%CI: 0.0, 3.9, p=0.05).  There was no intervention effect on screen time observed in the intervention group, compared to the control group at 7 years (mean difference in change=-0.3; 95%CI: -3.9, 3.0, p=0.87).  A higher screen time for the intervention girls, compared to the control girls was observed at 7 years (mean difference in change=3.6; 95%CI: 0.9, 6.3, p=0.01). There was no intervention effect on screen time observed in the intervention boys, compared to the control boys at 7 years (mean difference in change=-4.5; 95%CI: 11.1, 0.9, p=0.09). |
| Galle et al. 2020; Italy | N=153; mean age: 8.7 years | Non-Randomized Intervention (duration: 6 months) | **Active breaks**  Intervention: The program consisted into two bouts of classroom activity breaks in each weekly school day, except the day including PE or any other outdoor activity. Each bout lasted 5 min and included four exercises focused on fundamental movement skills, light aerobic activity, light strength activity and gross motor coordination. Children performed exercises behind their school desk.  Control: No information is reported. Assumed to be typical classroom condition. | **Light-intensity physical activity**  Objectively measured with accelerometer (ActiGraph GT1M) at 6 months.  **Moderate‐to‐vigorous-intensity physical activity**  Objectively measured with accelerometer (ActiGraph GT1M) at 6 months.  **Physical activity**  Objectively measured with accelerometer (ActiGraph GT1M) at 6 months. | **Active breaks**  LPA was significantly higher in the intervention group (median [25^th^-75^th^ %]=36 [27-42]) compared to sitting desk condition (median [25^th^-75^th^ %]=32 [23-39]) (p=0.046) at 6 months.  LPA was significantly higher in the non-overweight intervention participants (median [25^th^-75^th^ %]=36 [31-44]) compared to non-overweight controls (median [25^th^-75^th^ %]=35 [27-40]) (p=0.012) at 6 months. There was no difference in LPA for the overweight intervention participants (median [25^th^-75^th^ %]=31 [26-42]) compared to overweight controls (median [25^th^-75^th^ %]=32 [24-41]) (p=0.778) at 6 months.  There was no difference in MVPA in the intervention group (median [25^th^-75^th^ %]=35 [26-48]) compared to sitting desk condition (median [25^th^-75^th^ %]=33 [20-46]) (p=0.248) at 6 months.  There was no difference in MVPA for the non-overweight intervention participants (median [25^th^-75^th^ %]=35 [27-46]) compared to non-overweight controls (median [25^th^-75^th^ %]=33 [24-45]) (p=0.058) at 6 months. There was no difference in MVPA for the overweight intervention participants (median [25^th^-75^th^ %]=34 [23-49]) compared to overweight controls (median [25^th^-75^th^ %]=35 [23-48]) (p=0.725) at 6 months.  Total PA was significantly higher in the intervention group (median [25^th^-75^th^ %]=73 [57-85]) compared to sitting desk condition (median [25^th^-75^th^ %]=68 [46-81]) (p=0.129) at 6 months.  Total PA was significantly higher in the non-overweight intervention participants (median [25^th^-75^th^ %]=74 [62-86]) compared to non-overweight controls (median [25^th^-75^th^ %]=69 [52-81]) (p=0.022) at 6 months. There was no difference in total PA for the overweight intervention participants (median [25^th^-75^th^ %]=71 [48-86]) compared to overweight controls (median [25^th^-75^th^ %]=69 [48-85]) (p=0.856) at 6 months. |
| Kidokoro et al. 2019; Japan | N=38; mean age: 11.3 years | Non-Randomized Intervention (duration: 6 months) | **Standing desk**  Intervention: Height-adjustable standing desks with wheels (Stafit, Okamura Co., Japan).  Control: Usual practice of using traditional seated and non-movable desks with no environmental changes. | **Light-intensity physical activity**  Objectively measured with accelerometer (ActiGraph wGT3X-BT) at baseline and 6-months follow-up.  **Moderate‐to‐vigorous-intensity physical activity**  Objectively measured with accelerometer (ActiGraph wGT3X-BT) at baseline and 6-months follow-up. | **Standing desk**  No significant changes between baseline and 6 months in LPA were found for both classes (group x time F (1, 36)=1.21, p=0.279).  MVPA in the intervention class was significantly increased between baseline and 6 months, while no change was found in the control class (group x time F (1, 36)=9.22, p=0.005). MVPA increased by 19.9 min/day in the intervention class. |
| Moller et al. 2014; Denmark | N=1,213; mean age: 9.9 years | Non-Randomized Intervention (duration: 3 years) | **Recess/Physical education**  Intervention: Children attending sports schools had a minimum of 4.5 hours **PE** per week, divided over at least 3 sessions and each session lasting at least 60 minutes.  Control: At the control schools, the children were offered the standard 2 PE lessons per week (90 minutes in total). | Objectively measured accelerometer data (Actigraph GT3X) at baseline and yearly for 3 years, was used to measure **counts per minute (CPM), sedentary time (SED), light-intensity physical activity (LPA), moderate-intensity physical activity (MPA)**, **vigorous-intensity physical activity (VPA).**  Using school timetables, accelerometer data was categorized in the domains of: **total time, school time, leisure time (i.e. all time out of school during weekdays), PE lessons, recess, and weekends.** | **Recess/Physical education**  ***CPM***  There was no difference in **total CPM** between intervention girls compared to control girls (β=-10, p=0.452), and between intervention boys compared to control boys (β=-10, p=0.452).  There was no difference in **CPM** during **school time** between intervention and control girls (β=51, p=0.065). Intervention boys had higher counts per minute during school time than control boys (β=113, p<0.001).  Intervention girls had lower **CPM** during **leisure time** than control girls (β=-41, p<0.004). Intervention boys had lower counts per minute during leisure time than control boys (β=-72, p<0.001).  There was no difference in **CPM** during **PE** between intervention girls compared to control girls (β=-15, p=0.876), and between intervention boys compared to control boys (β=-7, p=0.963).  There was no difference in **CPM** during **recess** between intervention girls compared to control girls (β=−106, p=0.228) and between intervention boys compared to control boys (β=96, p=0.232).  There was no difference in **CPM** on **weekends** between intervention girls compared to control girls (β=-32, p=0.058), and between intervention boys compared to control boys (β=-24, p=0.369).  ***SED***  Intervention girls had higher **SED** during **leisure time** than control girls (β=1.81, p=0.017). Intervention boys had higher sedentary time during leisure time than control boys (β=2.3, p=0.002).  There was no difference in **SED** on **weekends** between intervention girls compared to control girls (β=1.13, p=0.060), and between intervention boys compared to control boys (β=1.04, p=0.280).  ***LPA***  There was no difference in **total LPA** between intervention girls compared to control girls (β=−0.14, p=0.803), and between intervention boys compared to control boys (β=−0.38, p=0.458).  There was no difference in **LPA** during **school time** between intervention girls compared to control girls control girls (β=1.28, p=0.088), and between intervention boys compared to control boys (β=0.54, p=0.433).  There was no difference in **LPA** during **leisure time** between intervention girls compared to control girls (β=−0.88, p=0.156), and between intervention boys compared to control boys (β=−0.79, p=0.220).  There was no difference in **LPA** during **PE** between intervention girls compared to control girls (β=−0.64, p=0.664), and between intervention boys compared to control boys (β=−0.26, p=0.877).  There was no difference in **LPA** during **recess** between intervention girls compared to control girls (β=0.55, p=0.616), and between intervention boys compared to control boys (β=0.78, p=0.397).  There was no difference in **LPA** on **weekends** between intervention girls compared to control girls (β=− −0.69, p=0.208), and between intervention boys compared to control boys (β=−0.76, p=0.230).  ***MPA***  There was no difference in **total MPA** between intervention girls compared to control girls (β=−0.19, p=0.191), and between intervention boys compared to control boys (β=−0.22, p=0.261).  There was no difference in **MPA** during **school time** between intervention girls compared to control girls (β=0.47, p=0.063).  Intervention boys had higher MPA during school time than control boys (β=0.77, p=0.012).  Intervention girls had lower **MPA** during **leisure time** than control girls (β=−0.62, p<0.001). Intervention boys had lower MPA during leisure time than control boys (β=−0.84, p<0.001).  There was no difference in **MPA** during **PE** between intervention girls compared to control girls (β=−0.09, p=0.931), and between intervention boys compared to control boys (β=−1.04, p=0.471).  There was no difference in **MPA** during **recess** between intervention girls compared to control girls (β=−0.71, p=0.381), and between intervention boys compared to control boys (β=0.60, p=0.586).  There was no difference in **MPA** on **weekends** between intervention girls compared to control girls (β=−0.20, p=0.246), and between intervention boys compared to control boys (β=−0.19, p=0.562).  ***VPA***  There was no difference in **total VPA** between intervention girls compared to control girls (β=−0.05, p=0.572), and between intervention boys compared to control boys (β=0.06, p=0.598).  Intervention girls had higher **VPA** during **school time** than control girls (β=0.46, p=0.030). Intervention boys had higher VPA during school time than control boys (β=1.19, p <0.001).  Intervention girls had lower **VPA** during **leisure time** than control girls (β=−0.32, p=0.004). Intervention boys had lower VPA during leisure time than control boys (β=−0.53, p=0.001).  There was no difference in **VPA** during **PE** between intervention girls compared to control girls (β=−0.34, p=0.703), and between intervention boys compared to control boys (β=0.20, p=0.892).  There was no difference in **VPA** during **recess** between intervention girls compared to control girls (β=−0.83, p=0.258), and intervention boys compared to control boys (β=1.00, p=0.243).  There was no difference in **VPA** on **weekends** between intervention girls compared to control girls (β=−0.24, p=0.081), and between intervention boys compared to control boys (β=−0.10, p=0.638). |
| Sherry et al. 2020; England | N=49; mean age: 9.7 years | Non-Randomized Intervention (duration: 8 months) | **Standing desk**  Intervention: All pupils within the class received a height adjustable sit-stand desk that allows the user to manually shift between sitting and standing.  Control: The control class continued with traditional classroom furniture. | **After school sitting time**  Objectively measured with posture-based activPAL at baseline, 4 months and 8 months.  **After school standing time**  Objectively measured with posture-based activPAL at baseline, 4 months and 8 months.  **After school stepping time**  Objectively measured with posture-based activPAL at baseline, 4 months and 8 months.  **Class sit-to-stand transitions**  Objectively measured with posture-based activPAL at baseline, 4 months and 8 months.  **Class standing time**  Objectively measured with posture-based activPAL at baseline, 4 months and 8 months.  **Class stepping time**  Objectively measured with posture-based activPAL at baseline, 4 months and 8 months  **Sit-to-stand transitions**  Objectively measured with posture-based activPAL at baseline, 4 months and 8 months.  **Sitting time**  Objectively measured with posture-based activPAL at baseline, 4 months and 8 months.  **Standing time**  Objectively measured with posture-based activPAL at baseline, 4 months and 8 months.  **Stepping time**  Objectively measured with posture-based activPAL at baseline, 4 months and 8 months. | **Standing desk**  There was no difference in afterschool sitting time between intervention and control groups at 4 months (β=3.7; 95%CI: 2.50, 9.90, p=0.242) or 8 months (β=1.29; 95%CI: -5.17, 7.75, p=0.696).  There was no difference in afterschool standing time between intervention and control groups at 4 months (β=-0.25; 95%CI: -3.72, 3.22, p=0.887) or 8 months (β=3.55; 95%CI: -0.37, 7.48, p=0.076).  There was no difference in afterschool stepping time between intervention and control groups at 4 months (β=-2.3; 95%CI: -5.58, 0.98, p=0.169). A negative association between the intervention, compared to control, and after school stepping time at 8 months was observed (β=-3.73; 95%CI: -0.03, -7.43, p=0.048).  A positive association between the intervention, compared to control, and class sit-to-stand transitions was observed at 4 months (β=2.92; 95%CI: 1.33,4.51, p=0.001) and 8 months (β=4.62; 95%CI: 2.99,6.24, p=0.001).  A positive association between the intervention, compared to control, and in class standing time at 4 months (β=25.74; 95%CI: −2.28,1.75, p=0.798) and 8 months was observed (β=17.82; 95%CI: 11.88,23.76, p=0.001).  There was no difference in class stepping time between intervention and control groups at 4 months (β=−0.26; 95%CI: -5.58, 0.98, p=0.169). A positive association between the intervention, compared to control, and in class stepping time at 8 months was observed (β=-2.21; 95%CI: 0.15,4.27, p=0.035).  A positive association between the intervention, compared to control, and total sit-to-stand transitions was observed at 4 months (β=1.44; 95%CI: 0.36,2.52, p=0.009) and 8 months (β=1.36; 95%CI: 0.29,2.43, p=0.013).  A negative association between the intervention, compared to control, and total sitting time was observed at 4 months (β=−7.67; 95%CI: −12.77,−2.57, p=0.003) and 8 months (β=−5.52; 95%CI: −10.84,−0.19, p=0.042).  A positive association between the intervention, compared to control, and total standing time was observed at 4 months (β=5.78; 95%CI: 2.03,9.53, p=0.003) and 8 months (β=8.78; 95%CI: 5.16,12.40, p=0.001).  There was no difference in total stepping time between intervention and control groups at 4 months (β=−0.87; 95%CI: - −3.38,1.65, p=0.498). A negative association between the intervention, compared to control, and in class stepping time at 8 months was observed (β=-0.20; 95%CI: −2.81,2.42, p=0.883). |
| Sudholz et al. 2020; Australia | N=86; mean age: 14.8 years | Non-Randomized Intervention (duration: 17 weeks) | **Standing desk**  Intervention: A classroom was equipped with 27 height-adjustable desks  and prompts (posters and desk stickers) to break up classroom sitting time.  Control: A comparison group was recruited from adolescents in 4 classes matched by year level and subject, but whose classes were held in traditional “seated” classrooms | **Breaks from sitting**  Objectively measured with posture-based activPAL3C at baseline, 4 and 17 weeks.  **Standing time**  Objectively measured with posture-based activPAL3C at baseline, 4 and 17 weeks.  **Stepping time**  Objectively measured with posture-based activPAL3C at baseline, 4 and 17 weeks. | **Standing desk**  A higher number of breaks from sitting for the intervention group, compared to the control group, was observed at 4 weeks (mean difference in change=1.3; 95%CI: 0.3, 2.4, p=0.009) and 17 weeks (mean difference in change=1.8; 95%CI: 0.5, 3.0, p=0.004).  A higher standing time for the intervention group, compared to the control group, was observed at 4 weeks (mean difference in change=7.3; 95%CI: 2.5, 12.2, p=0.003) and 17 weeks (mean difference in change=5.8; 95%CI: 2.0,9.5, p=0.002).  There was no difference in stepping time for the intervention group, compared to the control group, at 4 weeks (mean difference in change=2.5; 95%CI: 0.7, 4.3, p=0.010) and 17 weeks (mean difference in change=1.8; 95%CI: 0.5,3.0, p=0.004). |
| Wick et al. 2018; Switzerland | N=38; mean age: 10.8 years | Non-Randomized Intervention (duration: 11 weeks) | **Standing desk**  Intervention: Teachers were asked to encourage pupils to work for about 60 min a day at the standing desks.  Control: The control class attended their lessons as usual with no restrictions regarding the activity level of the lessons. | **Break standing time**  The body position of the participants was objectively measured using the ActiGraph wGT3X-BT.  **Break walking time**  The body position of the participants was objectively measured using the ActiGraph wGT3X-BT.  **Lesson standing time**  The body position of the participants was objectively measured using the ActiGraph wGT3X-BT.  **Lesson walking time**  The body position of the participants was objectively measured using the ActiGraph wGT3X-BT. | **Standing desk**  The standing time of the intervention group was higher during the break in comparison to the control group (mean difference=−2.5, 95%CI: −4.0,−0.9, p=0.003).  The walking time in the break of the intervention group was not significantly different to the control group (mean difference=−0.9, 95%CI: −2.7,0.8, p=0.30).  The standing time of the intervention group was higher during the lesson in comparison to the control group (mean difference=−13.4, 95%CI: −22.3,−4.5, p=0.004).  The walking time in the lesson of the intervention group was not significantly different to the control group (mean difference=−1.0, 95%CI: −4.6,2.6, p=0.57). |
| Schneller et al. 2017; Denmark | N=663; mean age: 10.8 years | Non-Randomized Intervention (duration: 1 year) | **Active lessons**  Intervention: Education outside the classroom classes were offered. These are curriculum-based educational activities that are moved outside the school’s building with an average duration throughout a school year of  minimum 300 min per week.  Control: Parallel classes at the same school and grade level, based on the willingness of teachers to participate in the study. As such, approximately half the children from whom PA data were obtained attended a comparison class in which Education outside the classroom was not supposed to be a regular curriculum-based activity. All data from participating children were pooled and analysed as the amount of Education outside the classroom varied greatly between participating classes, and some control classes had practiced Education outside the classroom. Instead of comparing the original intervention and control groups we performed all analyses based on the actual exposure to Education outside the classroom by comparing days with and without Education outside the classroom, as well as the specific Education outside the classroom domain with other domains | **Education outside the classroom domain light-intensity physical activity**  Objectively measured with accelerometer (Axivity AX3).  **Education outside the classroom domain light-intensity physical activity**  Objectively measured with accelerometer (Axivity AX3).  **Physical education domain light-intensity physical activity**  Objectively measured with accelerometer (Axivity AX3).  **Physical education domain moderate‐to‐vigorous-intensity physical activity**  Objectively measured with accelerometer (Axivity AX3).  **Recess domain light-intensity physical activity**  Objectively measured with accelerometer (Axivity AX3).  **Recess domain moderate‐to‐vigorous-intensity physical activity**  Objectively measured with accelerometer (Axivity AX3).  **School day light-intensity physical activity**  Objectively measured with accelerometer (Axivity AX3).  **School day moderate‐to‐vigorous-intensity physical activity**  Objectively measured with accelerometer (Axivity AX3). | **Active lessons**  Education outside the classroom domain had a higher proportion of time in LPA for all children, compared to classroom domain (mean Education outside the classroom=56.6, mean control=49.1). Education outside the classroom domain had a higher proportion of time in LPA for girls, compared to classroom domain (mean Education outside the classroom=58.4, mean control=48.3). There was no difference for boys in proportion of time in LPA between Education outside the classroom domain classroom domain (mean Education outside the classroom=53.3, mean control=50.4).  Education outside the classroom domain had a higher proportion of time in MVPA for all children, compared to classroom domain (assumed sedentary day) (mean Education outside the classroom=10.0, mean control=6.3). Education outside the classroom domain had a higher proportion of time in MVPA for girls, compared to classroom domain (assumed sedentary day) (Mean Education outside the classroom=6.3, mean control=4.4). Education outside the classroom domain had a higher proportion of time in MVPA for boys, compared to classroom domain (assumed sedentary day) (Mean Education outside the classroom=14.9, mean control=9.4).  Education outside the classroom day had a higher proportion of time in LPA for all children, compared to school day without Education outside the classroom and PE (mean Education outside the classroom=31.0, mean control=28.7). Education outside the classroom day had a higher proportion of time in LPA for girls, compared to school day without Education outside the classroom and PE (mean Education outside the classroom=32.0, mean control=29.5). Education outside the classroom day had a higher proportion of time in LPA for boys, compared to school day without Education outside the classroom and PE (mean Education outside the classroom=29.8, mean control=27.5).  There was no difference for all children in proportion of time in MVPA between school day with Education outside the classroom and school day without Education outside the classroom and PE (mean Education outside the classroom=5.3, mean control=4.9). There was no difference for boys in proportion of time in MVPA between school day with Education outside the classroom and school day without Education outside the classroom and PE (mean Education outside the classroom=7.8, mean control=6.7). There was no difference for girls in proportion of time in MVPA between school day with Education outside the classroom and school day without Education outside the classroom and PE (mean Education outside the classroom=3.8, mean control=3.8). |
| Carlson et al. 2015; USA | N=1,322; mean age: 8.8 years | Longitudinal (duration: 5-9 months) | **Active breaks**  Minutes/day of activity breaks was obtained through survey questions.  **Recess/Physical education**  Survey questions asked whether students were provided with other PA opportunities, including recess and PE, during the day. | **Moderate‐to‐vigorous-intensity physical activity**  Objectively measured with accelerometer (ActiGraph GT3X+). | **Active breaks**  Having held physical activity breaks in the past week [y/n] was not associated with MVPA (β=-0.12; 95%CI: -1.71, 1.46, p=0.880).  Having held physical activity breaks ever [y/n] was positively associated with MVPA (β=3.14; 95%CI: 1.17, 5.12, p=0.002).  Physical activity breaks for ≥ 3 min/day was not associated with student school-day MVPA (β=1.3; 95%CI: 0.16, 2.77), p=0.81).  Time spent in physical activity breaks was positively associated with MVPA (β=0.06; 95%CI: 0.01, 0.10, p=0.016).  Time spent in physical activity breaks (excluding zero's) was positively associated with MVPA (β=0.08; 95%CI: 0.02, 0.15, p=0.012).  **Recess/Physical education**  Having ≥90 min/week of PE [y/n] was positively associated with MVPA (β=1.66; 95%CI: 0.02, 3.29, p=0.047).  Having ≥30 min/week of recess [y/n] was not associated with MVPA (β=0.74; 95%CI: 1.04, 2.52, p=0.415). |
| Maume 2017; USA | N=974; age (baseline): 12 years | Longitudinal (duration: 3 years) | **Homework**  Participants self-reported weekly hours of homework. | **Sleep**  Youth self-reported their usual bedtimes and arise times. | **Homework**  There was a negative association between homework duration at age 12 and sleep at age 15 (β=-0.08; p<0.01).  There was no association between change in homework duration from age 12 to age 15, and sleep at age 15 (β=-0.04; p>0.05). |
| Tokiya et al. 2017; Japan | N=5,687; grade 7 and 10. | Longitudinal (duration: 2 years) | **Homework**  Extracurricular learning including studying at home or at a specialized private school, after regular school hours, was assessed using a survey. | **Insomnia**  Participants reported about the following three issues: (1) difficulty initiating sleep, (2) difficulty maintaining sleep, and (3) early morning awakening. Insomnia was defined as the presence of one or more of these symptoms"). | **Homework**  There was no association between ≥2 hours/day of extracurricular learning (compared to <2 hours/day) and odds of developing insomnia, for junior high school students (Adjusted odds ratio=0.61; 95%CI: 0.27, 1.39, p=0.24).  There was a positive association between ≥2 hours/day of extracurricular learning (compared to <2 hours/day) and odds of developing insomnia, for senior high school students (Adjusted odds ratio=2.1; 95%CI: 1.23, 3.60, p=0.01). |
| Morton et al. 2016; England | N=325; mean age: 10.2 years | Cross-Sectional and Longitudinal (duration: 4 years) | **Recess/Physical education**  Hours of PE and compulsory outdoor break (yes/no) were collected using questionnaires at baseline and 4 years. | **Lunchtime light-intensity physical activity**  Objectively measured with accelerometer (Actigraph GT1M).  **Lunchtime moderate‐to‐vigorous-intensity physical activity**  Objectively measured with accelerometer (Actigraph GT1M).  **Schoolday light-intensity physical activity**  Objectively measured with accelerometer (Actigraph GT1M).  **Schoolday moderate‐to‐vigorous-intensity physical activity**  Objectively measured with accelerometer (Actigraph GT1M). | **Recess/Physical education**  No cross-sectional association between hours of PE and lunchtime LPA (β=0.001; 95%CI: -0.018, 0.019), and between compulsory outdoor break and lunchtime LPA (β=0.029; 95%CI: -0.002, 0.060).  No association between a decrease in hours of PE, compared to no change, and lunchtime LPA at 4 years (β=-1.65; 95%CI: -5.00, 1.70). No association between an increase in hours of PE, compared to no change, and lunchtime LPA at 4 years (β=-0.96; 95%CI: -3.96, 2.04). No association between going from having a rule to no outdoor rule, compared to no change, and lunchtime LPA at 4 years (β=-2.54; 95%CI: -5.44, 0.36). No association between going from no rule to having an outdoor rule, compared to no change, and lunchtime LPA at 4 years.  No cross-sectional association between hours of PE and lunchtime MVPA (β=0.015; 95%CI: -0.000, 0.030), between compulsory outdoor break and lunchtime MVPA (β=0.019; 95%CI: -0.010, 0.048).  No association between a decrease in hours of PE, compared to no change, and lunchtime MVPA at 4 years (β=-1.46; 95%CI: -4.42, 1.49). No association between an increase in hours of PE, compared to no change, and lunchtime MVPA at 4 years (β=-0.80; 95%CI: -2.01, 3.60). No association between going from having a rule to no outdoor rule, compared to no change, and lunchtime MVPA at 4 years (β=-1.63; %CI: -4.54, 1.29). No association between going from no rule to having an outdoor rule, compared to no change, and lunchtime MVPA at 4 years.  No cross-sectional association between hours of PE and schoolday LPA (β=0.004; 95%CI: -0.004, 0.012), and between compulsory outdoor break and schoolday LPA (β=0.007; 95%CI: -0.006, 0.020).  No association between a decrease in hours of PE, compared to no change, and schoolday LPA at 4 years (β=-1.41; 95%CI: -2.94, 0.11). No association between an increase in hours of PE, compared to no change, and schoolday LPA at 4 years (β=-0.56; 95%CI: -1.81, 0.70). No association between going from having a rule to no outdoor rule, compared to no change, and schoolday LPA at 4 years (β=-0.62; %CI: -1.79, 0.55). No association between going from no rule to having an outdoor rule, compared to no change, and schoolday LPA at 4 years.  No cross-sectional association between hours of PE and schoolday MVPA (β=0.006; 95%CI: -0.000, 0.001), and between compulsory outdoor break and schoolday MVPA (β=0.002; 95%CI: -0.012, 0.016).  No association between a decrease in hours of PE, compared to no change, and schoolday MVPA at 4 years (β=-0.83; 95%CI: -2.03, 0.37). No association between an increase in hours of PE, compared to no change, and schoolday MVPA at 4 years (β=-0.09; 95%CI: -1.27, 1.08). No association between going from having a rule to no outdoor rule, compared to no change, and schoolday MVPA at 4 years (β=-0.49; %CI: -1.68, 0.71). No association between going from no rule to having an outdoor rule, compared to no change, and schoolday MVPA at 4 years. |
| Patte, Qian, Leatherdale 2018; Canada | N=2,620; grade 9 to 12. | Cross-Sectional and Longitudinal (duration: 4 years) | **Homework**  Students were asked the average time per day that they spent doing homework. | **Sleep guideline adherence**  Sleep duration was assessed by asking students how much time they usually spend sleeping per day. Responses were classified as either “meets recommendations” (≥8 h) or “insufficient sleep” (< 8 h). | **Homework**  The likelihood of meeting ≥8 hours/day was lower among students who spent more time completing homework (cross-sectional associations).  Negative association between an increase in time on homework and odds of sleeping ≥8 hours/day at 4 years (Odds Ratio=0.92; 95%CI: 0.91, 0.94). |
| Brunetti et al. 2016; Canada | N=1,205; mean age: 15.2 | Cross-Sectional | **Homework**  Time spent daily doing homework and reading was measured by survey questions. | **Daytime sleepiness** was measured with the 8-item Pediatric Daytime Sleepiness Scale.  **Sleep duration** was self-reported as the number of hours of sleep per night on weekdays and on the weekends. | **Homework**  No difference in daytime sleepiness between those that do ≤2 hours (mean [SD]=10.4 [5.6]) or>2 hours of homework (mean [SD]=11.0 [6.2]) (p=0.381).  No difference in daytime sleepiness between those that do ≤2 hours or>2 hours of homework (β=0.199; 95%CI: -1.2, 1.6, p>0.05).  No difference in sleep duration between those that do ≤2 hours (mean [SD]=9.02 [0.53]) or>2 hours of homework (mean [SD]=9.04 [01.13]) (p=0.668).  No difference in sleep duration classification (short, normal, long) between those that do ≤2 hours (9.1, 81.5, 9.5, respectively) or>2 hours of homework (11.1, 76.5, 12.3, respectively) (p=.104).  No difference in sleep duration classification (short [odds ratio=1.1, 95% CI: 0.5, 2.6], normal [reference], long [odds ratio=1.4, 95% CI: 0.6, 3.1]) between those that do ≤2 hours (p>0.05). |
| Burns et al. 2015; USA | N=435; mean age: 8.4 years | Cross-Sectional | **Stationary time/sedentary time** Stationary time objectively measured with accelerometer (wGT3X-BT). | **Moderate‐to‐vigorous-intensity physical activity**  Objectively measured with accelerometer (ActiGraph wGT3X-BT). | **Stationary time/sedentary time**  The odds of meeting the MVPA recommendations were lower with increasing stationary time (odds Ratio=0.45, 95%CI: 0.34, 0.59, p=0.001). |
| Chen et al. 2014; China | N=496; mean age: 15.6 years | Cross-Sectional | **Homework**  Homework on weekdays and on weekends was assessed using a survey.  **Cram school attendance**  Questionnaires asked whether children attended cram school learning (yes/no) | **Weekday sleep duration**  Self-reported by number of hours of sleep per night  **Weekend sleep duration**  Self-reported by number of hours of sleep per night. | **Homework**  No association between homework on weekends (≥2 hours vs <2 hours) and odds of weekday short sleep duration (defined as < 8 hours) (Odds ratio=1.07; 95%CI: 0.86, 1.32, p=0.559).  More weekday homework (≥2 hours vs <2 hours) was associated with higher odds of weekday short sleep duration (defined as < 8 hours) (Odds Ratio=2.02; 95%CI: 1.70, 2.40, p=0.001).  No association between weekday homework (≥2 hours vs <2 hours) and odds of weekend short sleep duration (defined as < 8 hours) (Odds ratio=1.09; 95%CI: 0.84, 1.43, p=0.517).  No association between homework on weekends (≥2 hours vs <2 hours) and odds of weekend short sleep duration (defined as < 8 hours) (Odds Ratio=1.26; 95%CI: 0.90, 1.77, p=0.185).  Attending cram school associated with higher odds of weekend short sleep duration (<8 hours) (Odds Ratio = 1.38, 95%CI: 1.06, 1.80, p=0.018)  No association between attending cram schools and odds of weekday short sleep duration (Odds Ratio=1.08, 95%CI: 0.92, 1.27, p=0.334) |
| Ellis, Dumas, and Forbes 2020; Canada | N=1,054; mean age: 16.7 years | Cross-Sectional | **Homework**  A scale was created to measure participants’ schoolwork during the crisis. Participants were asked to think about the past 3 weeks since the COVID-19 crisis and report, on average, how much time they spent on different activities. | **Physical activity** was assessed using the Godin Leisure-Time Exercise Questionnaire. Participants were asked to report how often they did three types of exercise (strenuous, moderate, mild) for more than 15 min during the last 7 days. | **Homework**  There was a positive association between homework and physical activity (Pearson’s correlation r=0.11, p < 0.05). |
| Gu et al. 2020; USA | N=374; mean age: 9.6 years | Cross-Sectional | **Stationary time/sedentary time** Stationary time objectively measured with accelerometer (Actical). | **Light-intensity physical activity during school**  Objectively measured with Actical activity monitors.  **Moderate‐to‐vigorous-intensity physical activity during school**  Objectively measured with Actical activity monitors. | **Stationary time/sedentary time**  Negative relationship between stationary time and LPA (Pearson’s product–moment Correlation r=-0.20, p<0.01).  Negative relationship between stationary time and MVPA (Pearson’s product–moment Correlation r=-0.42, p<0.01). |
| Ishihara et al. 2018; Japan | N=325; age: 12-13 years | Cross-Sectional | **Homework** Learning duration was assessed using the time spent on learning after school on weekdays and weekends, and the use of a cram school or private teacher through the use of a questionnaire. | **Exercise habits**  Exercise habits were measured using a questionnaire about daily lifestyle behaviours, including exercise frequency in days per week (excluding PE), exercise duration on weekdays (excluding PE), and exercise duration on weekends.  **Screen time** was measured using a questionnaire about daily lifestyle behaviours. | **Homework**  No association between learning duration and exercise habits (Pearson's correlation r=0.01, p>0.05).  No association between learning duration and exercise habits in girls (Pearson's correlation r=0.06, p>0.05) and boys (Pearson's correlation r=0.00, p>0.05).  There was a positive association between learning duration and screen time (Pearson's correlation r=0.37, p<0.001).    There was a positive association between learning duration and screen time in girls (Pearson's correlation r=0.36, p<0.001) and boys (Pearson's correlation r=0.37, p<0.001). |
| Jaafar et al. 2020; Malaysia | N=603; age: 13-14 years | Cross-Sectional | **Homework**  Study time was assessed using a questionnaire. | **Physical activity status** (low, moderate and high) was assessed using the PAQ-C self-administrated, 7-day recall instrument. | **Homework**  A higher study time was associated with lower odds of physical inactivity (Odds Ratio=0.86; 95%CI: 0.75, 0.98, p=0.025). |
| Jakubec et al. 2020; Czech Republic, Poland | N=1,277; mean age: 16.5 years | Cross-Sectional | **Stationary time/sedentary time**  Physical inactivity (sedentary time) was objectively measured with accelerometer (ActiTrainerTM) in combination with the PolarTM chest strap (type S610) for heart rate monitoring, and defined as <100 CPM. Boys and girls were divided into four quartile groups according to  the ratio between physical inactivity (PI) and total PA during classes: non-sedentary, little sedentary, much sedentary, and most sedentary. | **After school energy expenditure**  Objectively measured with accelerometer (ActiTrainer).  **After school light-intensity physical activity**  Objectively measured with accelerometer (ActiTrainer) defined as <3 METs.  **After school moderate‐to‐vigorous-intensity physical activity**  Objectively measured with accelerometer (ActiTrainer) defined as ≥3 METs.  **After school physical inactivity/physical activity ratio**  Objectively measured with accelerometer (ActiTrainer).  **After school steps/hour**  Objectively measured with accelerometer (ActiTrainer).  **After school time 50%–59.9% HRmax**  Objectively measured with accelerometer (ActiTrainer) in combination with the PolarTM chest strap (type S610).  **After school time ≥60% HRmax**  Objectively measured with accelerometer (ActiTrainer) in combination with the PolarTM chest strap (type S610).  **Energy expenditure**  Objectively measured with accelerometer (ActiTrainer).  **Light-intensity physical activity**  Objectively measured with accelerometer (ActiTrainer) defined as <3 METs.  **Moderate‐to‐vigorous-intensity physical activity**  Objectively measured with accelerometer (ActiTrainer) defined as ≥3 METs.  **Physical inactivity/physical activity ratio**  Objectively measured with accelerometer (ActiTrainer).  **Recess energy expenditure**  Objectively measured with accelerometer (ActiTrainer).  **Recess light-intensity physical activity**  Objectively measured with accelerometer (ActiTrainer) defined as <3 METs.  **Recess moderate‐to‐vigorous-intensity physical activity**  Objectively measured with accelerometer (ActiTrainer) defined as ≥3 METs.  **Recess physical inactivity/physical activity ratio**  Objectively measured with accelerometer (ActiTrainer).  **Recess steps/hour**  Objectively measured with accelerometer (ActiTrainer).  **Recess time 50%–59.9% HRmax**  Objectively measured with accelerometer (ActiTrainer) in combination with the PolarTM chest strap (type S610).  **Recess time ≥60% HRmax**  Objectively measured with accelerometer (ActiTrainer) in combination with the PolarTM chest strap (type S610).  **Steps/hour**  Objectively measured with accelerometer (ActiTrainer).  **Time 50%–59.9% HRmax**  Objectively measured with accelerometer (ActiTrainer) in combination with the PolarTM chest strap (type S610).  **Time ≥60% HRmax**  Objectively measured with accelerometer (ActiTrainer) in combination with the PolarTM chest strap (type S610). | **Stationary time/sedentary time**  ***Recess***  There was no difference in energy expenditure for non-stationary (median [IQR]=0.55 [0.47]) compared to most-stationary (median [IQR]=0.47 [0.36]) girls during recess. Energy expenditure for non-stationary boys (median [IQR]=0.68 [0.56]) was higher than in most-stationary boys (median [IQR]=0.51 [0.31]) during recess.  LPA for non-stationary girls (median [IQR]=16.87 [4.19]) was higher than in most-stationary girls (median [IQR]=5.6 [2.02]) during recess. LPA for non-stationary boys (median [IQR]=19.14 [3.92]) was higher than in most-stationary boys (median [IQR]=7.33 [2.27]) during recess.  There was no difference in MVPA for non-stationary (median [IQR]=2.1 [2.28]) compared to most-stationary (median [IQR]=0.11 [0.38]) girls during recess. There was no difference in MVPA for non-stationary (median [IQR]=2.00 [2.82]) compared to most-stationary (median [IQR]=0.25 [0.56]) boys during recess.  There was no difference in number of steps/hour for non-stationary (median [IQR]=889 [746]) compared to most-stationary (median [IQR]=920 [613]) girls during recess. The number of steps/hour was higher for non-stationary (median [IQR]=1066 [661]) compared to most-stationary (median [IQR]=839 [482]) boys during recess.  There was no difference in time in 50-59.9% HRmax for non-stationary (median [IQR]=10.67 [12.25]) compared to most-stationary (median [IQR]=8.36 [11]) girls during recess. There was no difference in time in 50-59.9% HRmax for non-stationary (median [IQR]=9.57 [14.19]) compared to most-stationary (median [IQR]=7.00 [12.29]) boys during recess.  There were no differences in time in ≥60% HRmax for non-stationary (median [IQR]=1.29 [5.00]) compared to most-stationary (median [IQR]=0.75 [2.5]) girls during recess. There were no differences in time in ≥60% HRmax for non-stationary (median [IQR]=0.59 [4.73]) compared to most-stationary (median [IQR]=0.00 [1.5]) boys during recess.  ***After school***  There was no difference in energy expenditure for non-stationary (median [IQR]=0.47 [0.43]) compared to most-stationary (median [IQR]=0.54 [0.36]) girls after school. There was no difference in energy expenditure for non-stationary (median [IQR]=0.65 [0.64]) compared to most-stationary (median [IQR]=0.49 [0.43]) boys after school.  There was no difference in LPA for non-stationary (median [IQR]=18.86 [9.70]) compared to most-stationary (median [IQR]=17.88 [6.97]) girls after school. LPA for non-stationary boys (median [IQR]=18.67 [9.80]) was higher than in most-stationary boys (median [IQR]=15.11 [7.47]) after school.  There was no difference in MVPA for non-stationary (median [IQR]=3.52 [4.40]) compared to most-stationary (median [IQR]=4.07 [3.62]) girls after school. There was no difference in MVPA for non-stationary (median [IQR]=4.53 [6.04]) compared to most-stationary (median [IQR]=3.86 [3.88]) boys after school.  There was no difference in number of steps/hour for non-stationary (median [IQR]=717 [574]) compared to most-stationary (median [IQR]=804 [499]) girls after school. There was no difference in number of steps/hour for non-stationary (median [IQR]=814 [761]) compared to most-stationary (median [IQR]=662 [489]) boys after school.  There was no difference in time in 50-59.9% HRmax for non-stationary (median [IQR]=7.78 [9.31]) compared to most-stationary (median [IQR]=7.35 [7.70]) girls after school. There was no difference in time in 50-59.9% HRmax for non-stationary (median [IQR]=7.71 [10.06]) compared to most-stationary (median [IQR]=5.90 [8.81]) boys after school.  There were no differences in time in ≥60% HRmax for non-stationary (median [IQR]=2.24 [5.05]) compared to most-stationary (median [IQR]=2.36 [4.83]) girls after school. There were no differences in time in ≥60% HRmax for non-stationary (median [IQR]=2.12 [11.05]) compared to most-stationary (median [IQR]=1.17 [5.59]) boys after school.  ***Whole day***  Energy expenditure for non-stationary girls (median [IQR]=0.46 [0.26]) was higher compared to most-stationary (median [IQR]=0.39 [0.19]) girls. Energy expenditure for non-stationary boys (median [IQR]=0.57 [0.42]) was higher compared to most-stationary (median [IQR]=0.40 [0.23]) boys.  LPA was higher in non-stationary (median [IQR]=20.92 [6.30]) compared to most-stationary (median [IQR]=16.51 [4.15]) girls. LPA was higher in non-stationary boys (median [IQR]=21.84 [5.78]) compared to most-stationary boys (median [IQR]=16.21 [4.21]).  There was no difference in MVPA for non-stationary (median [IQR]=4.56 [3.06]) compared to most-stationary (median [IQR]=3.82 [2.84]) girls. There was no difference in MVPA for non-stationary (median [IQR]=5.48 [4.16]) compared to most-stationary (median [IQR]=4.83 [3.70]) boys.  The number of steps/hour for non-stationary girls (median [IQR]=709 [338]) was higher compared to most-stationary girls (median [IQR]=605 [251]). The number of steps/hour for non-stationary boys (median [IQR]=798 [422]) was higher compared to most-stationary boys (median [IQR]=591 [286]).  There was no difference in time in 50-59.9% HRmax for non-stationary (median [IQR]=11.20 [7.33]) compared to most-stationary (median [IQR]=9.52 [6.44]) girls. There was no difference in time in 50-59.9% HRmax for non-stationary (median [IQR]=9.17 [8.02]) compared to most-stationary (median [IQR]=8.24 [6.21]) boys.  There were no differences in time in ≥60% HRmax for non-stationary (median [IQR]=4.34 [5.98]) compared to most-stationary (median [IQR]=3.35 [4.16]) girls. There were no differences in time in ≥60% HRmax for non-stationary (median [IQR]=3.94 [7.11]) compared to most-stationary (median [IQR]=8.24 [6.21]) boys.  Boys in the more-sedentary (Odds ratio=2.00; 95%CI: 1.10-3.65, p=0.023), less-sedentary (Odds ratio=1.77; 95%CI: 0.97-3.25, p=0.063) and non-sedentary (Odds ratio=3.62; 95%CI: 1.98-6.63, p<0.001) group had higher odds of meeting ≥60 minutes of MVPA/day, compared to those in the most-sedentary group (reference).  There was no difference in odds of meeting ≥60 minutes of MVPA/day between girls in the more-sedentary (Odds ratio=1.41; 95%CI: 0.92-2.15, p=0.115) and less-sedentary (Odds ratio=1.35; 95%CI: 0.88-2.06, p=0.172) group compared to those in the most-sedentary group (reference). Girls in the non-sedentary (Odds ratio=1.82; 95%CI: 1.20-2.76, p=0.005) group had higher odds of meeting ≥60 minutes of MVPA/day, compared to those in the most-sedentary group (reference).  There was no difference in odds of meeting 11,000 steps/day between boys in the more-sedentary (Odds ratio=1.35; 95%CI: 0.73-2.50, p=0.335) and less-sedentary (Odds ratio=1.59; 95%CI: 0.86-2.91, p=0.137) group compared to those in the most-sedentary group (reference). Boys in the non-sedentary (Odds ratio=2.38; 95%CI: 1.30-4.34, p<0.001) group had higher odds of meeting 11,000 steps/day, compared to those in the most-sedentary group (reference).  There was no difference in odds of meeting 11,000 steps/day between girls in the more-sedentary (Odds ratio=1.46; 95%CI: 0.96-2.12, p=0.078) and less-sedentary (Odds ratio=1.39; 95%CI: 0.92-2.12, p=0.123) group compared to those in the most-sedentary group (reference). Girls in the non-sedentary (Odds ratio=2.47; 95%CI: 1.64-3.71, p<0.001) group had higher odds of meeting 11,000 steps/day, compared to those in the most-sedentary group (reference). |
| Jiang et al. 2015; China | N=6,247; mean age: 11.5 years | Cross-Sectional | **Homework**  Time spent on homework on an average school day was measured by a questionnaire. | **Daytime tiredness** was assessed using the Multidimensional Sub-health Questionnaire of Adolescents.  **Difficulty initiating sleep** was assessed using the Multidimensional Sub-health Questionnaire of Adolescents.  **Difficulty maintaining sleep** was assessed using the Multidimensional Sub-health Questionnaire of Adolescents. | **Homework**  No association between homework time and odds of daytime tiredness, for elementary school-aged children (Odds ratio=1.07, 95%CI: 0.97, 1.17). Positive association between homework time and odds of daytime tiredness, for secondary school-aged children (Odds ratio=1.16, 95%CI: 1.07, 1.27).  No association between homework time and odds of difficulties initiating sleep, for elementary school-aged children (Odds ratio=0.98, 95%CI: 0.88, 1.08). No association between homework time and odds of difficulties initiating sleep, for secondary school-aged children (Odds ratio=1.05, 95%CI: 0.95, 1.17).  No association between homework time and odds of difficulties maintaining sleep, for elementary school-aged children (Odds ratio=0.98, 95%CI: 0.87, 1.09). Positive association between homework time and odds of difficulties maintaining sleep, for secondary school-aged children (Odds ratio=1.18, 95%CI: 1.07, 1.31). |
| Li et al. 2014; China | N=2,379; age: 9 years | Cross-Sectional | **Homework**  General homework schedules were evaluated for weekday and weekend day homework time. | **Weekday bedtime** was assessed using a parent-administered questionnaire regarding children’s sleep habits.    **Weekday sleep duration** was assessed using a parent-administered questionnaire regarding children’s sleep habits (bedtime and wake time in the morning).  **Weekday wake time** was assessed using a parent-administered questionnaire regarding children’s sleep habits.    **Weekend bedtime** was assessed using a parent-administered questionnaire regarding children’s sleep habits.    **Weekend sleep duration** was assessed using a parent-administered questionnaire regarding children’s sleep habits (bedtime and wake time in the morning).  **Weekend wake time** was assessed using a parent-administered questionnaire regarding children’s sleep habits. | **Homework**  Positive association between homework during weekdays and bedtime on weekdays (β [SE]=0.189 [0.007], p<0.001). No association between homework during weekends and bedtime on weekdays (β [SE]=0.009 [0.006], p=0.065).  There was a positive trend for homework duration during weekdays and odds of highest quantile of bedtime on weekdays (Odds Ratio=1.58, 95%CI: 1.50, 1.67, p<0.001). Doing homework during weekdays for ≥3 hours (Odds ratio=4.28; 95%CI: 3.46, 5.31, p<0.001), 2-3 hours (Odds ratio=2.29; 95%CI: 2.02, 2.60, p<0.001) or 1-2 hours (Odds ratio=1.36; 95%CI: 1.23, 1.50, p<0.001) was associated with higher odds of being classified in the highest quantile of bedtime on weekdays, compared to <1 hour (reference).  There was no significant trend for homework duration during weekend days and odds of highest quantile of bedtime on weekdays (Odds Ratio=1.00, 95%CI: 0.95, 1.05, p=0.978). Doing homework during weekend days for ≥3 hours (Odds ratio=1.02; 95%CI: 0.87,1.20, p=0.812), 2-3 hours (Odds ratio=1.02; 95%CI: 0.89, 1.17, p=0.77) or 1-2 hours (Odds ratio=1.01; 95%CI: 0.89, 1.14, p=0.932) was not significantly associated with higher odds of being classified in the highest quantile of bedtime on weekdays, compared to <1 hour (reference).  Negative association between homework during weekdays and sleep duration on weekdays (β [SE]=-0.18 [0.008], p<0.001). No association between homework during weekends and sleep duration on weekdays (β [SE]=-0.007 [0.035], p=0.124).  There was a positive trend for homework duration during weekdays and odds of highest quantile of sleep duration on weekdays (Odds Ratio=1.56, 95%CI: 1.46, 1.67, p<0.001). Doing homework during weekdays for ≥3 hours (Odds ratio=3.62; 95%CI: 2.87, 4.56, p<0.001), 2-3 hours (Odds ratio=1.91; 95%CI: 1.62, 2.26, p<0.001), but not 1-2 hours (Odds ratio=1.11; 95%CI: 0.96, 1.28, p=0.145), was associated with higher odds of being classified in the highest quantile of sleep duration on weekdays, compared to <1 hour (reference).  There was no significant trend for homework duration during weekend days and odds of highest quantile of sleep duration on weekdays (Odds Ratio=1.01, 95%CI: 0.95, 1.08, p=0.69). Doing homework during weekend days for ≥3 hours (Odds ratio=1.09; 95%CI: 0.88, 1.34, p=0.442), 2-3 hours (Odds ratio=0.97; 95%CI: 0.81, 1.17, p=0.748), and 1-2 hours (Odds ratio=0.97; 95%CI: 0.82, 1.16, p=0.768), was not associated with higher odds of being classified in the highest quantile of sleep duration on weekdays, compared to <1 hour (reference).  No association between homework during weekdays and wake time on weekdays (β [SE]=0.002 [0.005], p=0.748). No association between homework during weekends and wake time on weekdays (β [SE]=0.01 [0.004], p=0.054).  There was a positive trend for homework duration during weekdays and odds of highest quantile of wake time on weekdays (Odds Ratio=1.16, 95%CI: 1.10, 1.23, p<0.001). Doing homework during weekdays for ≥3 hours (Odds ratio=1.61; 95%CI: 1.31, 1.97, p<0.001), 2-3 hours (Odds ratio=1.29; 95%CI: 1.13, 1.46, p<0.001), but not 1-2 hours (Odds ratio=1.07; 95%CI: 0.97, 1.19, p=0.184), was associated with higher odds of being classified in the highest quantile of bedtime on weekdays, compared to <1 hour (reference).  There was a positive trend for homework duration during weekdays and odds of highest quantile of wake time on weekdays (Odds Ratio=1.12, 95%CI: 1.01, 1.24, p=0.0021). Doing homework during weekdays for ≥3 hours (Odds ratio=1.46.; 95%CI: 1.08, 1.67, p<0.001), 2-3 hours (Odds ratio=1.14; 95%CI: 1.03, 1.26, p=0.025), but not 1-2 hours (Odds ratio=1.12; 95%CI: 0.96, 1.29, p=0.256), was associated with higher odds of being classified in the highest quantile of bedtime on weekdays, compared to <1 hour (reference).  Positive association between homework during weekends and bedtime on weekends (β [SE]=0.054 [0.007], p<0.001). Positive association between homework during weekdays and bedtime on weekends (β [SE]=0.1 [0.008], p<0.001).  There was a positive trend for homework duration during weekdays and odds of highest quantile of bedtime on weekend days (Odds Ratio=1.23, 95%CI: 1.17, 1.31, p<0.001). Doing homework during weekdays for ≥3 hours (Odds ratio=1.68; 95%CI: 1.36, 2.07, p<0.001), 2-3 hours (Odds ratio=1.52; 95%CI: 1.34, 1.74, p<0.001) or 1-2 hours (Odds ratio=1.12; 95%CI: 1.00, 1.24, p=0.046) was associated with higher odds of being classified in the highest quantile of bedtime on weekend days, compared to <1 hour (reference).  There was a positive trend for homework duration during weekend days and odds of highest quantile of bedtime on weekend days (Odds Ratio=1.13, 95%CI: 1.08, 1.23, p=0.007). Doing homework during weekdays for ≥3 hours (Odds ratio=1.52; 95%CI: 1.11, 1.67, p<0.001) and 2-3 hours (Odds ratio=1.19; 95%CI: 1.10, 1.36, p<0.001), but not 1-2 hours (Odds ratio=1.05; 95%CI: 0.92, 1.17, p=0.124), was associated with higher odds of being classified in the highest quantile of bedtime on weekend days, compared to <1 hour (reference).  No association between homework during weekdays and sleep duration on weekends (β [SE]=-0.008 [0.011], p=0.068). Negative association between homework during weekends and sleep duration on weekends (β [SE]=-0.045 [0.010], p<0.001).  There was no significant trend for homework duration during weekdays and odds of highest quantile of sleep duration on weekend days (Odds Ratio=1.08, 95%CI: 0.99, 1.10, p=0.066). Doing homework during weekdays for ≥3 hours (Odds ratio=1.2; 95%CI: 1.00, 1.59, p=0.053), 2-3 hours (Odds ratio=1.11; 95%CI: 0.98, 1.27, p=0.065), and 1-2 hours (Odds ratio=1.07; 95%CI: 0.97, 1.19, p=0.165), was not associated with higher odds of being classified in the highest quantile of sleep duration on weekend days, compared to <1 hour (reference).  There was a positive trend for homework duration during weekend days and odds of highest quantile of sleep duration on weekend days (Odds Ratio=1.17, 95%CI: 1.11, 1.25, p<0.001). Doing homework during weekend days for ≥3 hours (Odds ratio=1.64; 95%CI: 1.33, 2.00, p<0.001), 2-3 hours (Odds ratio=1.29; 95%CI: 1.13, 1.47, p<0.001), but not 1-2 hours (Odds ratio=1.02; 95%CI: 0.90, 1.13, p=0.274), was associated with higher odds of being classified in the highest quantile of sleep duration on weekend days, compared to <1 hour (reference).  Positive association between homework during weekdays and wake time on weekends (β [SE]=0.072 [0.010], p<0.001). Positive association between homework during weekends and wake time on weekends (β [SE]=0.035 [0.009], p<0.001). |
| Lushington et al. 2015; Australia | N=398; age: 17.0 years | Cross-Sectional | **Homework**  An omnibus questionnaire assessed several items including homework time. | **Daytime sleepiness** was assessed using a self-reported survey assessing sleep and extracurricular activity based on items developed by Acebo and Carskadon and modified for use in an Australian population.  **Sleep period** was assessed using a self-reported survey assessing sleep and extracurricular activity based on items developed by Acebo and Carskadon and modified for use in an Australian population. | **Homework**  There was no association between homework time and daytime sleepiness, in Asian-Australians (p>0.05) and in Caucasian-Australians (p>0.05)  There was no association between homework time and sleep period, in Asian-Australians (p<0.05) and in Caucasian-Australians (p>0.05). |
| Martinez-Lopez et al. 2015; Spain | N=2,293; mean age: 14.2 years | Cross-Sectional | **Homework**  Homework on weekends and weekdays was assessed using a questionnaire. | **Computer on weekdays** Participants self-reported the number of hours a day that they spent on the PC.  **Computer on weekends** Participants self-reported the number of hours a day that they spent on the PC.  **Physical activity**  Participants self-reported the time spent in MVPA.  **Television on weekdays** Participants self-reported the number of hours a day that they watch TV.  **Television on weekends** Participants self-reported the number of hours a day that they watch TV. | **Homework**  No association between PC on weekdays and homework on weekdays for girls (Pearson's correlation r=0.030, p>0.05). Positive association between PC on weekdays and homework on weekdays for boys (Pearson's correlation r=0.069, p<0.05).  Positive association between PC on weekdays and homework on weekends for girls (Pearson's correlation r=0.604, p<0.01). Positive association between PC on weekdays and homework on weekends for boys (Pearson's correlation r=0.596, p<0.01).  Positive association between PC on weekends and homework on weekdays for girls (Pearson's correlation r=0.224, p<0.01). Positive association between PC on weekends and homework on weekdays for boys (Pearson's correlation r=0.181, p<0.01).  Positive association between PC on weekends and homework on weekends for girls (Pearson's correlation r=0.164, p<0.01). Positive association between PC on weekends and homework on weekends for boys (Pearson's correlation r=0.126, p<0.01).  No association between physical activity and homework on weekdays for girls (Pearson's correlation r=-0.030, p>0.05). No association between physical activity and homework on weekdays for boys (Pearson's correlation r=-0.003, p>0.05).  No association between physical activity and homework on weekend days for girls (Pearson's correlation r=-0.036, p>0.05). No association between physical activity and homework on weekend days for boys (Pearson's correlation r=0.001, p>0.05).  Positive association between TV on weekdays and homework on weekdays for girls (Pearson's correlation r=0.089, p<0.01). Positive association between TV on weekdays and homework on weekdays for boys (Pearson's correlation r=0.079, p<0.01.  Negative association between TV on weekdays and homework on weekends for girls (Pearson's correlation r=-0.088, p<0.01). No association between TV on weekdays and homework on weekends for boys (Pearson's correlation r=-0.017, p>0.05).  Positive association between TV on weekends and homework on weekdays for girls (Pearson's correlation r=0.576, p>0.01). Positive association between TV on weekends and homework on weekdays for boys (Pearson's correlation r=0.615, p>0.01).  Negative association between TV on weekends and homework on weekends for girls (Pearson's correlation r=-0.115, p>0.01). No association between TV on weekends and homework on weekends for boys (Pearson's correlation r=-0.055, p>0.05). |
| Michaud et al. 2015; Canada | N=511; mean age: 9.6 years | Cross-Sectional | **Homework**  Homework duration on weekdays and weekend were collected using a questionnaire. | **Moderate‐to‐vigorous-intensity physical activity**  Objectively measured with accelerometer (Actigraph LS 7164 and CAL71 calibrator).  **Screen time**  Daily screen time was self-reported.  **Sleep**  Sleep duration was calculated as the mean difference between the time at which the accelerometer was removed before bedtime and put back on the following morning. | **Homework**  There was no difference in MVPA between doing homework for <30 min/day (Median [IQR]=17.8 [11.0, 27.7]) and ≥30 min/day (Median [IQR]=16.0 [10.1, 23.0]) for girls (p=0.078). There was no difference in MVPA between doing homework for <30 min/day (Median [IQR]=18.3 [11.3, 29.8]) and ≥30 min/day (Median [IQR]=16.6 [11.2, 23.0]) for girls not stressed about schoolwork (p=0.185). There was no difference in MVPA between doing homework for <30 min/day (Median [IQR]=17.3 [10.9, 26.4]) and ≥30 min/day (Median [IQR]=15.4 [9.3, 23.4]) for girls stressed about schoolwork (p=0.147)  Doing homework for <30 min/day (Median [IQR]=30.7 [20.0, 48.6]) was associated with lower MVPA compared to ≥30 min/day (Median [IQR]=21.2 [12.8, 36.6]) for boys (p=0.010). There was no difference in MVPA between doing homework for <30 min/day (Median [IQR]=29.2 [20.2, 43.5]) and ≥30 min/day (Median [IQR]=27.0 [20.3, 37.3]) for boys not stressed about schoolwork (p=0.132). Doing homework for <30 min/day (Median [IQR]=29.4 [20.0, 45.8]) was associated with lower MVPA compared to ≥30 min/day (Median [IQR]=26.1 [13.7, 37.3]) for boys stressed about schoolwork (p=0.005)  Doing homework for <30 min/day (Median [IQR]=1.7 [1.1, 2.4]) was associated with higher screen time compared to ≥30 min/day (Median [IQR]=2.1 [1.7, 3.7]) for girls (p=0.010). There was no difference in screen time between doing homework for <30 min/day (Median [IQR]=1.9 [1.1, 2.7]) and ≥30 min/day (Median [IQR]=1.8 [1.1, 3.4]) for girls stressed about schoolwork (p=0.392). Doing homework for <30 min/day (Median [IQR]=1.5 [0.8, 2.2]) was associated with higher screen time compared to ≥30 min/day (Median [IQR]=2.3 [1.2, 3.9]) for girls not stressed about schoolwork (p=0.002).  There was no difference in screen time between doing homework for <30 min/day (Median [IQR]=2.5 [1.6, 4.1]) and ≥30 min/day (Median [IQR]=2.6 [1.6, 3.8]) for boys (p=0.476). There was no difference in screen time between doing homework for <30 min/day (Median [IQR]=2.3 [1.6, 4.1]) and ≥30 min/day (Median [IQR]=2.6 [1.9, 3.8]) for boys not stressed about schoolwork (p=0.297). There was no difference in screen time between doing homework for <30 min/day (Median [IQR]=3.1 [1.6, 4.4]) and ≥30 min/day (Median [IQR]=2.6 [1.5, 4.0]) for boys stressed about schoolwork (p=0.354)  There was no difference in sleep between doing homework for <30 min/day (Median [IQR]=10.6 [10.2, 11.2]) and ≥30 min/day (Median [IQR]=10.4 [10.0, 11.0]) for girls (p=0.068). There was no difference in sleep between doing homework for <30 min/day (Median [IQR]=10.5 [10.2, 11.0]) and ≥30 min/day (Median [IQR]=10.4 [10.0, 11.0]) for girls not stressed about schoolwork (p=0.211). There was no difference in sleep between doing homework for <30 min/day (Median [IQR]=10.7 [10.1, 11.2]) and ≥30 min/day (Median [IQR]=10.4 [10.0, 11.0]) for girls stressed about schoolwork (p=0. 097).  There was no difference in sleep between doing homework for <30 min/day (Median [IQR]=10.4 [10.0, 10.9]) and ≥30 min/day (Median [IQR]=10.4 [10.0, 11.3]) for boys (p=0.187). There was no difference in sleep between doing homework for <30 min/day (Median [IQR]=10.4 [10.1, 11.1]) and ≥30 min/day (Median [IQR]=10.4 [10.0, 11.2]) for boys not stressed about schoolwork (p=0.448). There was no difference in sleep between doing homework for <30 min/day (Median [IQR]=10.2 [9.9, 10.8]) and ≥30 min/day (Median [IQR]=10.5 [10.0, 11.5]) for boys stressed about schoolwork (p=0.054). |
| Peiro-Velert et al. 2014; Spain | N=3,095; age: 12-18 years | Cross-Sectional | **Homework**  Computer use for doing homework was assessed using a questionnaire. | **Active videogames**  Active videogames was self-reported using a questionnaire.  **Computer communicating** Computer for communicating was self-reported using a questionnaire.  **Computer playing**  Computer for playing time was self-reported using a questionnaire.  **Mobile communicating**  Mobile for communicating was self-reported using a questionnaire.  **Mobile playing**  Mobile for playing time was self-reported using a questionnaire.  **Passive videogames**  Time spent playing passive videogames was self-reported using a questionnaire.  **Sedentary screen media usage**  Overall sedentary screen time was the sum of three self-reported items: TV/video/DVD viewing, overall computer use, and passive videogames.  **Sleep time**  Sleep hours, including a siesta or nap were self-reported using a questionnaire.  **Television/video/** **Digital Video Disc time**  Time spent watching TV, videos and DVDs was self-reported using a questionnaire. | **Homework**  No association between time spent using a computer for homework and time spent playing active videogames (Spearman's correlation r=-0.07; 95%CI: -0.20, 0.06).  Positive association between time spent using a computer for homework and time spent using a computer for communicating (Spearman's correlation r=0.91; 95%CI: 0.88, 0.93).  Positive association between time spent using a computer for homework and time spent computer playing (Spearman's correlation r=0.22; 95%CI: 0.09, 0.34).  Positive association between time spent using a computer for homework and time spent using a mobile for communicating (Spearman's correlation r=0.56; 95%CI: 0.46, 0.64).  Positive association between time spent using a computer for homework and time spent using a mobile for playing (Spearman's correlation r=0.18; 95%CI: 0.05, 0.31).  No association between time spent using a computer for homework and time spent playing passive videogames (Spearman's correlation r=-0.07; 95%CI: -0.20, 0.06).  Positive association between time spent using a computer for homework and time spent on overall sedentary screen media usage (Spearman's correlation r=0.68; 95%CI: 0.60, 0.75).  Negative association between time spent using a computer for homework and sleep time (Spearman's correlation r=-0.38; 95%CI: -0.49, -0.26).  Positive association between time spent using a computer for homework and time spent watching TV/video/DVD (Spearman's correlation r=0.47; 95%CI: 0.36, 0.57). |
| Schneller et al. 2017; Denmark | N=663; mean age: 10.8 years | Cross-sectional | **Recess/physical Education**  Participant’s time spent in recess, PE, education outside the classroom, and typical class time was measured using class timetables, class diaries and individual leisure time diaries.  Days with or without recess, PE, education outside the classroom, and typical class time was measured using class timetables, class diaries and the online EOtC monitoring tool. Class diaries were filled in by three children selected by the class teacher in cooperation with the teacher. | **Light-intensity physical activity**  Objectively measured with accelerometer (Axivity AX3).  **Moderate‐to‐vigorous-intensity physical activity**  Objectively measured with accelerometer (Axivity AX3). | **Recess/Physical education**  PE domain had a higher proportion of time in LPA for all children, compared to classroom domain (mean PE=57.9, mean control=49.1). PE domain had a higher proportion of time in LPA for girls, compared to classroom domain (mean PE=60.4, mean control=48.3). PE domain had a higher proportion of time in LPA for boys, compared to classroom domain (mean PE=53.6, mean control=50.4).  PE domain had a higher proportion of time in MVPA for all children, compared to classroom domain (mean PE=25.7, mean control=6.3). PE domain had a higher proportion of time in MVPA for girls, compared to classroom domain (mean PE=22.3, mean control=4.4). PE domain had a higher proportion of time in MVPA for boys, compared to classroom domain (mean PE=31.0, mean control=9.4).  Recess domain had a higher proportion of time in LPA for all children, compared to classroom domain (mean recess=55.1, mean control=49.1). Recess domain had a higher proportion of time in LPA for girls, compared to classroom domain (mean recess=59.4, mean control=48.3). Recess domain had a higher proportion of time in LPA for boys, compared to classroom domain (mean recess=47.6, mean control=50.4).  Recess domain had a higher proportion of time in MVPA for all children, compared to classroom domain (mean PE=20.0, mean control=6.3). Recess domain had a higher proportion of time in MVPA for girls, compared to classroom domain (mean PE=13.9, mean control=4.4). Recess domain had a higher proportion of time in MVPA for boys, compared to classroom domain (mean PE=29.9, mean control=9.4).  There was no difference for all children in proportion of time in LPA between school day with PE and school day without Education outside the classroom and PE (mean PE=28.7, mean control=28.7). There was no difference for girls in proportion of time in LPA between school day with PE and school day without Education outside the classroom and PE (mean PE=29.8, mean control=29.5). There was no difference for boys in proportion of time in LPA between school day with PE and school day without Education outside the classroom and PE (mean PE=27.5, mean control=27.5).  PE day had a higher proportion of time in MVPA for all children, compared to school day without Education outside the classroom and PE (mean PE=5.9, mean control=4.9). PE day had a higher proportion of time in MVPA for girls, compared to school day without Education outside the classroom and PE (mean PE=4.6, mean control=3.8). PE day had a higher proportion of time in MVPA for boys, compared to school day without Education outside the classroom and PE (mean PE=8.5, mean control=6.7). |
| Sharma, Chavez, and Nam 2018; Peru | N=1,354; age: 11 to 19 years | Cross-Sectional | **Recess/Physical education**  Survey included an item asking how many days per week PE class was attended at school. | **Physical activity**  Physical activity was measured using a single self-reported question.  . | **Recess/Physical education**  Adolescents who attended five PE classes each week were less likely to report insufficient PA than those who mentioned less than five physical education classes (adjusted prevalence ratio=0.94; 95%CI: 0.88-0.99, p<0.05). |
| Sigmund et al. 2014; Czech Republic | N=338; mean age: 9.9 years | Cross-Sectional | **Recess/ Physical education**  Stationary time and PA were objectively measured with accelerometer (ActiTrainerTM). Under the supervision of their class teachers, the participating children further recorded the beginning times of PE school lessons and recesses in the individual PA log book. | **After-school light-intensity physical activity**  Objectively measured with accelerometer (ActiTrainer).  **After-school moderate‐to‐vigorous-intensity physical activity**  Objectively measured with accelerometer (ActiTrainer).  **After-school stationary time**  Objectively measured with accelerometer (ActiTrainer).  **Before school light-intensity physical activity**  Objectively measured with accelerometer (ActiTrainer).  **Before school moderate‐to‐vigorous-intensity physical activity**  Objectively measured with accelerometer (ActiTrainer).  **Before school stationary time**  Objectively measured with accelerometer (ActiTrainer).  **Light-intensity physical activity**  Objectively measured with accelerometer (ActiTrainer).  **Moderate‐to‐vigorous-intensity physical activity**  Objectively measured with accelerometer (ActiTrainer).  **School light-intensity physical activity**  Objectively measured with accelerometer (ActiTrainer).  **School moderate‐to‐vigorous-intensity physical activity**  Objectively measured with accelerometer (ActiTrainer). | **Recess/Physical education**  No difference in after-school LPA between days with and without PE, for normal weight girls (Median PE=212.05, Median no PE=223.51, p>0.05) and for normal weight boys (Median PE=191.28, Median no PE=186.99, p>0.05). No difference in after-school LPA between days with and without PE, for overweight girls (Median PE=202.74, Median no PE=207.98, p>0.05) and for overweight boys (Median PE=173.33, Median no PE=182.11, p>0.05).  No difference in after-school MVPA between days with and without PE, for normal weight girls (Median PE=26.02, Median no PE=27.06, p>0.05) and for normal weight boys (Median PE=35.17, Median no PE=30.39, p>0.05). No difference in after-school MVPA between days with and without PE, for overweight girls (Median PE=27.49, Median no PE=24.15, p>0.05) and for overweight boys (Median PE=16.25, Median no PE=18.17, p>0.05).  No difference in after-school stationary time between days with and without PE, for normal weight girls (Median PE=275.03, Median no PE=317.90, p>0.05) and for normal weight boys (Median PE=282.62, Median no PE=324.53, p>0.05). No difference in after-school stationary time between days with and without PE, for overweight girls (Median PE=288.50, Median no PE=325.81, p>0.05) and for overweight boys (Median PE=314.99, Median no PE=364.18, p>0.05).  No difference in before school LPA between days with and without PE, for normal weight girls (Median PE=20.36, Median no PE=27.75, p>0.05) and for normal weight boys (Median PE=21.75, Median no PE=25.1, p>0.05). No difference in before school LPA between days with and without PE, for overweight girls (Median PE=21.65, Median no PE=24.25, p>0.05) and for overweight boys (Median PE=26.5, Median no PE=34.25, p>0.05).  No difference in before school MVPA between days with and without PE, for normal weight girls (Median PE=1.12, Median no PE=1.78, p>0.05) and for normal weight boys (Median PE=2, Median no PE=1.99, p>0.05). No difference in before school MVPA between days with and without PE, for overweight girls (Median PE=2.78, Median no PE=1.5, p>0.05) and for overweight boys (Median PE=2, Median no PE=2.6, p>0.05).  No difference in before school stationary time between days with and without PE, for normal weight girls (Median PE=13.87, Median no PE=13.39, p>0.05) and for normal weight boys (Median PE=16.66, Median no PE=16.5, p>0.05). No difference in before school stationary time between days with and without PE, for overweight girls (Median PE=13.39, Median no PE=12.75, p>0.05) and for overweight boys (Median PE=13, Median no PE=15.5, p>0.05).  No difference in LPA between days with and without PE, for normal weight girls (Median PE=358.62, Median no PE=360.92, p>0.05) and for normal weight boys (Median PE=342.6, Median no PE=335.8, p>0.05). No difference in LPA between days with and without PE, for overweight girls (Median PE=352.87, Median no PE=361.38, p>0.05) and for overweight boys (Median PE=324.04, Median no PE=341.36, p>0.05).  No difference in MVPA between days with and without PE, for normal weight girls (Median PE=44.19, Median no PE=36.27, p>0.05) and for normal weight boys (Median PE=59.79, Median no PE=40.98, p>0.05). No difference in MVPA between days with and without PE, for overweight girls (Median PE=49.29, Median no PE=35.9, p>0.05) and for overweight boys (Median PE=41.17, Median no PE=31.66, p>0.05).  Higher school LPA for days with PE compared to without PE, for normal weight girls (Median PE=119.39, Median no PE=108, p<0.005) and for normal weight boys (Median PE=129, Median no PE=115.25, p<0.01). No difference in school LPA between days with and without PE, for overweight girls (Median PE=111.25, Median no PE=107.25, p>0.05) and for overweight boys (Median PE=131.58, Median no PE=129, p>0.05).  Higher school MVPA for days with PE compared to without PE, for normal weight girls (Median PE=14.9, Median no PE=4.82, p<0.005) and for normal weight boys (Median PE=20, Median no PE=6.59, p<0.005). No difference in school MVPA between days with and without PE, for overweight girls (Median PE=15.66, Median no PE=5.45, p>0.05) and for overweight boys (Median PE=17.29, Median no PE=5.25, p>0.05). |
| Street et al. 2016; USA | N=1,878; grade 9 to 12. | Cross-Sectional | **Homework**  A questionnaire item asked students to report hours spent on homework daily. | **School night sleep duration** was computed from two questions capturing time for going to bed and waking up on school days. These questions are an abbreviated adaptation drawn from the Sleep Timing Questionnaire. Sleep duration was categorized as ≤7 hours insufficient,>7-<9 hours borderline, and ≥9 hours optimal. | **Homework**  Those with ≤1 hour/day of homework had different distributions of insufficient, borderline, and optimal sleep categories compared to those with>3 hours/day of homework (reference group) (p=0.02).  Those with>1-3 hours/day of homework had different distributions of insufficient, borderline, and optimal sleep categories compared to those with>3 hours/day of homework (reference group) (p<0.001). |
| Street et al. 2018; USA | N=1,614; mean age: 16.7 years | Cross-Sectional | **Homework**  A questionnaire item asked students to report hours spent on homework daily. | **School night sleep duration** was computed from two questions capturing time for going to bed and waking up on school days. These questions are an abbreviated adaptation drawn from the Sleep Timing Questionnaire. | **Homework**  Negative association between studying>3 hours, compared to ≤1 hours, and school night sleep duration (β=-0.36, SE=0.12, p<0.05).  Positive association between studying>1-3 hours, compared to ≤1 hours, and school night sleep (β=0.17, SE=0.17, p<0.05). |
| Sun et al. 2014; China | N=734; mean age: 10.8 years | Cross-Sectional | **Homework**  Time spent on homework was surveyed. | **Bedtime routine**  Bedtime routine was assessed with the self-report Chinese version of the Adolescent Sleep Hygiene Scale. Higher scores are indicative of better sleep hygiene.  **Bedroom sharing**  Bedroom sharing was assessed with the self-report Chinese version of the Adolescent Sleep Hygiene Scale. Higher scores are indicative of better sleep hygiene.  **Cognitive sleep hygiene**  Cognitive sleep hygiene was assessed with the self-report Chinese version of the Adolescent Sleep Hygiene Scale. Higher scores are indicative of better sleep hygiene.  **Daytime sleep**  Daytime sleep was assessed with the self-report Chinese version of the Adolescent Sleep Hygiene Scale. Higher scores are indicative of better sleep hygiene.  **Emotional sleep hygiene**  Emotional sleep hygiene was assessed with the self-report Chinese version of the Adolescent Sleep Hygiene Scale. Higher scores are indicative of better sleep hygiene.  **Overall sleep hygiene**  Overall sleep hygiene was calculated as the total score on the self-report Chinese version of the Adolescent Sleep Hygiene Scale. This is a 26-item measure completed across eight subscales: physiological (5 items), cognitive (6 items), emotional (3 items), sleep environment (4 items), daytime sleep (1 item), bedtime routine (1 item), sleep stability (4 items), and bed- or bedroom-sharing (2 items). Higher scores are indicative of better sleep hygiene.  **Physiological sleep hygiene**  Physiological sleep hygiene was assessed with the self-report Chinese version of the Adolescent Sleep Hygiene Scale. Higher scores are indicative of better sleep hygiene.  **Sleep duration**  Average bedtime and waking time on weekday and weekend days were collected through the parent questionnaire and used to calculate the sleep duration.  **Sleep stability**  Sleep stability was assessed with the self-report Chinese version of the Adolescent Sleep Hygiene Scale. Higher scores are indicative of better sleep hygiene. | **Homework**  No difference in bedtime routine sleep hygiene subscore between <1 hour (mean=5.37; 99%CI: 4.87–5.86) and 1-2 hours of homework (mean=4.98; 99%CI: 4.75–5.21) (p>0.05). No difference in bedtime routine sleep hygiene subscore between <1 hour (mean=5.37; 99%CI: 4.87–5.86) and 2-3 hours of homework (mean=4.58; 99%CI: 4.26–4.90) (p>0.05). Higher bedtime routine sleep hygiene subscore for <1 hour (mean=5.37; 99%CI: 4.87–5.86) compared to>3 hours of homework (mean=4.52; 99%CI: 4.14-4.89) (p=0.009).  No difference in bedroom-sharing sleep hygiene subscore between <1 hour (mean=9.92; 99%CI: 8.86–10.99) and 1-2 hours of homework (mean=9.92; 99%CI: 9.48–10.35) (p>0.05). No difference in bedroom-sharing sleep hygiene subscore between <1 hour (mean=9.92; 99%CI: 8.86–10.99) and 2-3 hours of homework (mean=9.56; 99%CI: 9.04–10.09) (p>0.05). No difference in bedroom-sharing sleep hygiene subscore between <1 hour (mean=9.92; 99%CI: 8.86–10.99) and>3 hours of homework (mean=9.35; 99%CI: 8.65-10.06) (p>0.05).  No difference in cognitive sleep hygiene subscore between <1 hour (mean=25.63; 99%CI: 23.26–28.01) and 1-2 hours of homework (mean=25.17; 99%CI: 24.24–26.09) (p>0.05). No difference in cognitive sleep hygiene subscore between <1 hour (mean=25.63; 99%CI: 23.26–28.01) and 2-3 hours of homework (mean=24.52; 99%CI: 23.45–25.58) (p>0.05). No difference in cognitive sleep hygiene subscore between <1 hour (mean=25.63; 99%CI: 23.26–28.01) and (mean=25.34; 99%CI: 24.16-26.52) (p>0.05).  No difference in daytime sleep, sleep hygiene subscore between <1 hour (mean=5.58; 99%CI: 5.17–5.98) and 1-2 hours of homework (mean=5.47; 99%CI: 5.32–5.63) (p>0.05). No difference in daytime sleep, sleep hygiene subscore between <1 hour (mean=5.58; 99%CI: 5.17–5.98) and 2-3 hours of homework (mean=5.44; 99%CI: 5.25–5.64) (p>0.05). No difference in daytime sleep, sleep hygiene subscore between <1 hour (mean=5.58; 99%CI: 5.17–5.98) and>3 hours of homework (mean=5.50; 99%CI: 5.27-5.74) (p>0.05).  No difference in emotional sleep hygiene subscore between <1 hour (mean=14.85; 99%CI: 13.43–16.26) and 1-2 hours of homework (mean=15.41; 99%CI: 15.02–15.81) (p>0.05). No difference in emotional sleep hygiene subscore between <1 hour (mean=14.85; 99%CI: 13.43–16.26) and 2-3 hours of homework (mean=15.08; 99%CI: 14.60–15.57) (p>0.05). No difference in emotional sleep hygiene subscore between <1 hour (mean=14.85; 99%CI: 13.43–16.26) and>3 hours of homework (mean=14.57; 99%CI: 13.88-15.27) (p>0.05).  No difference in overall sleep hygiene score between <1 hour (mean=128.62; 99%CI: 122.69–134.54) and 1-2 hours of homework (mean=126.78; 99%CI: 124.55–129.01) (p>0.05). No difference in overall sleep hygiene score between <1 hour (mean=128.62; 99%CI: 122.69–134.54) and 2-3 hours of homework (mean=123.61; 99%CI: 121.04–126.18) (p>0.05). No difference in overall sleep hygiene score between <1 hour (mean=128.62; 99%CI: 122.69–134.54) and>3 hours of homework (mean=122.43; 99%CI: 119.16-125.70) (p>0.05)  No difference in physiological sleep hygiene subscore between <1 hour (mean=26.25; 99%CI: 24.75–27.75) and 1-2 hours of homework (mean=26.47; 99%CI: 25.98–26.95) (p>0.05). No difference in physiological sleep hygiene subscore between <1 hour (mean=26.25; 99%CI: 24.75–27.75) and 2-3 hours of homework (mean=26.49; 99%CI: 25.98–27.02) (p>0.05). No difference in physiological sleep hygiene subscore between <1 hour (mean=26.25; 99%CI: 24.75–27.75) and>3 hours of homework (mean=26.14; 99%CI: 24.45-26.84) (p>0.05).  There was a negative association between homework duration and sleep duration (β=-0.23, p<0.001).  No difference in sleep stability sleep hygiene subscore between <1 hour (mean=18.65; 99%CI: 16.47–20.84) and 1-2 hours of homework (mean=17.2; 99%CI: 16.44–17.95) (p>0.05). Higher sleep stability sleep hygiene subscore for <1 hour (mean=18.65; 99%CI: 16.47–20.84) of homework, compared to 2-3 hours of homework (mean=15.97; 99%CI: 15.02–16.93) (p=0.006). Higher sleep stability sleep hygiene subscore for <1 hour (mean=18.65; 99%CI: 16.47–20.84) of homework, compared to>3 hours of homework (mean=15.57; 99%CI: 14.51-16.63) (p=0.002). |
| Tang and Patrick 2018; USA | N=40,389; grade 8 and 10. | Cross-Sectional | **Homework**  Time spent on homework was surveyed. | **Play electronic video games** Students reported the number of hours per week they spent “playing electronic games on a computer, television, phone, or other device”.  **Social networking**  Students reported the number of hours per week they spent “texting on a cell phone”, “talking on the cell phone”, “video chatting (Skype, etc.)” and “visiting social networking websites like Facebook, Twitter, Instagram, etc.”.  **Talking on phone**  Students reported the number of hours per week they spent “talking on the cell phone”.  **Television on a weekday**  Students reported the average number of hours spent watching television on an average weekday.  **Television on a weekend day**  Students reported the average number of hours spent watching television on an average weekend day.  **Texting**  Students reported the number of hours per week they spent “texting on a cell phone”.  **Video chatting**  Students reported the number of hours per week they spent “video chatting (Skype, etc.)”. | **Homework**  Higher levels of playing electronic video games for <5 hours of homework time (mean [SE]=4.57 [0.02]), compared to ≥5 hours of homework time (mean [SE]=4.43 [0.04]) (p<0.01).  No difference for social networking between <5 hours of homework time (mean [SE]=4.27 [0.03]), and ≥5 hours of homework time (mean [SE]=4.24 [0.03]) (p>0.05).  Higher levels of talking on the phone for <5 hours of homework time (mean [SE]=3.02 [0.02]), compared to ≥5 hours of homework time (mean [SE]=2.92 [0.03]) (p<0.01).  Higher levels of television on a weekday for <5 hours of homework time (mean [SE]=4.03 [0.02]), compared to ≥5 hours of homework time (mean [SE]=3.58 [0.03]) (p<0.01).  No difference for television on a weekend between <5 hours of homework time (mean [SE]=4.15 [0.02]) and ≥5 hours of homework time (mean [SE]=4.13 [0.02]) (p>0.05).  No difference for texting between <5 hours of homework time (mean [SE]=4.54 [0.03]) and ≥5 hours of homework time (mean [SE]=4.54 [0.03]) (p>0.05).  No difference for video chatting between <5 hours of homework time (mean [SE]=2.45 [0.02]) and ≥5 hours of homework time (mean [SE]=2.39 [0.03]) (p>0.05). |
| Wang et al. 2020; China | N=176,428; mean age: 12.1 years | Cross-Sectional | **Homework**  Weekday time spent on homework was surveyed. | **Reading in bed**  Students reported reading in bed in a multiple-choice question indicating the frequency of this behaviour.  **Weekday screen time**  Students reported screen time in a multiple-choice question indicating the frequency of this behaviour.  **Weekend screen time**  Students reported screen time in a multiple-choice question indicating the frequency of this behaviour. | **Homework**  Positive association between math homework time on weekdays and reading in bed (Correlation r=0.053, p<0.01).  Negative association between math homework time on weekdays and weekday screen time (Correlation r=-0.016, p<0.01).  Positive association between math homework time on weekdays and weekend screen time (Correlation r=0.027, p<0.01). |
| Widome et al. 2019; USA | N=2,134; Grade 9. | Cross-Sectional | **Homework** Time spent studying or doing homework before school and on school nights were assessed using a questionnaire. | **Sleep duration**  Sleep duration was calculated from two items adapted from the Teen Sleep Habits Survey regarding usual bedtime and waking time. | **Homework**  No association between doing homework before school and sleep duration (p=0.089).  Positive association between prevalence of homework on school nights and sleep duration (p=0.0001). |
| Yeo et al. 2019; Singapore | N=2,313; age: 16 years | Cross-Sectional | **Homework**  Homework or studying time were assessed using a questionnaire. | **School day bedtime**  Participants self-reported their bedtime. | **Homework**  Those with <3 hours of studying on school days, had earlier bed times than those studying for ≥3 hours (main effect of  studying duration: F=41.6, p < 0.001). |
